# All-silicon multidimensionally-encoded optical physical unclonable functions for integrated circuit anti-counterfeiting

**DOI:** 10.1038/s41467-024-47479-y

**Published:** 2024-04-13

**Authors:** Kun Wang, Jianwei Shi, Wenxuan Lai, Qiang He, Jun Xu, Zhenyi Ni, Xinfeng Liu, Xiaodong Pi, Deren Yang

**Affiliations:** 1 State Key Laboratory of Silicon and Advanced Semiconductor Materials & School of Materials Science and Engineering, Zhejiang University, Hangzhou, Zhejiang 310027 China; 2 CAS Key Laboratory of Standardization and Measurement for Nanotechnology, National Center for Nanoscience and Technology, Beijing, 100190 China; 3 State Key Laboratory for Superlattices and Microstructures, Institute of Semiconductors, Chinese Academy of Sciences, Beijing, 100083 China; 4 School of Electronic Science and Engineering & National Laboratory of Solid State Microstructures, Nanjing University, Nanjing, Jiangsu 210093 China; 5 School of Microelectronics, Nantong University, Nantong, Jiangsu 226019 China; 6 Institute of Advanced Semiconductors, ZJU-Hangzhou Global Scientific and Technological Innovation Centre, Zhejiang University, Hangzhou, Zhejiang 311215 China

**Keywords:** Metamaterials, Quantum dots, Applied optics

## Abstract

Integrated circuit anti-counterfeiting based on optical physical unclonable functions (PUFs) plays a crucial role in guaranteeing secure identification and authentication for Internet of Things (IoT) devices. While considerable efforts have been devoted to exploring optical PUFs, two critical challenges remain: incompatibility with the complementary metal-oxide-semiconductor (CMOS) technology and limited information entropy. Here, we demonstrate all-silicon multidimensionally-encoded optical PUFs fabricated by integrating silicon (Si) metasurface and erbium-doped Si quantum dots (Er-Si QDs) with a CMOS-compatible procedure. Five in-situ optical responses have been manifested within a single pixel, rendering an ultrahigh information entropy of 2.32 bits/pixel. The position-dependent optical responses originate from the position-dependent radiation field and Purcell effect. Our evaluation highlights their potential in IoT security through advanced metrics like bit uniformity, similarity, intra- and inter-Hamming distance, false-acceptance and rejection rates, and encoding capacity. We finally demonstrate the implementation of efficient lightweight mutual authentication protocols for IoT applications by using the all-Si multidimensionally-encoded optical PUFs.

## Abstract (web summary)

The researchers introduce an all-silicon optical PUF that enhances IoT device security through CMOS-compatible fabrication, showcasing five unique optical responses per pixel for advanced authentication and high information entropy.

## Introduction

The rapid expansion of Internet of Things (IoT) devices has been demanding secure management of sensitive personal information^1,2^. However, the widespread use of counterfeit integrated circuits in networked devices poses a challenge to seamless IoT integration and raises concerns about the security and reliability of the electronics supply chain^3^. Asymmetric cryptography that is exemplified by the renowned Rivest–Shamir–Adleman (RSA) encryption, offers a robust mechanism for information security by leveraging the computational complexity of factoring large integers^4^. Nevertheless, this complexity becomes surmountable with quantum computing^5^, thus motivating the exploration of alternative approaches. Symmetric cryptography operates on the premise that entities share a private key, typically stored in nonvolatile memory, enabling mutual encryption and decryption of messages^6^. However, the stored key can be compromised by physical and side-channel attacks^7^. To address the aforementioned vulnerabilities, physically unclonable functions (PUFs) generating unique fingerprints from intrinsic random variations are now vigorously explored^8–10^. Among all types of PUFs, optical PUFs have become preferable due to their high output complexity and resilience against erratic IoT power supplies^11,12^.

Up to now, optical PUFs have been primarily based on single-dimensional responses such as micropattern imaging^13,14^, Raman scattering^15^, fluorescent lifetime^16^, and fluorescent intensity^17,18^ within each pixel. These single-dimensionally-encoded optical PUFs typically generate one response per pixel, imposing fundamental limits on the encoding capacity^19^ and mutual authentication protocols^20^. As a consequence, multidimensionally-encoded optical PUFs that are capable of producing multiple responses within a single pixel have attracted growing interest. Examples include lanthanide(III)-doped zeolites^21^, fluorescent proteins^22^, gap-enhanced nanoparticles (NPs)^23^, plasmonic NPs^24^, organic molecules^25^, perovskites^26^, and nanorods^27^. However, these optical PUFs are plagued by their inherent weaknesses, such as signal cross-talk and limited spatial resolution, largely owing to the incorporation of heterogeneous materials in anti-counterfeiting inks. This underscores the need for the development of multidimensionally-encoded optical PUFs that can leverage the unique properties of single-material systems (e.g., Ag nanoislands^28^, nanocluster/graphene hybrids^29^, Au networks^30^, block copolymer self-assembly^20^, diamond microparticles^31^, and carbon dots^8^). Nevertheless, many reported multidimensionally-encoded optical PUFs involve materials that are not compatible with the standard silicon (Si) complementary metal-oxide-semiconductor (CMOS) platform. The incompatibility with CMOS manufacturing impedes the scalable integration of these PUFs to combat the counterfeiting of integrated circuits for IoT systems^1^. Moreover, existing multidimensionally-encoded optical PUFs suffer from low information entropy due to their limited optical responses within a single pixel^7,8,10,20,32–34^. This leads to high false-acceptance rates (FARs) induced by the overlapping or indistinguishable features between genuine and counterfeit samples^19,23^. Therefore, it is highly desired to make a single pixel that is capable of manifesting more optical responses, enabling high information entropy for optical PUFs.

Optical metasurfaces composed of artificially engineered subwavelength building blocks^35^ hold great promise for enhancing the performance of multidimensionally-encoded optical PUFs by leveraging the optical properties of quantum dots (QDs)^36,37^. It has been recently demonstrated that erbium-doped Si quantum dots (Er–Si QDs) exhibit dual near-infrared (NIR) emissions^38^, enabling excellent detection of signals against background noises^31^. Furthermore, Si substrates have been proven to be exceptional optical metasurfaces by providing various nanostructures^39^. Therefore, it is inspired that the integration of a metasurface based on a Si substrate with Er–Si QDs may render all-Si, CMOS-compatible, multidimensionally-encoded optical PUFs with high information entropy, fulfilling the requirements of integrated circuit anti-counterfeiting.

In this work, we present the fabrication of all-Si multidimensionally-encoded optical PUFs by integrating a metasurface that is enabled by the copper (Cu)-nanoparticle-assisted anisotropic etching of a Si substrate with Er–Si QDs. Such an integration enables five in-situ optical responses encompassing micropattern imaging (R_1_), the photoluminescence (PL) intensity of Si QDs (R_2_), the PL intensity of Er^3+ ^(R_3_), the PL wavelength of Si QDs (R_4_), and the PL lifetime of Si QDs (R_5_) to be realized within a single pixel. Hence, the all-Si multidimensionally-encoded optical PUFs have ultrahigh information entropy (up to 2.32 bits/pixel). Finite-difference time-domain (FDTD) simulations show that the Si metasurface and Er–Si QDs are robustly coupled owing to the radiation field and Purcell effect, giving rise to position-dependent optical responses. The bit uniformity, similarity, intra-Hamming distance (Intra-HD), inter-Hamming distance (Inter-HD), false-acceptance rate (FAR), false-rejection rate (FRR), and encoding capacity of the all-Si multidimensionally-encoded optical PUFs are calculated. Efficient, lightweight mutual authentication protocols for IoT are finally demonstrated by using the current all-Si multidimensionally-encoded optical PUFs.

## Results

### Fabrication of all-Si multidimensionally-encoded optical PUFs

**Fig. 1:**
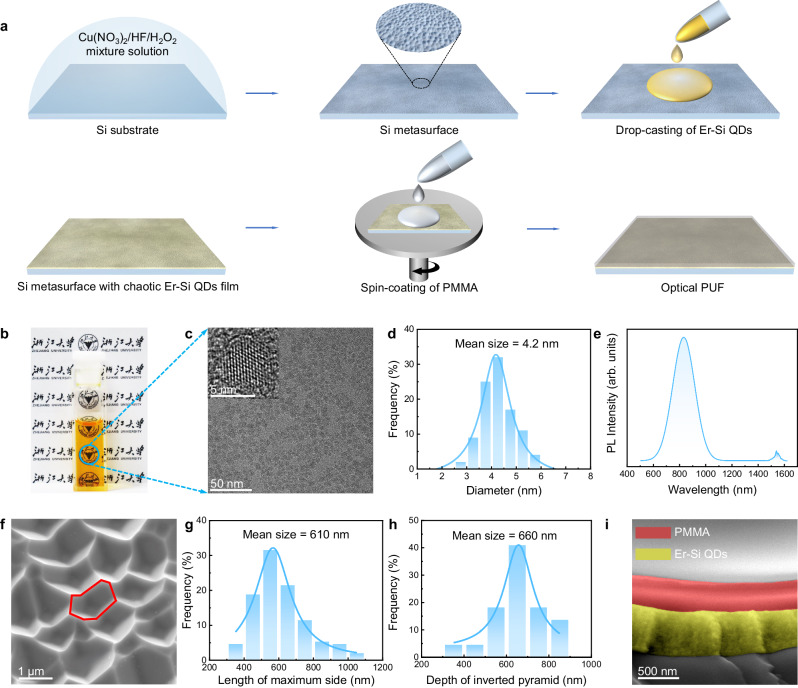
Preparation and structural characterization of an all-Si multidimensionally-encoded optical PUF. **a** Schematic illustration of the fabrication process of an all-Si multidimensionally-encoded optical PUF. **b** Photograph of a solution of Er–Si QDs. **c** Transmission electron microscopy (TEM) image of Er–Si QDs. The inset displays a high-resolution TEM image. **d** Size distribution of Er–Si QDs. **e** PL spectrum of Er–Si QDs. **f** Scanning electron microscopy (SEM) image at a 30° side view of the random array of inverted pyramids. The red lines depict the sides of an inverted pyramid. **g** Distribution of the maximum length of the sides of an inverted pyramid. **h** Distribution of the depth of an inverted pyramid. For the statistical analysis presented in Fig. 1g, h, 50 top-view SEM images with each measuring the area of 20 μm × 20 μm are used. **i** Cross-sectional SEM image of an all-Si multidimensionally-encoded optical PUF.

The fabrication process of all-Si multidimensionally-encoded optical PUFs is schematically depicted in Fig. 1a. A Si metasurface is initially prepared by etching a Si substrate with the mixture of Cu(NO_3_)_2_/HF/H_2_O_2_ containing Cu nanoparticles. This is followed by the deposition of Er–Si QDs by drop casting, which is synthesized through a combination of nonthermal plasma and wet chemistry^38^. The resultant PUF is then encapsulated by spin-coating poly(methyl methacrylate) (PMMA). It is noteworthy that the scalability and cost-effectiveness of the fabrication process are paramount for the practical deployment of PUF-based anti-counterfeiting labels. The processing techniques adopted in this work, such as nonthermal plasma synthesis^40,41^, wet etching^42–44^, drop casting^45^, and spin coating^46,47^ have already been widely used in the CMOS industry. This means that our fabrication process could be readily carried out based on the existing CMOS manufacturing infrastructure, which is crucial for large-scale production. Moreover, cost analysis reveals that the total cost for the fabrication of each PUF label is approximately ~ 10^−5^ USD, highlighting the economic viability of the current approach for widespread applications (Supplementary Table 1 and Supplementary Note 1).

Figure 1b shows a photograph of Er–Si QDs dispersed in toluene. The low-resolution TEM image (Fig. 1c) shows that Er–Si QDs are basically spherical. The high-resolution TEM image of a typical Er–Si QD (the inset of Fig. 1c) further reveals the good crystallinity of the Er–Si QDs. Statistical analysis indicates that Er–Si QDs have a mean size of ~ 4.2 nm with a standard deviation of 0.6 nm (Fig. 1d). Figure 1e shows that the Er–Si QDs exhibit PL peaks at 830 nm and 1540 nm, which correspond to the band-to-band transition of Si QDs and the ^4^I_13/2_ → ^4^I_15/2_ transition of Er^3+^, respectively^48^. A 30° side view of the Si metasurface obtained by SEM is illustrated in Fig. 1f (more SEM images of the Si metasurface are shown in Supplementary Fig. 1a, b). Randomly distributed inverted pyramids obtained with the Cu-nanoparticle-assisted anisotropic etching^49^ are readily observed. Statistical analysis on the maximum length of the sides of an inverted pyramid and that on the depth of an inverted pyramid are plotted in Fig. 1g, h, respectively. These results indicate that the average length of the longest side and depth of an inverted pyramid are ~ 610 nm and 660 nm, respectively. Such a subwavelength disorder serves as a nano-antenna, enabling the modulation of light emissions from Er–Si QDs through scattering, reflection, absorption, and localization^26^. A cross-sectional SEM image (Fig. 1i) indicates that the Si metasurface is well covered by Er–Si QDs. Please note that the PMMA is employed to protect Er–SiQDs from degradation in water (Supplementary Fig. 1c), which is transparent to the light emitted from Er–Si QDs (Supplementary Fig. 1d).

### Encoding based on in-situ multidimensionally-encoded optical responses

**Fig. 2:**
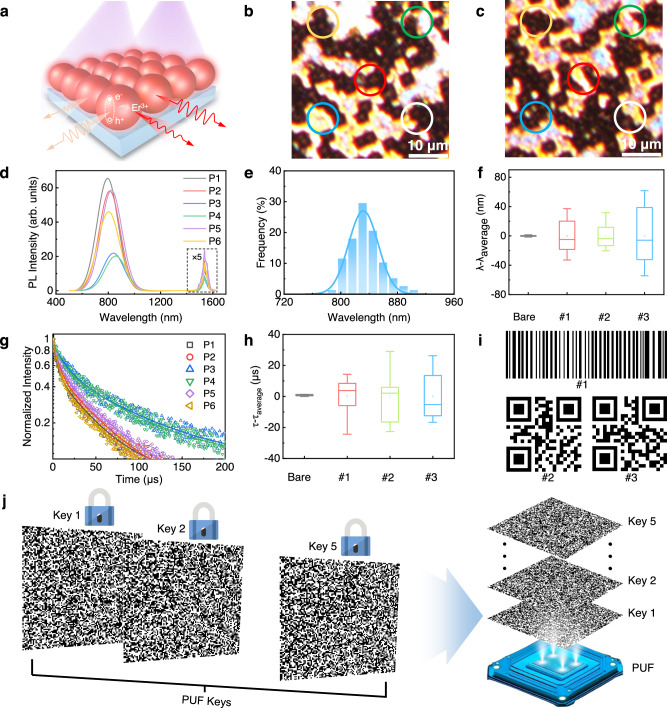
All-Si multidimensionally-encoded optical PUF encryption features. **a** Illustration depicting the working principle of the PUF. Top-view optical microscopy images of a PUF at positions 1 (**b**) and 2 (**c**). The overlaid circles highlight the differences between the random micropatterns. **d** PL spectra of the PUF at six different positions. The PL peaks at 1540 nm are magnified by a factor of five. **e** Statistical distribution of the wavelength of the PL from Si QD. **f** Demonstration of the random PL wavelength of Si QDs by using 20 positions per sample. *λ*_average_ is the average value of the PL wavelength. (*λ*–*λ*_average_) quantifies the variation of the PL wavelength. **g** The PL lifetime of Si QDs at six different positions. **h** Demonstration of the random PL lifetime of Si QDs by using 20 positions per sample. *τ*_average_ is the average value of the PL lifetime. (*τ*–*τ*_average_) quantifies the variation of the PL lifetime. **i** Optical key generation from various samples with distinct PL-wavelength thresholds. **j** Schematic image of five security keys extracted from a single PUF. All boxplots illustrate the interquartile range (IQR), extending from the first to the third quartile, with a central line denoting the median. Whiskers extend from the quartiles to the minimum and maximum data points. Hollow square points indicate the mean values.

Figure 2a schematically shows the working principle of an all-Si multidimensionally-encoded optical PUF. Upon optical illumination (referred to as the “challenge”), the PUF generates randomized optical signals (referred to as the “responses”), which are dependent on the position of the optical illumination. The first response (R_1_) is recorded as the image of the micropattern at the illumination position. When the illumination position changes, the micropattern changes as well, as indicated by the optical microscopy images (Fig. 2b, c). Figure 2d presents the PL spectra of the PUF acquired at different positions. The PL intensity of Si QDs (R_2_) and that of Er^3+^ (R_3_) are both dependent on position. The stochastic distribution of R_2_ and R_3_ across the PUF is further demonstrated in Supplementary Fig. 2a and b. The dependence of R_1_, R_2_ and R_3_ on position may primarily arise from the synergistic interplay between the inherent structural disorder of the Si metasurface and the random spatial distribution of Er–Si QDs^31^. In particular, the greater randomness of R_3_ derived from the Si metasurface compared to those from the bare Si substrate (Supplementary Fig. 3) well indicates the interplay between the Si metasurface and R_3_. Additionally, Fig. 2d shows a position-dependent wavelength change of the PL associated with the band-to-band transition of Si QDs (R_4_), consistent with the influence of the subwavelength structure of the Si metasurface on the emission wavelength of Si QDs^36^. The stochastic distribution of R_4_ across various positions is illustrated in Fig. 2e. To ensure that the randomized R_4_ is not derived from the measurement error or noise, Er–Si QDs have also been drop-coated onto an unpatterned bare Si substrate. It is found that the position-dependent fluctuation of the PL wavelength for Si QDs is rather small, in contrast to those of the PL wavelength for Si QDs in three randomly selected PUFs (Fig. 2f), highlighting the influence of the Si metasurface on the emission wavelength of Si QDs.

The position-dependent PL lifetime of Si QDs (R_5_) shown in Fig. 2g substantiates the optical coupling between the Si metasurface and Er–Si QDs. In addition, the stochastic distribution (Supplementary Fig. 2c) and random generation (Fig. 2h) of R_5_ across the PUF further validate the interaction between the Si metasurface and Er–Si QDs. Consequently, five distinct optical responses could be obtained within a single PUF. It should be stressed that these five optical responses are generated at any individual position of a single PUF, endowing the PUF with the capability of achieving in situ multidimensionally encoded responses. Hence, an ultrahigh information entropy (up to 2.32 bits/pixel) is obtained (Supplementary Note 2), which is superior to those of PUFs based on alternative technologies^7,8,10,13,20,31–34,50–54^ (Supplementary Fig. 4). Each of the five optical responses can be employed to generate a key in a popular form. For example, R_4_ of three PUFs is transformed into a binary barcode or quick-response (QR) code by introducing a predefined threshold (Fig. 2i, Supplementary Note 3). In contrast to a conventional PUF that could only generate a single key, five keys are generated in a single PUF (Fig. 2j), enabling mutual authentications. Users can also readily select an authentication key tailored to their specific requirements. Moreover, the combination of the multiple authentication keys can facilitate robust security through multifactor authentication.

### The coupling mechanism between the Si metasurface and Er–Si QDs

**Fig. 3:**
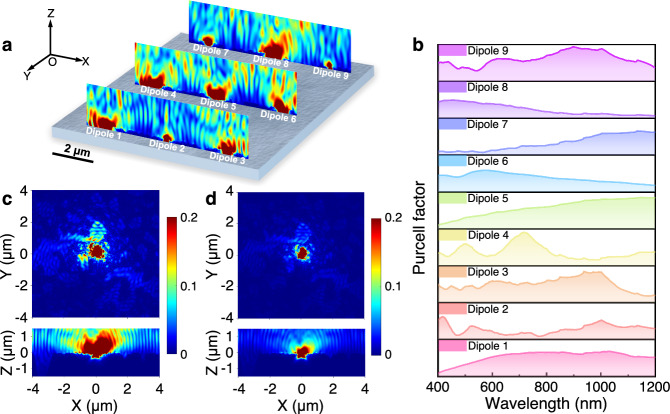
Simulated position-dependent optical behaviors in an all-Si multidimensionally-encoded optical PUF. **a** Radiation field distribution at the wavelength of 830 nm for nine dipole emitters across the Si metasurface. **b** Comparison of Purcell factors for the nine dipole locations. **c** Radiation field intensity distributions in the XY plane (top) and XZ plane (bottom) for a single dipole at its original position of the Si metasurface. **d** Radiation field intensity distributions in the XY plane (top) and XZ plane (bottom) for the same dipole shifted 10 nm from its original position at the Si metasurface.

To figure out the coupling mechanism between the Si metasurface and Er–Si QDs, FDTD simulations are employed. These Er–Si QDs contain two emitters: Er^3+^ and Si QDs. Owing to the shielding of the 4*f* shell orbital by the outer 5*s* and 5*p* orbitals for Er^3+^ (ref. ^55^), the PL wavelength and lifetime of Er^3+^ in Er–Si QDs exhibit rather weak sensitivity to environmental changes (Supplementary Fig. 5), limiting their use for anti-counterfeiting. Thus, the following FDTD simulations focus on the optical responses related to the band-to-band transitions of Er–Si QDs (i.e., the PL emission of Si QDs), particularly in relation to the interaction with the Si metasurface. Using 3D FDTD simulations, we determine the spatial distribution of the radiation-field intensity and Purcell factor for the light emission induced by the band-to-band transition of Er–Si QDs on Si metasurface. The Er–Si QDs were conceptualized as electric dipoles, and the structure of the Si metasurface was constructed from its SEM image. Figure 3a displays the radiation field distributions of nine dipoles positioned on the Si metasurface for light emission at the wavelength of 830 nm. It is clear that the near-field distributions of these dipoles exhibit heterogeneity within the plane parallel to the Si metasurface (Supplementary Fig. 6a). Please note that they are remarkably uniform within the plane parallel to an unpatterned bare Si surface (Supplementary Fig. 6b). It is the subwavelength disorder of the Si metasurface that acts as nano-antennas, enabling the manipulation of dipole emission via mechanisms such as scattering, reflection, absorption, and localization^26^. Variations in the radiation field distribution stem from different coupling strengths between the dipoles and metasurface at different positions.

Figure 3b shows the position-dependent Purcell factors for the nine dipoles. The variation in the Purcell factor across different positions leads to varying enhancements in the radiative recombination rate of Si QDs, resulting in variations in R_4_ (ref. ^56^). Since the radiative lifetime is reciprocal to the radiative recombination rate^57^, R_5_ also changes with position. Remarkably, the distributions of the radiation field intensity (Fig. 3c, d) and Purcell factor (Supplementary Fig. 6c) show variation even between two dipoles positioned merely 10 nm apart on the Si metasurface. These changes create a unique fingerprint for each individual PUF, forming the basis for anti-counterfeiting. Our current simulations highlight the capability of the PUF to provide fingerprint patterns with an extraordinary theoretical spatial resolution of 10 nm, enabled by the intrinsic dependence of the spontaneous emission of a quantum emitter on the local density of states (LDOS) within a nanoscale environment^37^. We should note that our test limits the spatial resolution of the PUF to around 2 μm, otherwise, a much higher spatial resolution could be achievable by using miniaturized photonic integrations.

To delve more deeply into the role of the Si metasurface in the generation of optical responses, simulations have been conducted to examine the distributions of the reflected field intensity associated with an incident plane wave with a wavelength of 830 nm. The results show intricate and disordered optical patterns characterized by significantly changed brightness and darkness (Supplementary Fig. 6d). Furthermore, the interaction between the ultraviolet (UV) excitation and the Si metasurface increases the stochasticity of the optical responses. Under the illumination at the wavelength of 405 nm, the metasurface exhibits tapestry-like hotspots of the local exciting field (Supplementary Fig. 6e) owing to the diffractive coupling^39^ and the interaction with photonic modes^58,59^. Both the stochastic reflected field and exciting field demonstrate that the optical response of the Si metasurface encompasses multiple modes of propagation and interaction, improving the complexity and security of our PUFs. Therefore, we would like to mention that the multidimensionally-encoded optical responses and the unpredictability of complex challenge-response pairs (CRPs) inherent to the PUF intrinsically confound machine learning-based modeling attacks^34,60^.

### Performance of an all-Si multidimensionally-encoded optical PUF

**Fig. 4:**
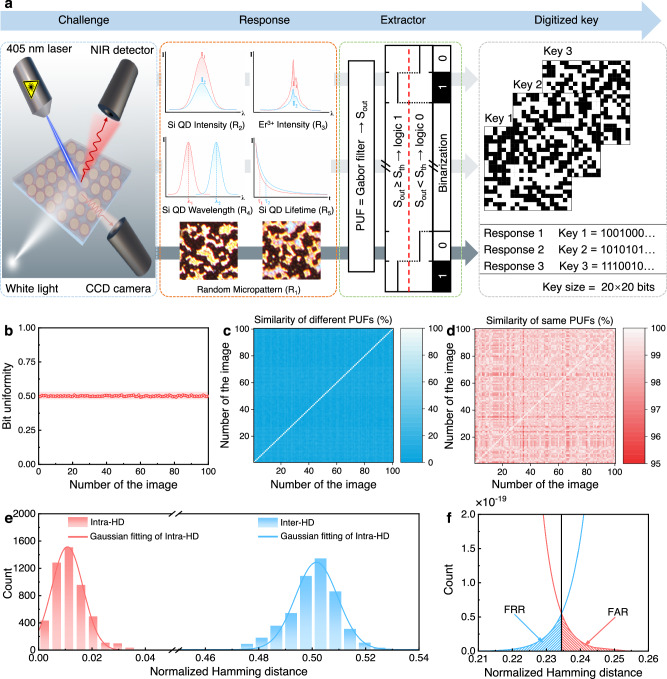
Generated multiple security keys and features of a PUF based on the PL wavelength of Si QDs (R_4_). **a** Challenge-response authentication process for the PUF. **b** Occurrence probability of “1” in binary bits extracted from 100 images. **c** Pairwise match of 100 different PUFs using per pixel binary encoding of images acquired from the different positions. **d** Pairwise match of 100 same PUFs using per pixel binary encoding of images acquired from the same position. **e** Distribution of normalized Hamming distance (HD) for Inter-HD and intra-HD. **f** Magnified Inter-HD and Intra-HD.

Figure 4a depicts the “challenge-response” authentication process employed by an all-Si multidimensionally-encoded optical PUF. At the beginning of challenge generation, common light sources such as a white LED and a UV laser are used as challenges. An array of responses including R_1_–R_5_ are subsequently recorded and represented as the responses. The following extraction is carried out by making the PUF function as a distinctive random-number generator. This is facilitated by its unique interaction with the challenges. A digitalization process is then performed, which compares with pre-established threshold values to generate response bits and the final cryptographic keys (Supplementary Fig. 7).

To quantitatively evaluate the anti-counterfeiting performance of the PUF, we analyze its characteristics, including bit uniformity, similarity, Intra-HD, Inter-HD, FAR, FRR, and encoding capacity. Here, we take R_4_ as an example. Each PUF is designed to be represented by a square image with a size of 40 × 40 μm^2^. Metallic marks (Supplementary Fig. 8) are used to simplify location tracking during recurrent measurements. Prior to analysis, the discrepancies in the image arising from device vibrations and other factors are corrected by utilizing the principle of phase correlation (Supplementary Note 4)^61,62^. The discrepancies are manifested in the frequency domain as follows:

1 $$F\left(u,\nu \right)=G(u,\nu )\exp \left[2\pi i\left(\frac{{udx}}{M}+\frac{\nu {dy}}{N}\right)\right]$$

where $F\left(u,\nu \right)$ represents the Fourier transform of the reference image, and $G(u,\nu )$ denotes the Fourier transform of the images requiring correction. The term $\exp \left[2\pi i\left(\frac{{udx}}{M}+\frac{\nu {dy}}{N}\right)\right]$ represents the correction factor, where *dx* and *dy* are the displacements in the x and y directions, respectively, and *M* and *N* are the dimensions of the image in the x and y directions, respectively. Once the correction is accomplished, the speeded-up robust features (SURF) algorithm^63^ is employed to verify the location of identical feature regions within the image.

In a single PUF, every pixel carries an equal chance of being in either a 0 or 1 state. Thereby, achieving a balanced distribution of 0 and 1 states becomes crucial for optimizing the capacity of producing a varying array of random binary-code combinations^64^. The concept of Hamming weight pertains to the count of nonzero elements within the PUF, particularly pertaining to the presence of “1” in the system. The Hamming weight serves as a metric for determining the level of bit uniformity (Supplementary Equation 5). Given that each bit in the binary array has an equiprobable chance of being either 0 or 1, an optimal bit uniformity value should converge to 0.5 (ref. ^8^). Figure 4b illustrates the probability distribution of “1” occurrences within the binary bits extracted from 100 images, unveiling an approximate value of 0.5. This observation signifies the emergence of a bitwise maximum entropy code^65^.

Subsequently, the similarity among the images of 100 PUFs is assessed to explore the uniqueness of the PUF response, as demonstrated in Fig. 4c (Supplementary Eq. 6). Remarkably, the similarities for all images are less than 15%, conclusively validating the exceptional capability of the PUFs in generating perceptibly distinct optical responses. To ascertain the repeatability and robustness of the optical responses, a series of 100 PUF images are captured at different time intervals, all obtained from the same position on a single PUF. The depiction in Fig. 4d provides evidence of the repeatability and robustness of the optical responses, with similarities exceeding 95% across all images. To elucidate the authentication results, we employed similarity distribution histograms to visualize the results (Supplementary Fig. 9). Remarkably, the similarity values between different PUFs and the same PUFs are clearly segregated, exhibiting a substantial gap of approximately 80%. This notable differentiation between responses originating from the same PUFs and those from different PUFs establishes a sturdy foundation for reliably verifying device or individual authenticity using our PUF-based authentication systems^27^.

In addition, we proceeded to measure the Intra-HD (Supplementary Eq. 7) within the 100 same PUFs, alongside the Inter-HD (Supplementary Eq. 8) across the 100 different PUFs, as exemplified in Fig. 4e. The Intra-HD, reflecting the variation within the same PUFs, manifests an exceedingly narrow Gaussian distribution spanning from 0.0025 to 0.0425, with an average of 0.0119. Impressively, this average value approaches the ideal theoretical value of 0, denoting minimal dissimilarity between the same PUFs. This minimal dissimilarity between the same PUFs likely stems from minute vibrations inherent to the optical system, along with the associated noise originating from the charge-coupled device (CCD)^23^. In contrast, the Inter-HD follows a Gaussian distribution centered at 0.5004, nearing the optimal value of 0.5. This observation underscores the remarkable device randomness exhibited by all PUF instances^31^. To assess the performance of the authentication system, FAR and FRR were evaluated from the Intra-HD and Inter-HD measurements. The overlapping regions between the Intra-HD and Inter-HD can be categorized as FAR and FRR. The region of overlaps constitutes the FAR and FRR, with the former occupying the false positive intersection and the latter residing in the false negative domain^66^, as depicted in Fig. 4f. Impressively, the resulting FAR and FRR values are computed as 2.4232 × 10^−22^ and 3.1076 × 10^−22^, respectively. These infinitesimal values signify the near-zero occurrence of false acceptances and false rejections when PUFs are employed for authentication purposes. Furthermore, by calculating the average normalized HD across the digitized keys of the five optical responses within each PUF, we evaluate the uniqueness of individual digitized keys (Supplementary Fig. 10). Remarkably, across the 100 different PUFs examined, a mean normalized HD value of 0.4999 is obtained, affirming the uniqueness of individual digitized keys within each PUF^22^.

Apart from the PL wavelength related to Si QDs (R_4_), the anti-counterfeiting performance of the other optical responses within the PUF is depicted in Supplementary Figs. 11–14. Table 1 presents an overview of the anti-counterfeiting performance metrics for all PUF optical responses. The results validate the ability of the PUFs to generate unique cryptographic keys, achieving negligible FAR and FRR. In addition, the feasibility of extracting optical responses using low-cost, user-friendly devices is essential for practical application. To this end, we have utilized standard smartphones in conjunction with portable microscopes to capture the R_1_ of our PUF labels, as depicted in Supplementary Fig. 15, demonstrating excellent anti-counterfeiting performance. With the progress of portable spectrometer technology, the ongoing improvements of smartphone cameras, and the evolution of fluorescence imaging techniques based on pulse sampling^67^ and frequency domain^68^, we anticipate that the potential of extracting all five optical responses by using even more affordable and user-friendly devices may be realized.

**Table 1:** The anti-counterfeiting performance and readout accessibility of our PUF for various optical responses

Response	Bit uniformity	Intra-HD	Inter-HD		FAR	FRR	Readout		
							Time		Equipment
R_1_	0.4989	0.0125	0.4989		2.0538 × 10^−21^	1.4709 × 10^−21^	1 s		Optical microscopy
R_2_	0.4987	0.0062	0.5005		2.1088 × 10^−36^	5.3115 × 10^−36^	40 s		Confocal Raman System
R_3_	0.4985	0.0063	0.4998		4.7162 × 10^−31^	8.0269 × 10^−31^	200 s		
R_4_	0.5006	0.0120	0.5004		2.4232 × 10^−22^	3.1076 × 10^−22^	40 s		
R_5_	0.4991	0.0106	0.5017		1.3949 × 10^−16^	3.0477 × 10^−16^	40 s		

In addition, we have computed the theoretical encoding capacity of the PUF. The theoretical encoding capacity represents the number of CRPs that can be generated and is expressed as *c*^*s*^, where *c* represents the number of optical responses per pixel and *s* denotes the key size^22,64^. The PUF in our study possesses a *c* value of 5, consisting of five optical responses. Accordingly, we image an area of 20 × 20 pixels, providing a theoretical encoding capacity of 5^400^ (i.e., 3.8726 × 10^279^). This is significantly larger than the encoding capacity (~ 10^20^) of rudimentary PUFs, manifesting compelling resistance to counterfeiting attempts^19,30^. Furthermore, the theoretical encoding capacity can be enhanced by expanding the key size, leveraging the remarkable theoretical spatial resolution of the PUF.

We have also validated the robustness of our PUFs to heating, laser exposure, abrasion, and UV radiation (Supplementary Figs. 16–19). Thermal stability is examined by heating the PUFs to 100 °C for 1 h and comparing pre-heating and post-heating images. The average similarity between pre- and post-heating images exceeds 94% for each optical response (Supplementary Fig. 16). Photostability is verified by acquiring 100 consecutive images under 405 nm laser illumination. The average similarity remains above 98% for all-optical responses (Supplementary Fig. 17). Mechanical durability is evaluated by abrading the PUFs with quartz sands. The average post-abrasion similarity is greater than 94% for each type of signal (Supplementary Fig. 18). Finally, UV stability is tested by exposing the PUFs to 365 nm UV radiation at 10 W for 1 h. The average similarity remains above 95% post-exposure (Supplementary Fig. 19). All these results validate the remarkable stability of our all-Si multidimensionally-encoded PUFs under thermal, laser, mechanical, and UV conditions.

### Authentication of the PUFs

**Fig. 5:**
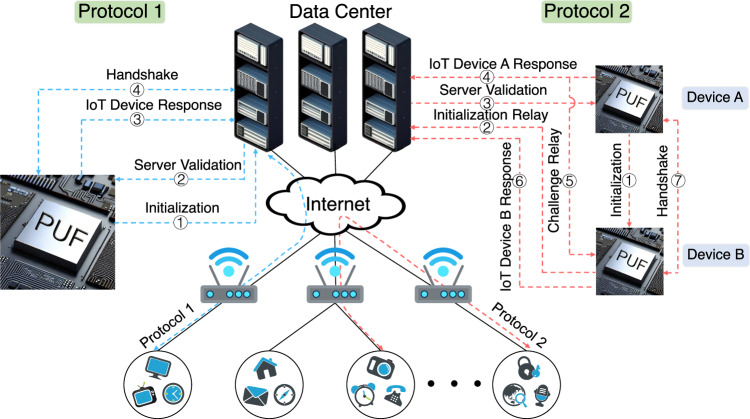
Mutual authentication protocols enabled by the all-Si multidimensionally-encoded optical PUF. Protocol 1: device-server mutual authentication. Authentication for the IoT device initiates with the transmission of its ID and a random number to the server (①). The server validates the device, formulates an encrypted message, and sends it back along with codes (②). The device responds, validates the data, and establishes new codes for mutual authentication (③). The server verifies the message, and if successful, mutual authentication is achieved (④). Protocol 2: device-device mutual authentication. The authentication process begins with IoT device A sending its ID and a random number to IoT device B (①). Device B relays the IDs and numbers to the server (②). The server retrieves suitable CRPs and generates encrypted messages for device A (③). Device A responds by decrypting messages, verifying integrity, and sending an encrypted message to the server (④). The server updates the associated CRP. A challenge relay occurs (⑤), and device B responds similarly (⑥). Finally, a validation and handshake process takes place, completing the mutual authentication between device A and device B (⑦).

Authentication is indispensable in IoT systems, yet conventional PUF approaches incur significant overheads for storing CRPs^69^. Despite recent efforts to reduce requirements with single-CRP schemes^12^, they still face susceptibility to machine learning attacks^70^. Fortunately, the in-situ multidimensionally-encoded optical responses from our PUF provide resilience against machine learning attacks, facilitating simplified authentication protocols. We introduced a lightweight mutual authentication platform based on our all-Si multidimensionally-encoded optical PUFs, as depicted in Fig. 5. Two scenarios were explored: device-server and device-device mutual authentications. Prior to mutual authentication, the server retains the identity (ID) and the CRP (C_i_, R_i_) for each IoT device, while the IoT device retains no information, minimizing potential vulnerabilities. The mutual authentication protocol for the communication between an IoT device and a server is illustrated in Fig. 5 (blue lines) and proceeds as follows:

1. Initialization: The device starts the process by sending its unique ID and a random number (N_1_) to the server.
2. Server Validation: If the server recognizes the device ID, it generates a new random number (N_2_). It also constructs an encrypted message (M_A_) using a secret response (R_i_) stored for that device. This R_i_ is selected randomly from a set of five possible optical responses configured on the device. This multidimensionally-encoded response set substantially bolsters security compared to single-dimensional response PUFs. The server sends M_A_, a challenge (C_i_), and a message authentication code (MAC) to the device.
3. IoT Device Response: The device uses C_i_ to generate the matching response R_i_ and decrypts M_A_ using R_i_ to retrieve N_2_. It validates the MAC to check the integrity of the data from the server. If successful, the device generates a new CRP (C_i+1_, R_i+1_), random number (N_3_), encrypted message (M_S_), MAC, and sends these to the server.
4. Server Validation and Handshake: The server authenticates the MAC and uses the stored R_i_ to extract N_3_ and R_i+1_ from the encrypted M_S_. If successful, this completes mutual authentication—both sides have proven knowledge of the shared secret R_i_.

Additionally, the protocol for mutual authentication between devices adopts a similar methodology, employing the server as the conduit through which the authentication of the two devices to each other is facilitated. For further details, please refer to Supplementary Note 6. In summary, we presented a lightweight and secure mutual authentication platform based on all-Si multidimensionally-encoded optical PUF that offers excellent benefits for the IoT field.

## Discussion

In this work, we have demonstrated advanced all-Si multidimensionally-encoded optical PUFs fabricated with a CMOS-compatible process. Five in-situ optical responses encompassing random micropatterns (R_1_), the PL intensity of Si QDs (R_2_), the PL intensity of Er^3+^ (R_3_), the PL wavelength of Si QDs (R_4_), and the PL lifetime of Si QDs (R_5_) provide exceptionally high information entropy (up to 2.32 bits/pixel). These distinct optical responses arise from the varying coupling strengths between the Si metasurface and Er–Si QDs at different spatial positions. The excellent anti-counterfeiting performance of the PUFs, including bit uniformity, similarity, Intra-HD, Inter-HD, FAR, FRR, and encoding capacity, is thoroughly evaluated. In addition, the stability of the PUFs across extreme conditions such as heating, abrasion, UV radiation, and laser exposure has been demonstrated, validating their robustness for demanding applications. Based on the current PUFs, efficient lightweight mutual authentication protocols for IoT are proposed. The seamless integration of our PUFs with integrated circuits should open a path to the secure identification and authentication of devices in the IoT.

## Methods

### Materials

A premixed gas containing 20% argon (Ar) and 80% silane (SiH_4_) was acquired from Linde Electronic & Specialty Gases Co., Ltd. (Suzhou, China). Erbium(III) 2,2,6,6-tetramethyl-3,5-heptanedionate (Er(tmhd)_3_, 99.999%) was obtained from Nanjing Ai Mou Yuan Scientific equipment Co., Ltd. (Nanjing, China). Additional chemical reagents, including nitric acid (HNO_3_, 65–68%), hydrogen peroxide (H_2_O_2_, 30%), hydrofluoric acid (HF, 40%), copper nitrate (Cu(NO_3_)_2_.3H_2_O, 99%), methanol (98.5%), and toluene (99.5%), were acquired from Sinopharm Chemical Reagent Co., Ltd. (Shanghai, China). Mesitylene (97%) and 1-dodecene (95%) were sourced from Aladdin (Shanghai, China). PMMA (950 A4), featuring a 4.1% mass fraction in anisole, was purchased from Taizhou SUNANO New Energy Co., Ltd. (Taizhou, China). Heavily arsenic-doped Si wafers (〈100〉 orientation, <0.005 Ω cm resistivity) were obtained from Zhejiang Jinruihong Technology Co., Ltd. (Ningbo, China).

### Synthesis of Er–Si QDs

Er–Si QDs were fabricated by nonthermal plasma synthesis. A controlled gas mixture of 4.8 sccm SiH_4_/Ar (20% by volume) and 500 sccm Er(tmhd)_3_/Ar was fed into the plasma reactor. Er(tmhd)_3_, the erbium precursor, was vaporized in a heated bubbler at 160 °C, under a constant plasma pressure of 3.3 mbar. A 13.56 MHz power source and matching network were used to generate plasma with an approximate power of 60 W. After the synthesis, the as-produced Er–Si QDs were hydrosilylated with 1-dodecene.

### PUF fabrication

The substrates employed in this study were 〈100〉-oriented and heavily arsenic-doped Si (<0.005 Ω cm) slices with a surface area of 1.5 × 1.5 cm^2^. The substrates were cleaned with acetone and ethanol before etching. We etched the substrates at 50 °C for 1 min in a solution containing 3.28 g Cu(NO_3_)_2_·3H_2_O, 60.9 mL HF, and 58.1 mL H_2_O_2_ in a polytetrafluoroethylene container, producing random inverted pyramid arrays. After etching, the substrates were ultrasonically cleaned in HNO_3_ for 20 min, rinsed with deionized water, and dried under flowing nitrogen. We dropped the hydrosilylated Er–Si QDs on the etched substrates and heated them at 160 °C for 30 min. We deposited a PMMA layer on the substrates by spin coating (2000 rpm, 45 s) and heated them at 160 °C for 30 min.

### Characterization

PL emission spectra were acquired using an FLS1000 system (Edinburgh Instruments). Transient measurements utilized a pulsed 405 nm excitation laser at 100 Hz, with PL decay quantified via time-corrected single photon counting. PL mapping (40 × 40 μm^2^) was read via a Confocal Raman System (WITEC alpha 300 R) with a 405 nm laser. Optical microscopy (Olympus mx50) was used to observe the micropattern of the PUFs. The optical absorption spectra of the PMMA films were acquired using a UV–vis–NIR spectrometer (HITACHI U-4100). TEM imaging was conducted with a Talos F200X G2 microscope (Thermo Fisher) at 200 kV. The morphology of the PUFs was characterized by SEM (JEOL JSMIT800).

### Digitization of PUFs

To generate binary sequences from PUF images, we devised custom MATLAB codes using MATLAB R2017b for image processing and digital key extraction. The image processing procedure comprised the following steps:

(1) Before analysis, the phase information of the image in the frequency domain was extracted. The discrepancies in the image, arising from device vibrations and other factors, were corrected using the principle of phase correlation. Moreover, the speeded-up robust features (SURF) algorithm was employed to verify the positions of identical feature regions within the image^63^.

(2) Next, Gabor filtering (Supplementary Note 7) was applied to improve local feature extraction and noise reduction. Then, the image was transformed into the desired key size through binning operations. Finally, a global search algorithm^23^ was employed to iteratively find the most suitable threshold for image binarization.

(3) The HD between two data matrices was computed. The Inter-HD was determined by comparing the HD of different PUFs, while the Intra-HD was obtained by comparing the HD of the same PUFs measured independently.

(4) The similarity index served as a simple performance metric between the two PUFs. If a pixel-by-pixel comparison revealed discrepancies between the binary code arrays, the count of “0” outcomes was tallied. The percentage of “0” in relation to the total number of pixels represented the similarity measure.

### FDTD simulation

In this article, we conducted FDTD simulations using the software “Lumerical Solutions”. The Er–Si QDs were treated as electric dipole sources in this simulation, focusing on analyzing the Purcell factor and electric field distributions that include the reflected, exciting, and radiated fields. To accurately model the physical setup, we based our simulation on SEM images of the PUF surface. The simulation space was bounded by perfectly matched layer (PML) conditions in all three dimensions (x, y, and z) to minimize any reflection artifacts that could skew the results. For the reflected field analysis, we employed a total-field scattered-field (TFSF) source with a wavelength of 830 nm, representative of the emission characteristics of the dipole sources within the Er–Si QDs. Similarly, to simulate the exciting field, another 405 nm TFSF source was used to mimic the excitation conditions of the Er–Si QDs. Our simulation domain included a Si metasurface characterized by a random distribution, onto which we placed nine dipole sources at varying locations to simulate the distribution and emission of light from the Er–Si QDs. A highly detailed grid layout with 1 nm × 1 nm × 1 nm resolution was chosen to capture the intricate electric field variations near the metasurface and dipole sources. To extract the simulation results, we utilized electric field monitors and Purcell factor monitors strategically placed within the domain. These tools enabled us to capture the detailed electric field distributions (reflecting the reflected, excited, and radiated electric fields) and the Purcell factor enhancement at specific points on the Si metasurface.

### Supplementary information

Supplementary Information

Peer Review File

### Source data

Source Data

## Data Availability

The authors confirm that data supporting the findings of this study are available within the article and its Supplementary Information. Additional data supporting these findings are available from the corresponding author upon reasonable request. Source data are provided in this paper.

## Code availability

The MATLAB (R2017b) codes employed for data processing and digitization are included with the source data in the Source Data file.
