# Supplementary material for: All-silicon multidimensionally-encoded optical physical unclonable functions for integrated circuit anti-counterfeiting
Source: Nat Commun. 2024 Apr 13;15:3203. doi: 10.1038/s41467-024-47479-y (PMC11016093; doi:10.1038/s41467-024-47479-y)
Supplement: Supplementary file 1 — Supplementary Information [file 41467_2024_47479_MOESM1_ESM.pdf]

Supplementary Information

All-silicon multidimensionally-encoded optical physical unclonable functions for integrated circuit anti-counterfeiting

Kun Wang[1], Jianwei Shi[2,3], Wenxuan Lai[1], Qiang He[1], Jun Xu[4], Zhenyi Ni[1*], Xinfeng Liu[2*], Xiaodong Pi[1,5*] & Deren Yang[1,5*]

[1]State Key Laboratory of Silicon and Advanced Semiconductor Materials & School of Materials Science and Engineering, Zhejiang University, Hangzhou, Zhejiang 310027, China

[2]CAS Key Laboratory of Standardization and Measurement for Nanotechnology, National Center for Nanoscience and Technology, Beijing 100190, China

[3]State Key Laboratory for Superlattices and Microstructures, Institute of Semiconductors, Chinese Academy of Sciences, Beijing 100083, China

[4]School of Electronic Science and Engineering & National Laboratory of Solid State Microstructures, Nanjing University, Nanjing, Jiangsu 210093; School of Microelectronics, Nantong University, Nantong, Jiangsu 226019, China

[5]Institute of Advanced Semiconductors, ZJU-Hangzhou Global Scientific and Technological Innovation Centre, Zhejiang University, Hangzhou, Zhejiang 311215, China

*E-mail: zyni@zju.edu.cn; liuxf@nanoctr.cn; xdpi@zju.edu.cn; mseyang@zju.edu.cn

Supplementary Table 1. The PUF fabrication process and estimation of detailed cost for fabricating one PUF with the size of 1.5 × 1.5 cm^2.

Fabrication process	CMOS compatibility	Material/equipment		Unit price/ depreciation cost	Consumption	Cost (USD)	[a]The number of suppliers	Remarks
①Fabrication of Er-Si QDs toluene solution.	Compatible with plasma vapor deposition[1,2] and wet chemistry[3] in CMOS processes.	Material	SiH_4	14.88 USD/L	0.003 L	0.045	>100	Given that the fabrication of a single PUF with the size of 1.5 × 1.5 cm^2 necessitates 50 µL of Er-Si QDs in toluene solution, the consumption of materials and the depreciation cost of equipment are correspondingly proportional to the amounts and expenses required to prepare 50 µL of Er-Si QDs toluene solution. The depreciation cost of equipment is calculated over a 10-year period, with a residual value rate of 5%. The formula for calculating equipment depreciation cost is as follows: Equipment Depreciation Cost = [Cost of Equipment × (1 - 5%)] / (10 × 365 × 24 hours) × Actual Usage Time (hours). The same
			Ar	0.77 USD/L	0.3 L	0.23	>1000	
			$Er(tmhd)_3$	21 USD/g	0.0003 g	0.0063	>100	
			Glass tubes	0.42 USD/piece	1 piece	0.42	>6000	
			Methanol	4.39 USD/L	0.00005 L	0.00022	>100	
			HF	6.12 USD/L	0.000005 L	0.00003	>100	
			1-Dodecene	43.43 USD/L	0.00001 L	0.00043	>100	
			Mesitylene	21.47 USD/L	0.0001 L	0.0021	>100	
			Toluene	7.18 USD/L	0.00005 L	0.00036	>100	
		Equipment	$Er(tmhd)_3$ bottle	0.0018 USD/g	1 hour	0.0018	>6000	
			$Er(tmhd)_3$ Heater	0.00057 USD/hour	1 hour	0.00057	>6000	
			Vacuum Gauge	0.00046 USD/hour	1 hour	0.00046	>6000	
			Vacuum Pumps	0.00069 USD/hour	1 hour	0.00069	>6000	
			Pipeline Heaters	0.00036 USD/hour	1 hour	0.00036	>6000	
			Steel Tubing	0.000063 USD/hour	1 hour	0.000063	>6000	
			RF Power Supply	0.00046 USD/hour	1 hour	0.00046	>100	
			Matching box	0.000231 USD/hour	1 hour	0.000231	>100	

			Heating jacket	0.0000015 USD/hour	1 hour	0.0000015	>6000	method is applied for calculating the depreciation costs of subsequent equipment.<br><br>One person can prepare 10 mL of Er-Si QDs in toluene solution within 3 hours, then the actual time required to prepare 50 µL of the Er-Si QDs in toluene solution is 0.015 hours. The average power of our equipment during the fabrication process is 2 kW
			Rotary evaporator	0.0000076 USD/hour	1 hour	0.0000076	>6000	
		Electricity	-	0.11 USD/kWh	0.03 kWh	0.0033	-	
		Labor	-	2.8 USD/hour	0.015 hours	0.042	-	
②<br>Preparation of silicon metasurface	Compatible with wet chemical etching[3–5] in CMOS processes.	Material	Silicon wafer	0.07 USD/cm^2	2.25 cm^2	0.16	>100	Given that a single batch allows for the preparation of 60 pieces of PUFs with the size of 1.5 × 1.5 cm^2, the consumption of materials and the depreciation cost of equipment are respectively equivalent to the amounts and expenses required to fabricate a single PUF with the size of 1.5 × 1.5 cm^2.
			Acetone	6.38 USD/L	0.0017 L	0.011	>100	
			Ethanol	4.39 USD/L	0.0017 L	0.0075	>100	
			$Cu(NO_3)_2.3H_2O$	0.022 USD/L	0.055 L	0.0012	>100	
			HF	6.11 USD/L	0.0012 L	0.0073	>100	
			H_2O_2	6.91 USD/L	0.00096 L	0.0066	>100	
			HNO_3	6.91 USD/L	0.0017 L	0.012	>100	
			Deionized water	1.12 USD/L	0.0017 L	0.0019	>6000	
			N_2	0.24 USD/L	0.00083 L	0.0002	>100	

		Equipment	Ultrasonic machine	0.0000051 USD/hour	1 hour	0.0000051	>6000	One person is capable of preparing 60 PUFs within one hour, then the actual time required to prepare a single PUF is ~0.017 hours. The average power of our equipment during the fabrication process is 1 kW
			Heating table	0.0000051 USD/hour	1 hour	0.0000051	>6000	
			Nitrogen Gun	0.0000026 USD/hour	1 hour	0.0000026	>6000	
		Electricity	-	0.11 USD/kWh	0.017 kWh	0.0019	-	
		Labor	-	2.8 USD/hour	0.017 hour	0.048	-	
③ Encapsulation	Compatible with plastic encapsulation[3,5,6] in CMOS processes.	Material	PMMA	3080 USD/L	0.00005 L	0.15	>100	One person is capable of encapsulating 10 PUFs within one hour, then the actual time required to encapsulate a single PUF is 0.1 hours. The average power of our equipment during the fabrication process is 1 kW
		Equipment	Pipette Gun	0.00003 USD/hour	1 hour	0.00003	>6000	
			Spin Coater	0.00045 USD/hour	1 hour	0.00045	>6000	
			Heating Station	0.00003 USD/hour	1 hour	0.00003	>6000	
		Electricity	-	0.11 USD/kWh	0.1kWh	0.011	-	
		Labor	-	2.8 USD/hour	0.1 hour	0.28	-	
Total	-	-	-	-	-	1.45	-	In the future, through measures such as batch preparation, process optimization, and automation, there is potential for further reduction in costs.

[a] The number of suppliers is totaled from Google, Alibaba, and Amazon.

Supplementary Note 1 | The cost of each PUF label.

The total cost for fabricating one piece of 1.5 × 1.5 cm^2 sized PUF label is 1.45 USD. Due to the high information entropy of our PUFs, a tiny working area can meet the purpose of anti-counterfeiting. In our study, the area used for encoding is 40 μm × 40 μm. Thus, a 1.5 × 1.5 cm^2 sized PUF label can be cut into a maximum of ~1.4×10^5 pieces of working labels, and the cost of an individual working label will be significantly low (~ 10^{-5} USD). In addition, there is potential for further reduction in costs through measures such as batch preparation, process optimization, and automation.

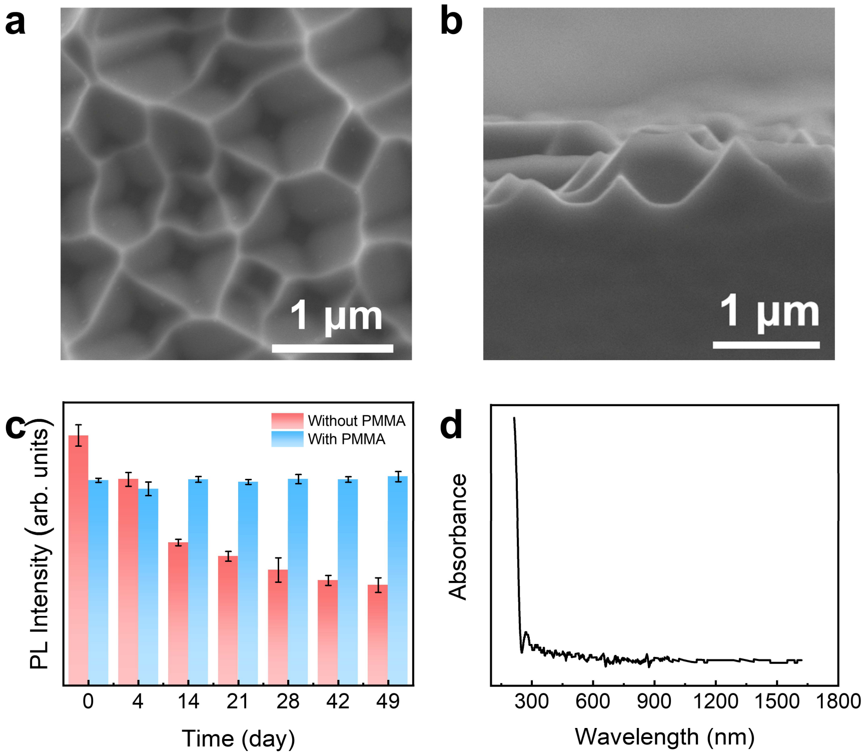

Supplementary Fig. 1 | a SEM image at the top view of the random inverted pyramid arrays. **b** SEM image at the side view of the random inverted pyramid arrays. **c** The Si QD-related PL intensity of the same area on an all-Si multidimensionally-encoded optical PUF, with and without PMMA encapsulation, after immersion in deionized water for varying durations. The error bars represent the standard deviation from five independent measurements. **d**. Absorption spectrum of PMMA.

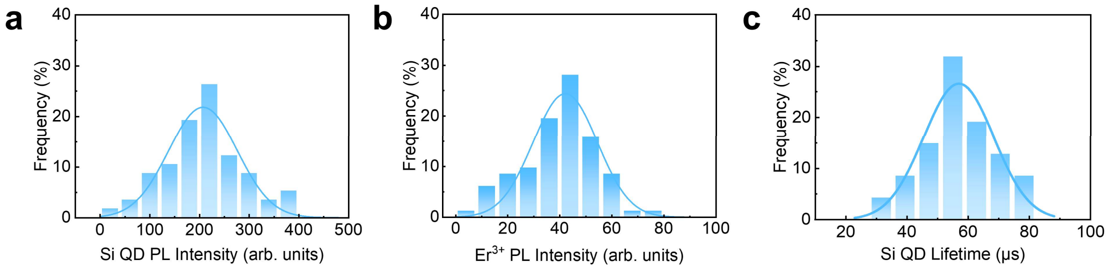

Supplementary Fig. 2 | Statistical distribution of integrated intensity of Si QD PL intensity (**a**), Er^{3+} PL intensity (**b**), and Si QD PL lifetime (**c**).

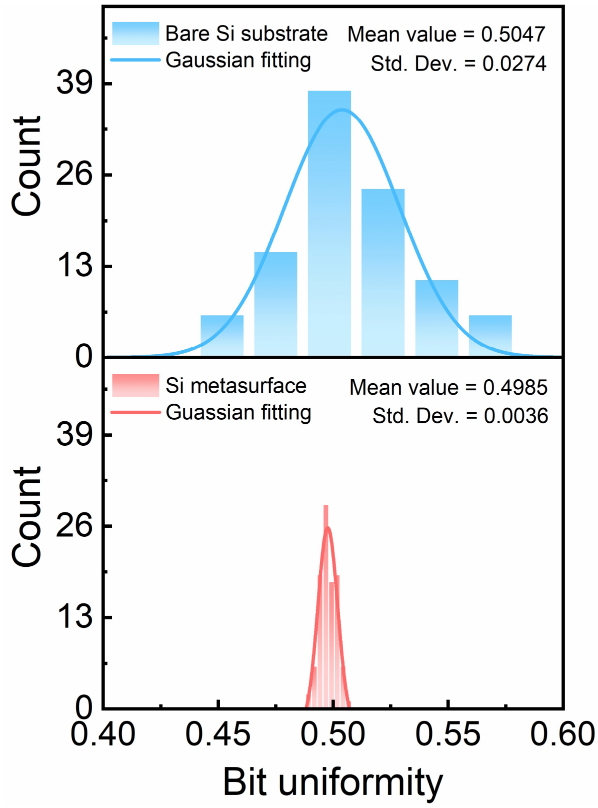

Supplementary Fig. 3 | Comparison of bit uniformity (Supplementary Formula 5) distribution for Er^{3+} PL intensity (R_3) derived from Si metasurface (bottom) and bare Si substrate (top). Each distribution is based on analyses of 100 images.

Supplementary Note 2 | Information entropy calculation.

In the domain of information theory, the assumption of a discrete stochastic variable, X, possessing an assortment of feasible results such as x_1, x_2,…, x_n, which materialize with respective probabilities $P(x_1)$, $P(x_2)$,…,$P(x_n)$, culminates in the delineation of the entropy of X. The information entropy of X is defined as[7]

$$\text{Information entropy} = -\sum_{i=1}^{n} P(x_i)\log_2[P(x_i)] \qquad (1)$$

For our all-Si multidimensionally-encoded optical PUFs, multiple optical responses have an equal occurrence probability of 1/5; therefore, the information entropy is calculated as 2.32 bits/pixel.

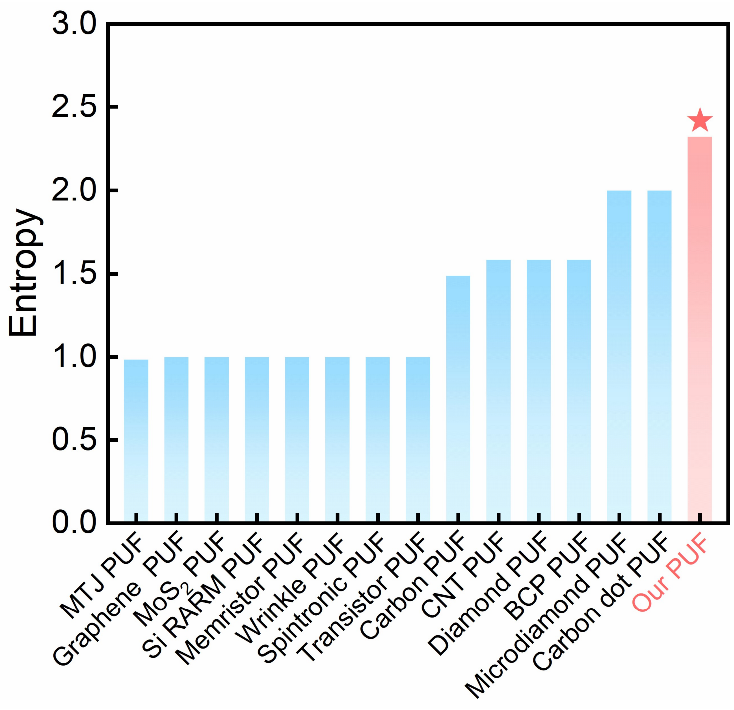

Supplementary Fig. 4 | A critical comparison of the information entropy of diverse PUFs, including MTJ PUF[8], graphene PUF[9], MoS_2 PUF[10], Si RARM PUF[11], memristor PUF[12], wrinkle PUF[13], spintronic PUF[14], transistor PUF[15], carbon PUF[16], carbon nanotube (CNT) PUF[17], diamond PUF[18], block co-polymer (BCP) PUF[19], microdiamond PUF[20], carbon dot PUF[21], and our PUF.

Supplementary Note 3 | The generation of barcodes or QR codes based on Si QD-related PL wavelength (R_4),

Threshold value: Average wavelength (λ) of the all-Si multidimensionally-encoded optical PUFs

λ < threshold = 0, λ > threshold = 1. Barcode creation by code 128.

all-Si multidimensionally-encoded optical PUF#1

**Position**	**1**	**2**	**3**	**4**	**5**	**6**	**7**	**8**	**9**	**10**
λ (nm)	788	811	839	858	820	801	843	815	846	844
**Position**	**11**	**12**	**13**	**14**	**15**	**16**	**17**	**18**	**19**	**20**
λ (nm)	837	844	817	798	810	797	813	797	804	835
Threshold (nm)	820.85									

String	1	2	3	4	5	6	7	8	9	10	11	12	13	14	15	16	17	18	19	20
Digit	0	0	1	1	0	0	1	0	1	1	1	1	0	0	0	0	0	0	0	1

all-Si multidimensionally-encoded optical PUF#2

**Position**	**1**	**2**	**3**	**4**	**5**	**6**	**7**	**8**	**9**	**10**
λ (nm)	803	796	813	835	812	803	813	800	805	816
**Position**	**11**	**12**	**13**	**14**	**15**	**16**	**17**	**18**	**19**	**20**
λ (nm)	809	812	848	843	838	813	800	797	821	848
Threshold (nm)	816.25									

String	1	2	3	4	5	6	7	8	9	10	11	12	13	14	15	16	17	18	19	20
Digit	0	0	0	1	0	0	0	0	0	0	0	0	1	1	1	0	0	0	1	1

all-Si multidimensionally-encoded optical PUF#3

**Position**	**1**	**2**	**3**	**4**	**5**	**6**	**7**	**8**	**9**	**10**
λ (nm)	840	799	784	866	864	850	803	801	866	874
**Position**	**11**	**12**	**13**	**14**	**15**	**16**	**17**	**18**	**19**	**20**
λ (nm)	784	773	868	869	798	802	889	843	792	781
Threshold (nm)	827.3									

String	1	2	3	4	5	6	7	8	9	10	11	12	13	14	15	16	17	18	19	20
Digit	1	0	0	1	1	1	0	0	1	1	0	0	1	1	0	0	1	1	0	0

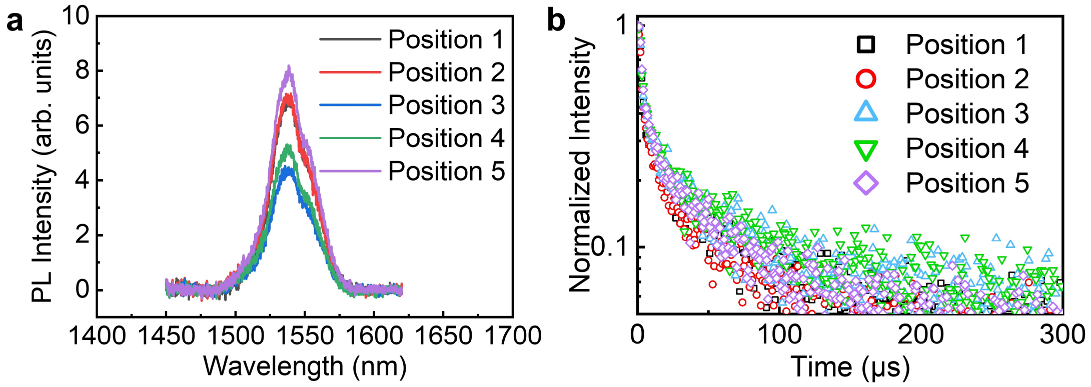

Supplementary Fig. 5 | a Er^{3+} PL spectra of the PUF on different positions. **b** The PL lifetime of Er^{3+} at different positions.

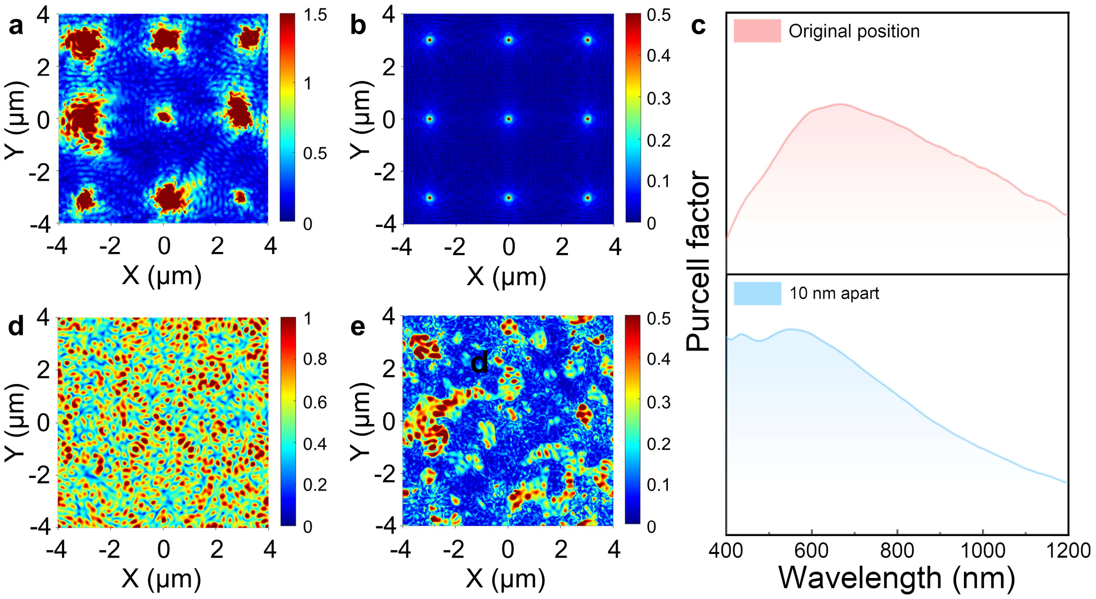

Supplementary Fig. 6 | Field distributions of nine distinct dipoles at the surfaces of the Si metasurface (**a**) and non-patterned Si (**b**). **c** Purcell factors of two dipoles situated 10 nm apart on the Si metasurface. **d** Reflected field 5 μm above the PUF surface for an 830 nm wavelength plane wave. **e** Exciting field on the Si metasurface surface at 405 nm wavelength.

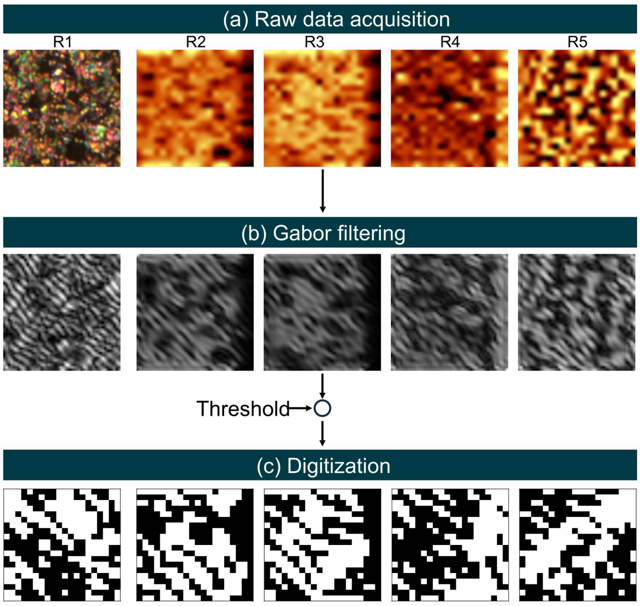

Supplementary Fig. 7 | Description of the bit extraction process, involving: (a) raw data acquisition, (b) Gabor filtering, and (c) digitization.

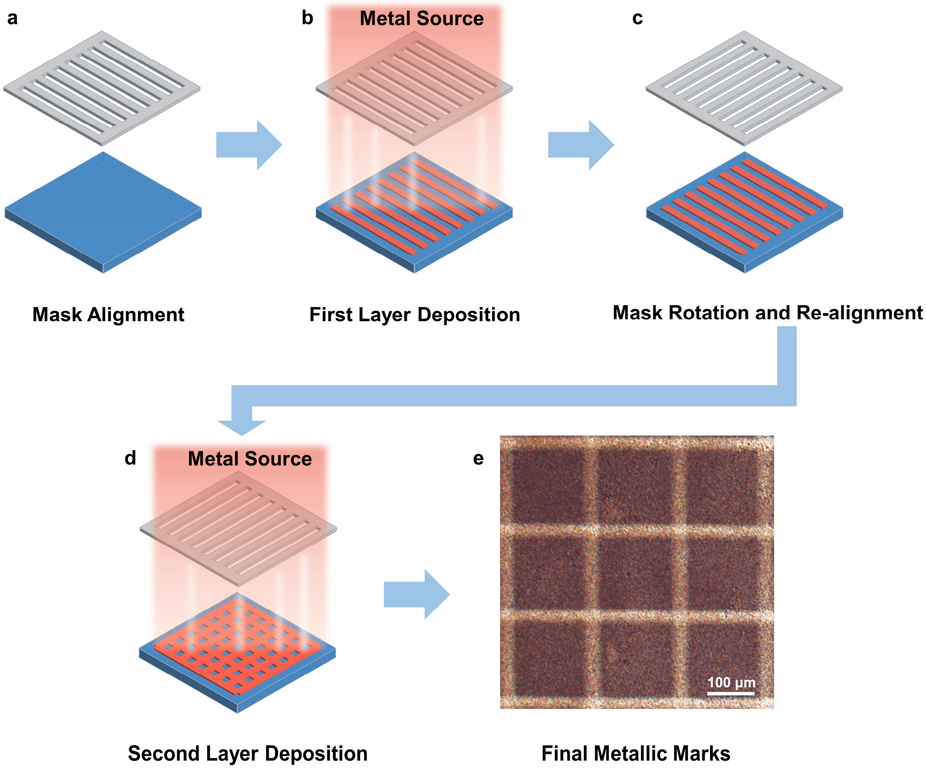

Supplementary Fig. 8 | Schematic diagram of the production process of metallic marks. a Mask alignment. **b** First layer deposition. **c** Mask rotation and re-positioning. The mask is rotated 90° clockwise. **d** Second layer deposition. **e** Final metallic marks.

Supplementary Note 4 | The principle of phase correlation.

Before analyzing, it is imperative to extract the phase information of the image in the frequency domain. By utilizing the principle of phase correlation, the discrepancies in the image caused by factors such as device instability are rectified. Taking the initial image as a fundamental reference, a Fourier transformation is then applied to it:

$$F(u,v) = FFT(f(x,y)) = \sum_{x,y} f(x,y) \exp\left[-2\pi i\left(\frac{ux}{M} + \frac{vy}{N}\right)\right] \quad (2)$$

where x and y denote the coordinates of the image, while u and v represent the coordinates in the frequency spectrum. M and N respectively signify the dimensions of the image in the two directions. Subsequently, if the captured image deviates due to device instability, the following relationship will emerge in comparison to the reference image:

$$f(x,y) = g(x + dx, y + dy) \quad (3)$$

where dx and dy represent the amount of displacement caused by device instability in the x and y directions respectively, this deviation manifests in the frequency domain as the following displayed phase difference:

$$F(u,v) = G(u,v) \exp\left[2\pi i\left(\frac{udx}{M} + \frac{vdy}{N}\right)\right] \quad (4)$$

where G(u,v) signifies the frequency spectrum matrix corresponding to the image of the deviation, and $\exp\left[2\pi i\left(\frac{udx}{M} + \frac{vdy}{N}\right)\right]$ represents the correction factor. Once the correction is accomplished, the speeded-up robust features (SURF) algorithm[22] is employed to verify the location of identical feature regions within the image.

Supplementary Note 5 | Calculation of various performances of all-Si multidimensionally-encoded optical PUF

$$\text{Bit uniformity} = \frac{1}{s}\sum_{l=1}^{s} K_l \quad (5)$$

where K_l is the *l*th bit response (0 or 1) of an array with *s* bits.

$$\text{Similarity} = \frac{\boldsymbol{C_0}}{\boldsymbol{C}} \quad (6)$$

where $\boldsymbol{C}$ is the matrix difference between two PUF images after binary digitalization.

The digitized matrices of two PUF images can be represented as: $\mathbf{A} = \begin{bmatrix} a_{11} & \cdots & a_{1n} \\ \vdots & \ddots & \vdots \\ a_{m1} & \cdots & a_{mn} \end{bmatrix}$, $\mathbf{B} = \begin{bmatrix} b_{11} & \cdots & b_{1n} \\ \vdots & \ddots & \vdots \\ b_{m1} & \cdots & b_{mn} \end{bmatrix}$, $\mathbf{C} = \mathbf{A} - \mathbf{B} = \begin{bmatrix} a_{11} - b_{11} & \cdots & a_{1n} - b_{1n} \\ \vdots & \ddots & \vdots \\ a_{m1} - b_{m1} & \cdots & a_{mn} - b_{mn} \end{bmatrix}$. $\boldsymbol{C_0}$ is the number of "0" in matrix $\boldsymbol{C}$.

$$\text{Intra} - \text{Hamming distance} = \frac{1}{m}\sum_{t=1}^{m}\frac{\text{HD}(K_i, K_{i,t})}{s} \tag{7}$$

where K_i and $K_{i,t}$ are the original s-bit reference key and a s-bit key extracted from the same PUF device at a different time-point t and m is the number of repeated measurements.

$$\text{Inter} - \text{Hamming distance} = \frac{2}{q(q-1)}\sum_{i=1}^{q-1}\sum_{j=i+1}^{q}\frac{\text{HD}(K_i, K_j)}{s} \tag{8}$$

where K_i and K_j are s-bit keys of the ith PUF device and the jth PUF device among q different PUFs, respectively.

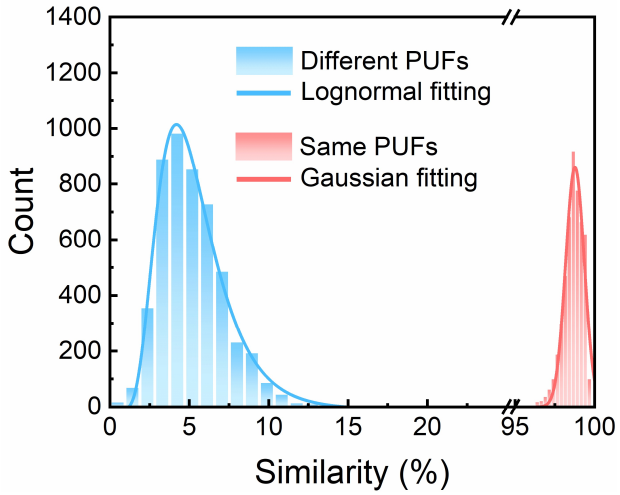

Supplementary Fig. 9 | Distribution of the similarity for the different versus same PUFs in terms of per pixel binary encoding.

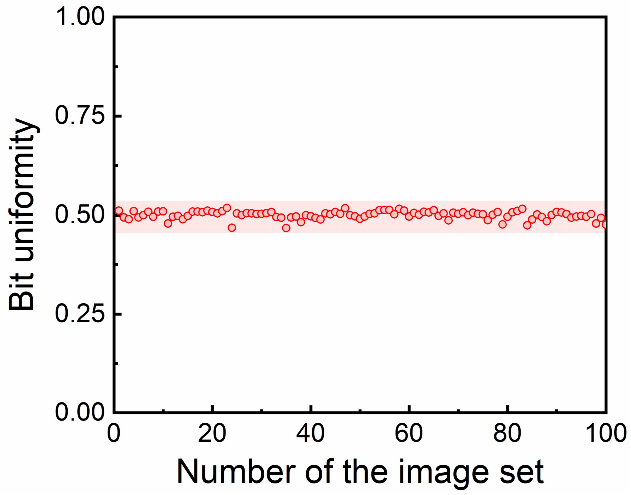

Supplementary Fig. 10 | The normalized Hamming distance (HD) across the digitized keys of the five responses.

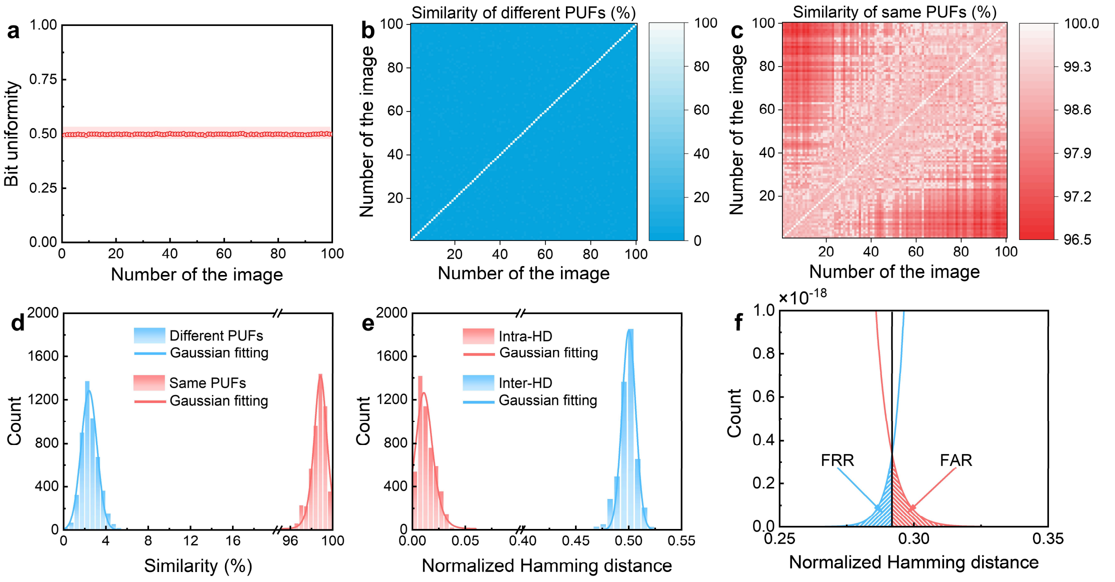

Supplementary Fig. 11 | Performance of the all-Si multidimensionally-encoded optical PUF based on the optical response of random micropattern(R_1). **a** Occurrence probability of "1" in binary bits extracted from 30 images. **b** Pairwise match of 30 different PUFs using per pixel binary encoding of images acquired from the different positions. **c** Pairwise match of 30 same PUFs using per pixel binary encoding of images acquired from the same position. **d** Distribution of the similarity for the different versus same PUFs in terms of per pixel binary encoding. **e** Distribution of normalized Hamming distance (HD) for Inter-HD and intra-HD. **f** Magnified Inter-HD and Intra-HD.

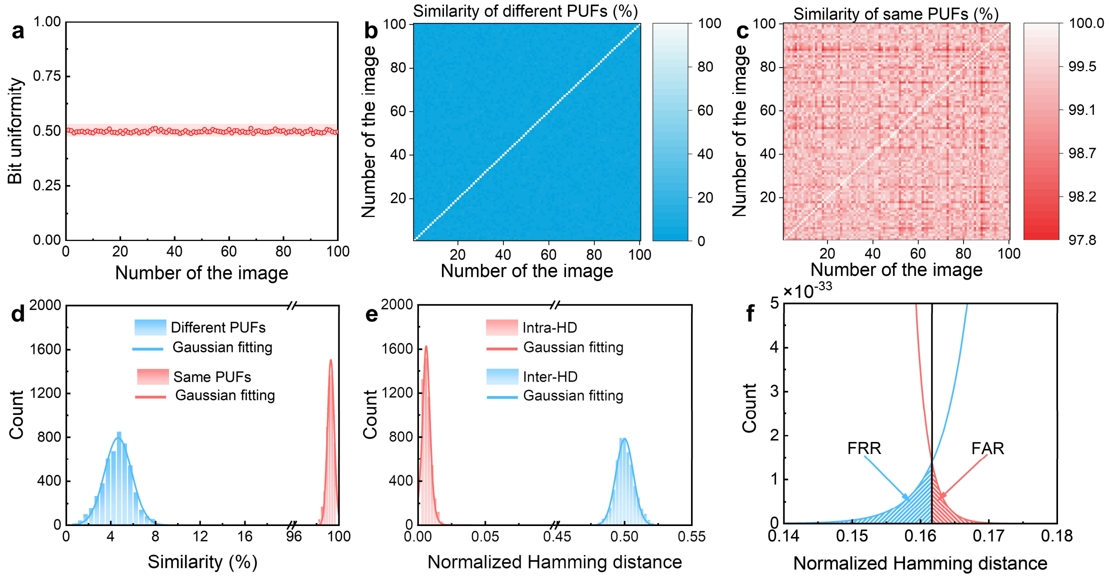

Supplementary Fig. 12 | Performance of the all-Si multidimensionally-encoded optical PUF based on the optical response of Si QD-related PL intensity (R_2). **a** Occurrence probability of "1" in binary bits extracted from 30 images. **b** Pairwise match of 30 different PUFs using per pixel binary encoding of images acquired from the different positions. **c** Pairwise match of 30 same PUFs using per pixel binary encoding of images acquired from the same position. **d** Distribution of the similarity for the different versus same PUFs in terms of per pixel binary encoding. **e** Distribution of normalized Hamming distance (HD) for Inter-HD and intra-HD. **f** Magnified Inter-HD and Intra-HD.

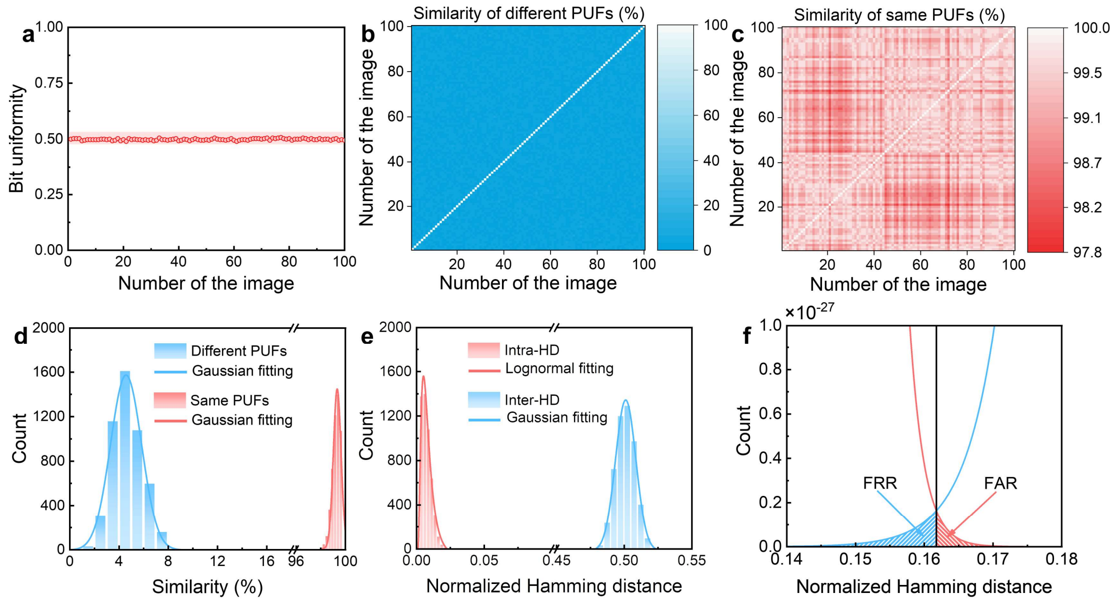

Supplementary Fig. 13 | Performance of the all-Si multidimensionally-encoded optical PUF based on the optical response of Er^{3+}-related emission intensity (R_3). **a** Occurrence probability of "1" in binary bits extracted from 30 images. **b** Pairwise match of 30 different PUFs using per pixel binary encoding of images acquired from the different positions. **c** Pairwise match of 30 same PUFs using per pixel binary encoding of images acquired from the same position. **d** Distribution of the similarity for the different versus same PUFs in terms of per pixel binary encoding. **e** Distribution of normalized Hamming distance (HD) for Inter-HD and intra-HD. **f** Magnified Inter-HD and Intra-HD.

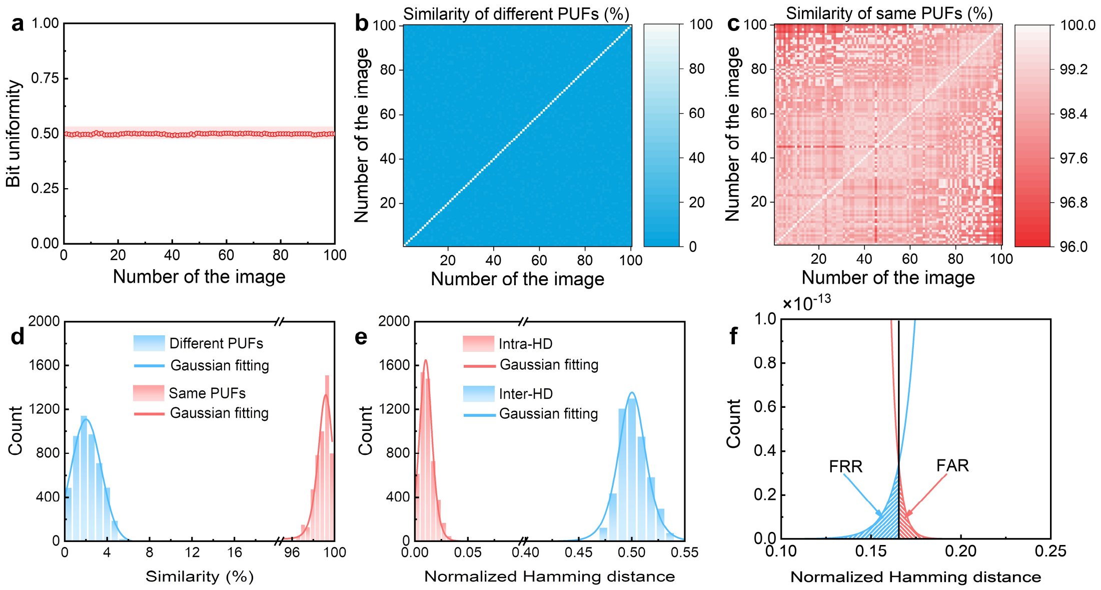

Supplementary Fig. 14 | Performance of the all-Si multidimensionally-encoded optical PUF based on the optical response of the Si QD-related PL lifetime (R_5). **a** Occurrence probability of "1" in binary bits extracted from 30 images. **b** Pairwise match of 30 different PUFs using per pixel binary encoding of images acquired from the different positions. **c** Pairwise match of 30 same PUFs using per pixel binary encoding of images acquired from the same position. **d** Distribution of the similarity for the different versus same PUFs in terms of per pixel binary encoding. **e** Distribution of normalized Hamming distance (HD) for Inter-HD and intra-HD. **f** Magnified Inter-HD and Intra-HD.

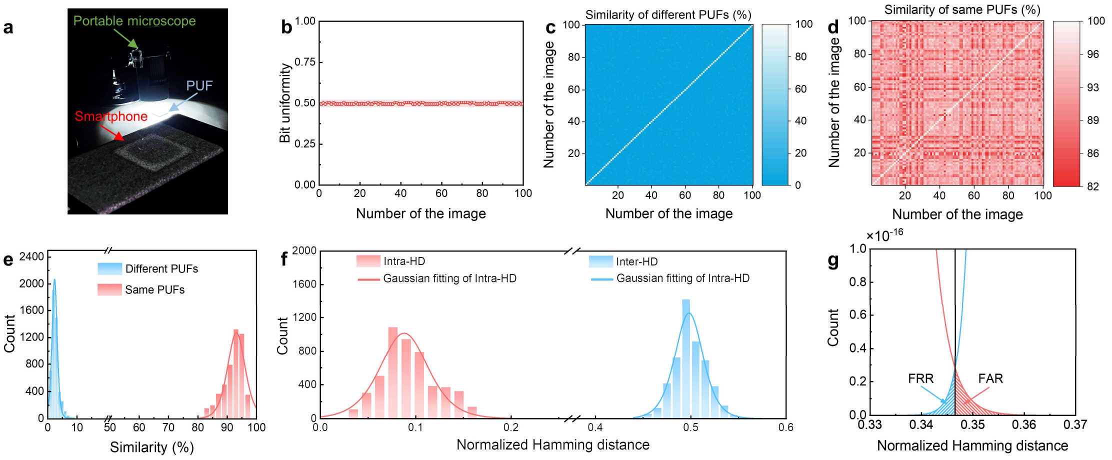

Supplementary Fig. 15 | a Experimental setup for capturing R_1 with a smartphone equipped with a portable microscope. **b** Occurrence probability of "1" in binary bits extracted from 100 images. **c** Pairwise match of 100 different PUFs using per pixel binary encoding of images acquired from the different positions. **d** Pairwise match of 100 same PUFs using per pixel binary encoding of images acquired from the same position. **e** Distribution of the similarity for the different versus same PUFs in terms of per pixel binary encoding. **f** Distribution of normalized Hamming distance (HD) for Inter-HD and intra-HD. **g** Magnified Inter-HD and Intra-HD.

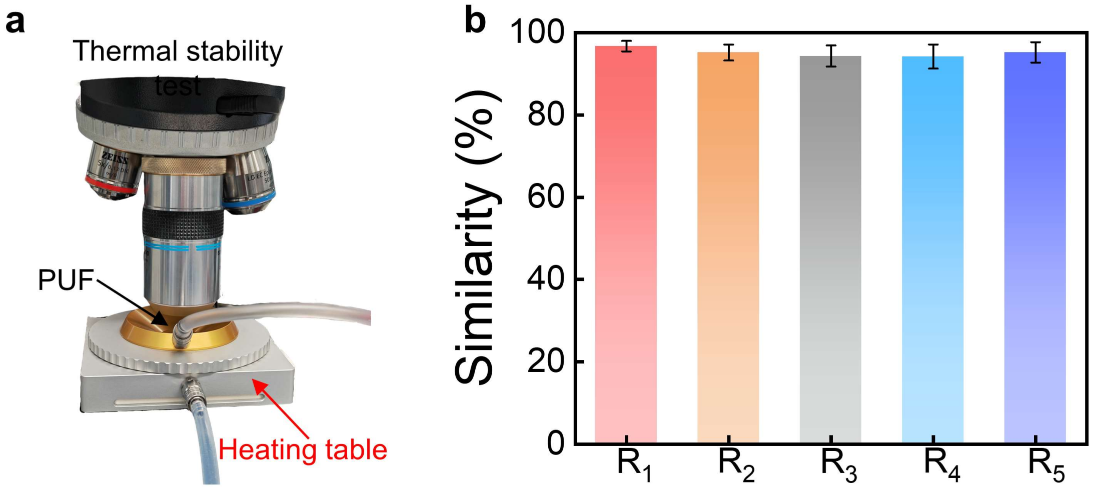

Supplementary Fig. 16 | Thermal stability measurement of the PUF devices. a Photograph of measurement setup for thermal stability test. **b** Average similarity of each optical response. Five PUF devices were tested for each optical response. The error bars represent the standard deviation from five independent measurements.

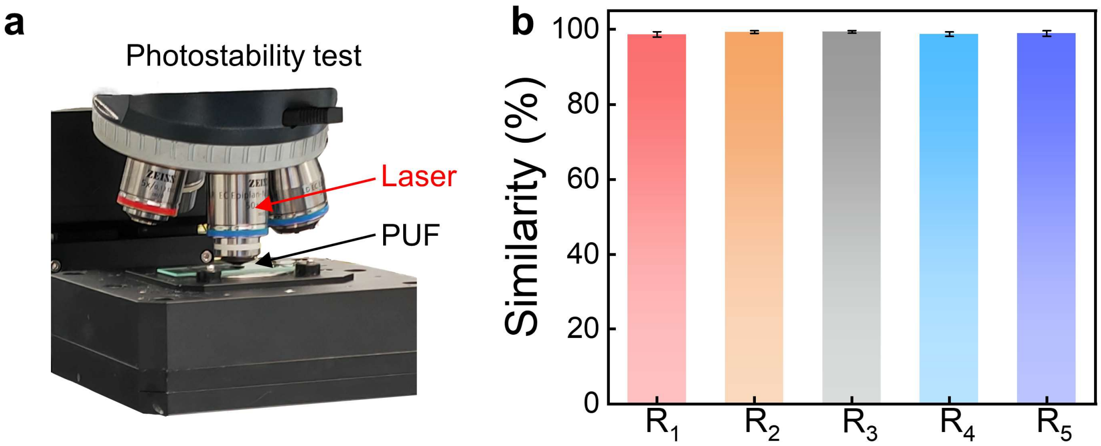

Supplementary Fig. 17 | Photostability measurement of the PUF devices. a Photograph of measurement setup for photostability test. **b** Average similarity of each optical response. Each optical response was tested 100 times. The error bars represent the standard deviation from 100 independent measurements.

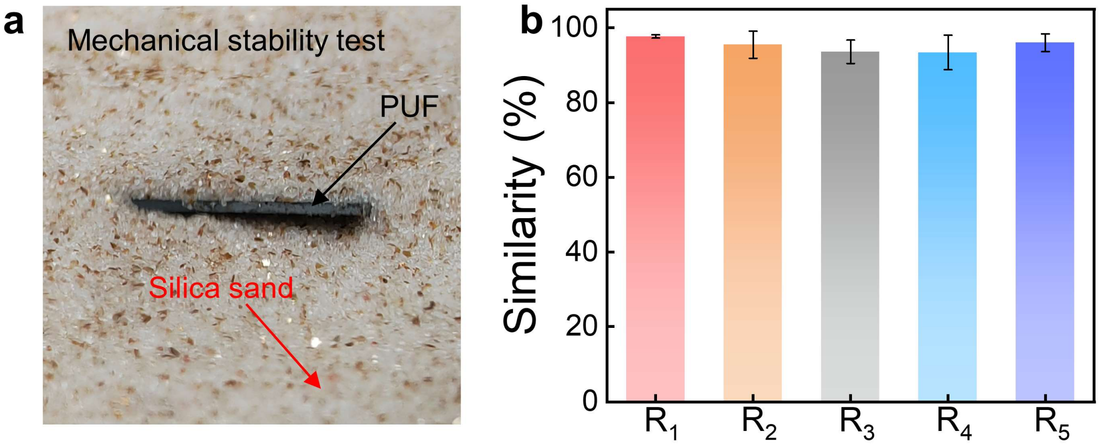

Supplementary Fig. 18 | Mechanical stability measurement of the PUF devices. a Photograph of measurement setup for mechanical stability test. **b** Average similarity of each optical response. Five PUF devices were tested for each optical response. The error bars represent the standard deviation from five independent measurements.

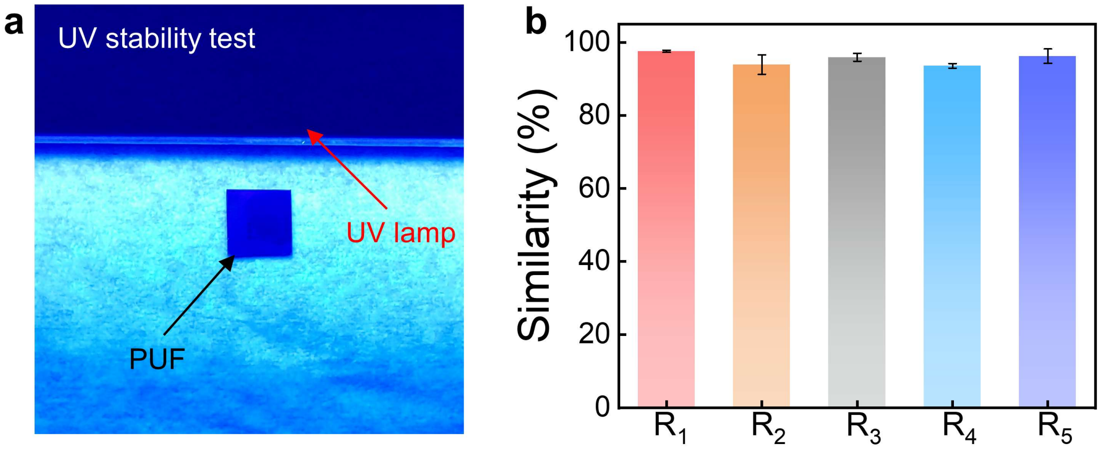

Supplementary Fig. 19 | UV stability measurement of the PUF devices. a Photograph of measurement setup for UV stability test. **b** Average similarity of each optical response. Five PUF devices were tested for each optical response. The error bars represent the standard deviation from five independent measurements.

Supplementary Note 6 | Mutual authentication protocol 2

In this note, we present the protocol for mutual authentication and key exchange. Consider a scenario where two IoT devices, ID_A and ID_B, intend to initiate a session and require mutual authentication. The secure establishment of trust between these devices is crucial to ensure the integrity and confidentiality of their communications. To achieve this, we propose an illustrated mutual authentication protocol (Fig. 5 red lines) that consists of the following steps:

① Initialization: In the authentication process, IoT device A initiates communication by transmitting its unique identifier (ID_A) and a randomly generated number (N_1) to IoT device B, establishing the initial connection.

② Initialization Relay: IoT device B acts as a relay, forwarding the identifiers (ID_A and ID_B) and random numbers (N_1 and N_2) to the server, facilitating the exchange of information between the IoT devices and the central server.

③ Server Validation: The server retrieves the CRPs associated with ID_A and ID_B, represented by (C_i, R_i) and (C_j, R_j) respectively. Utilizing these protocols, the server generates random numbers (R_{S1} and R_{S2}) and constructs encrypted messages (M_A and M_B) employing R_i and R_j. These encrypted messages,

along with their corresponding challenge signals (C_i and C_j) and message authentication codes (MACs), are then transmitted to IoT device A.

④ IoT Device A Response: IoT device A employs the challenge signal C_i to obtain the corresponding optical response signal R_i from the all-Si multidimensionally-encoded optical PUF. Subsequently, it undertakes the following tasks: (i) Decrypts the received messages using R_i to obtain R_{S1}, R_{S2}, and M_B. (ii) Verifies the integrity of the MAC. In the event of verification failure, IoT device A terminates the ongoing authentication attempt. (iii) Generates a random number N_A and derives a new optical response signal, R_{i+1}, from all-Si multidimensionally-encoded optical PUF. IoT device A then transmits the encrypted message M_{S1}, along with the corresponding MAC, to the server. The server employs R_i to derive N_A and R_{i+1}, subsequently verifying the integrity of the MAC. If the verification fails, the server rejects the proposed cryptographic key pair (CRP) update. Otherwise, the server updates the CRP associated with IoT device A.

⑤ Challenge Relay: IoT device A relays the challenge signal C_j, the encrypted messages (M_{B1}), and the corresponding MAC received from the server to IoT device B.

⑥ IoT Device B Response: IoT device B decrypts the received message, verifies the MAC integrity, generates a new random number (N_B), and updates its CRP accordingly. Subsequently, IoT device B dispatches a confirmation message to IoT device A, solidifying the authentication process.

⑦ Validation and Handshake: IoT device A verifies the integrity of the MAC associated with the confirmation message received from IoT device B. Upon successful verification, mutual authentication between IoT device A and IoT device B is achieved, confirming the legitimacy and trustworthiness of the devices.

Supplementary Note 7 | Gabor filtering

We fine-tuned the Gabor filtering parameters to improve local feature extraction and noise reduction, applying the formula[23]:

$$g_{\lambda,\theta,\varphi,\sigma,\gamma}(x,y) = e^{-\frac{x'^2+\gamma^2 y'^2}{2\sigma^2}} \cos\left(2\pi\frac{x'}{\lambda}\right) \tag{9}$$

with $x' = x\cos\theta + y\sin\theta$ and $y' = -x\sin\theta + y\cos\theta$, where x, y denote pixel

coordinates; λ is the wavelength of the filter; θ is the orientation of the Gabor kernel; σ is the standard deviation of the Gaussian envelope; γ is the spatial aspect ratio. The optimized parameters for our Gabor filtering were：$\lambda = 3$, $\theta = 45°$, $\sigma = 1$, $\gamma = 1$.

Supplementary References

1. Chia, X. X. & Tan, D. T. H. Deuterated SiN_x: A low-loss, back-end CMOS-compatible platform for nonlinear integrated optics. *Nanophotonics* **12**, 1613–1631 (2023).
2. Marchack, N. *et al.* Plasma processing for advanced microelectronics beyond CMOS. *J. Appl. Phys.* **130**, 080901 (2021).
3. Quirk, M. & Serda, J. *Semiconductor manufacturing technology* (Prentice Hall, New Jersey, 2001).
4. Liao, Y., Shin, S. H., Jin, Y., Wang, Q. J. & Kim, M. Producing microscale Ge textures via titanium nitride- and nickel-assisted chemical etching with CMOS-compatibility. *Adv. Mater. Interfaces* **8**, 2100937 (2021).
5. Cui, J. *et al.* CMOS-compatible electrochemical synaptic transistor arrays for deep learning accelerators. *Nat. Electron.* **6**, 292–300 (2023).
6. He, Z. *et al.* CMOS compatible high-performance nanolasing based on perovskite–SiN hybrid integration. *Adv. Opt. Mater.* **8**, 2000453 (2020).
7. Shannon, C. E. A mathematical theory of communication. *Bell Syst. Tech. J.* **27**, 379–423 (1948).
8. Zhang, L., Fong, X., Chang, C.-H., Kong, Z. H. & Roy, K. Highly reliable memory-based physical unclonable function using spin-transfer torque MRAM. *2014 IEEE International Symposium on Circuits and Systems (ISCAS)* 2169–2172 (IEEE, 2014). doi:10.1109/ISCAS.2014.6865598
9. Dodda, A. *et al.* Graphene-based physically unclonable functions that are reconfigurable and resilient to machine learning attacks. *Nat. Electron.* **4**, 364–374 (2021).
10. Alharbi, A., Armstrong, D., Alharbi, S. & Shahrjerdi, D. Physically unclonable cryptographic primitives by chemical vapor deposition of layered MoS_2. *ACS Nano* **11**, 12772–12779 (2017).
11. Liu, R., Wu, H., Pang, Y., Qian, H. & Yu, S. Experimental characterization of physical unclonable function based on 1 kb resistive. *IEEE Electron Device Lett.* **36**, 1380–1383 (2015).
12. Zhang, R. *et al.* Nanoscale diffusive memristor crossbars as physical unclonable functions. *Nanoscale* **10**, 2721–2726 (2018).
13. Kim, K. *et al.* Voxelated opto-physically unclonable functions via irreplicable wrinkles. *Light Sci. Appl.* **12**, 245 (2023).
14. Lee, S. *et al.* Spintronic physical unclonable functions based on field-free spin–orbit-torque switching. *Adv. Mater.* **34**, 2203558 (2022).
15. Kim, D. *et al.* Reconfigurable electronic physically unclonable functions based on organic thin-film transistors with multiscale polycrystalline entropy for highly secure cryptography primitives. *Adv. Funct. Mater.* **33**, 2210367 (2023).
16. Hu, Z. *et al.* Physically unclonable cryptographic primitives using self-assembled carbon nanotubes. *Nat. Nanotechnol.* **11**, 559–565 (2016).
17. Zhong, D. *et al.* Twin physically unclonable functions based on aligned carbon nanotube arrays. *Nat. Electron.* **5**, 424–432 (2022).
18. Zhang, T. *et al.* Multimodal dynamic and unclonable anti-counterfeiting using robust diamond microparticles on heterogeneous substrate. *Nat. Commun.* **14**, 2507 (2023).
19. Kim, J. H. *et al.* Nanoscale physical unclonable function labels based on block

co-polymer self-assembly. *Nat. Electron.* **5**, 433–442 (2022).
20. Guo, H. *et al.* Multilevel encoding physically unclonable functions based on the multispecies structure in diamonds. *Adv. Funct. Mater.* 2304648 (2023). doi:10.1002/adfm.202304648
21. Zhang, J. *et al.* An all-in-one nanoprinting approach for the synthesis of a nanofilm library for unclonable anti-counterfeiting applications. *Nat. Nanotechnol.* **18**, 1027–1035 (2023).
22. Bay, H., Ess, A., Tuytelaars, T. & Van Gool, L. Speeded-up robust features (SURF). *Comput. Vis. Image Underst.* **110**, 346–359 (2008).
23. Wang, F. H., Yao, X. H. & Han, J. Q. Iris recognition based on multichannel Gabor filtering and feature fusion. *Guangdian Gongcheng/Opto-Electronic Eng.* **34**, 72–76 (2007).
