# Supplementary figures and images for: All-silicon multidimensionally-encoded optical physical unclonable functions for integrated circuit anti-counterfeiting (part 2 of 2)
Source: Nat Commun. 2024 Apr 13;15:3203. doi: 10.1038/s41467-024-47479-y (PMC11016093; doi:10.1038/s41467-024-47479-y)

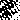

Supplement: Supplementary file 3 — Source Data [file 41467_2024_47479_MOESM3_ESM.zip › Source Data file/Coding for digitalization/Fig. 4/Same PUFs/20í┴20 (1).bmp]

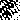

Supplement: Supplementary file 3 — Source Data [file 41467_2024_47479_MOESM3_ESM.zip › Source Data file/Coding for digitalization/Fig. 4/Same PUFs/20í┴20 (10).bmp]

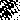

Supplement: Supplementary file 3 — Source Data [file 41467_2024_47479_MOESM3_ESM.zip › Source Data file/Coding for digitalization/Fig. 4/Same PUFs/20í┴20 (100).bmp]

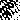

Supplement: Supplementary file 3 — Source Data [file 41467_2024_47479_MOESM3_ESM.zip › Source Data file/Coding for digitalization/Fig. 4/Same PUFs/20í┴20 (11).bmp]

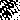

Supplement: Supplementary file 3 — Source Data [file 41467_2024_47479_MOESM3_ESM.zip › Source Data file/Coding for digitalization/Fig. 4/Same PUFs/20í┴20 (12).bmp]

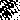

Supplement: Supplementary file 3 — Source Data [file 41467_2024_47479_MOESM3_ESM.zip › Source Data file/Coding for digitalization/Fig. 4/Same PUFs/20í┴20 (15).bmp]

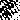

Supplement: Supplementary file 3 — Source Data [file 41467_2024_47479_MOESM3_ESM.zip › Source Data file/Coding for digitalization/Fig. 4/Same PUFs/20í┴20 (16).bmp]

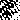

Supplement: Supplementary file 3 — Source Data [file 41467_2024_47479_MOESM3_ESM.zip › Source Data file/Coding for digitalization/Fig. 4/Same PUFs/20í┴20 (18).bmp]

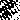

Supplement: Supplementary file 3 — Source Data [file 41467_2024_47479_MOESM3_ESM.zip › Source Data file/Coding for digitalization/Fig. 4/Same PUFs/20í┴20 (2).bmp]

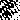

Supplement: Supplementary file 3 — Source Data [file 41467_2024_47479_MOESM3_ESM.zip › Source Data file/Coding for digitalization/Fig. 4/Same PUFs/20í┴20 (23).bmp]

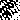

Supplement: Supplementary file 3 — Source Data [file 41467_2024_47479_MOESM3_ESM.zip › Source Data file/Coding for digitalization/Fig. 4/Same PUFs/20í┴20 (25).bmp]

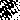

Supplement: Supplementary file 3 — Source Data [file 41467_2024_47479_MOESM3_ESM.zip › Source Data file/Coding for digitalization/Fig. 4/Same PUFs/20í┴20 (26).bmp]

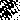

Supplement: Supplementary file 3 — Source Data [file 41467_2024_47479_MOESM3_ESM.zip › Source Data file/Coding for digitalization/Fig. 4/Same PUFs/20í┴20 (3).bmp]

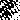

Supplement: Supplementary file 3 — Source Data [file 41467_2024_47479_MOESM3_ESM.zip › Source Data file/Coding for digitalization/Fig. 4/Same PUFs/20í┴20 (35).bmp]

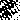

Supplement: Supplementary file 3 — Source Data [file 41467_2024_47479_MOESM3_ESM.zip › Source Data file/Coding for digitalization/Fig. 4/Same PUFs/20í┴20 (39).bmp]

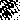

Supplement: Supplementary file 3 — Source Data [file 41467_2024_47479_MOESM3_ESM.zip › Source Data file/Coding for digitalization/Fig. 4/Same PUFs/20í┴20 (4).bmp]

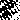

Supplement: Supplementary file 3 — Source Data [file 41467_2024_47479_MOESM3_ESM.zip › Source Data file/Coding for digitalization/Fig. 4/Same PUFs/20í┴20 (40).bmp]

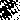

Supplement: Supplementary file 3 — Source Data [file 41467_2024_47479_MOESM3_ESM.zip › Source Data file/Coding for digitalization/Fig. 4/Same PUFs/20í┴20 (41).bmp]

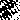

Supplement: Supplementary file 3 — Source Data [file 41467_2024_47479_MOESM3_ESM.zip › Source Data file/Coding for digitalization/Fig. 4/Same PUFs/20í┴20 (42).bmp]

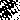

Supplement: Supplementary file 3 — Source Data [file 41467_2024_47479_MOESM3_ESM.zip › Source Data file/Coding for digitalization/Fig. 4/Same PUFs/20í┴20 (43).bmp]

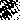

Supplement: Supplementary file 3 — Source Data [file 41467_2024_47479_MOESM3_ESM.zip › Source Data file/Coding for digitalization/Fig. 4/Same PUFs/20í┴20 (45).bmp]

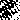

Supplement: Supplementary file 3 — Source Data [file 41467_2024_47479_MOESM3_ESM.zip › Source Data file/Coding for digitalization/Fig. 4/Same PUFs/20í┴20 (53).bmp]

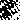

Supplement: Supplementary file 3 — Source Data [file 41467_2024_47479_MOESM3_ESM.zip › Source Data file/Coding for digitalization/Fig. 4/Same PUFs/20í┴20 (63).bmp]
