# Supplementary figures and images for: All-silicon multidimensionally-encoded optical physical unclonable functions for integrated circuit anti-counterfeiting (part 1 of 2)
Source: Nat Commun. 2024 Apr 13;15:3203. doi: 10.1038/s41467-024-47479-y (PMC11016093; doi:10.1038/s41467-024-47479-y)

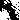

Supplement: Supplementary file 3 — Source Data [file 41467_2024_47479_MOESM3_ESM.zip › Source Data file/Coding for digitalization/Fig. 4/Different PUFs/20í┴20 (1).bmp]

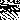

Supplement: Supplementary file 3 — Source Data [file 41467_2024_47479_MOESM3_ESM.zip › Source Data file/Coding for digitalization/Fig. 4/Different PUFs/20í┴20 (10).bmp]

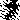

Supplement: Supplementary file 3 — Source Data [file 41467_2024_47479_MOESM3_ESM.zip › Source Data file/Coding for digitalization/Fig. 4/Different PUFs/20í┴20 (100).bmp]

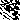

Supplement: Supplementary file 3 — Source Data [file 41467_2024_47479_MOESM3_ESM.zip › Source Data file/Coding for digitalization/Fig. 4/Different PUFs/20í┴20 (11).bmp]

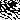

Supplement: Supplementary file 3 — Source Data [file 41467_2024_47479_MOESM3_ESM.zip › Source Data file/Coding for digitalization/Fig. 4/Different PUFs/20í┴20 (12).bmp]

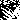

Supplement: Supplementary file 3 — Source Data [file 41467_2024_47479_MOESM3_ESM.zip › Source Data file/Coding for digitalization/Fig. 4/Different PUFs/20í┴20 (13).bmp]

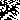

Supplement: Supplementary file 3 — Source Data [file 41467_2024_47479_MOESM3_ESM.zip › Source Data file/Coding for digitalization/Fig. 4/Different PUFs/20í┴20 (14).bmp]

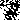

Supplement: Supplementary file 3 — Source Data [file 41467_2024_47479_MOESM3_ESM.zip › Source Data file/Coding for digitalization/Fig. 4/Different PUFs/20í┴20 (15).bmp]

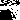

Supplement: Supplementary file 3 — Source Data [file 41467_2024_47479_MOESM3_ESM.zip › Source Data file/Coding for digitalization/Fig. 4/Different PUFs/20í┴20 (16).bmp]

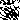

Supplement: Supplementary file 3 — Source Data [file 41467_2024_47479_MOESM3_ESM.zip › Source Data file/Coding for digitalization/Fig. 4/Different PUFs/20í┴20 (17).bmp]

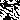

Supplement: Supplementary file 3 — Source Data [file 41467_2024_47479_MOESM3_ESM.zip › Source Data file/Coding for digitalization/Fig. 4/Different PUFs/20í┴20 (18).bmp]

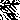

Supplement: Supplementary file 3 — Source Data [file 41467_2024_47479_MOESM3_ESM.zip › Source Data file/Coding for digitalization/Fig. 4/Different PUFs/20í┴20 (19).bmp]

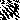

Supplement: Supplementary file 3 — Source Data [file 41467_2024_47479_MOESM3_ESM.zip › Source Data file/Coding for digitalization/Fig. 4/Different PUFs/20í┴20 (2).bmp]

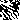

Supplement: Supplementary file 3 — Source Data [file 41467_2024_47479_MOESM3_ESM.zip › Source Data file/Coding for digitalization/Fig. 4/Different PUFs/20í┴20 (20).bmp]

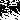

Supplement: Supplementary file 3 — Source Data [file 41467_2024_47479_MOESM3_ESM.zip › Source Data file/Coding for digitalization/Fig. 4/Different PUFs/20í┴20 (21).bmp]

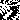

Supplement: Supplementary file 3 — Source Data [file 41467_2024_47479_MOESM3_ESM.zip › Source Data file/Coding for digitalization/Fig. 4/Different PUFs/20í┴20 (22).bmp]

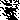

Supplement: Supplementary file 3 — Source Data [file 41467_2024_47479_MOESM3_ESM.zip › Source Data file/Coding for digitalization/Fig. 4/Different PUFs/20í┴20 (23).bmp]

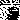

Supplement: Supplementary file 3 — Source Data [file 41467_2024_47479_MOESM3_ESM.zip › Source Data file/Coding for digitalization/Fig. 4/Different PUFs/20í┴20 (24).bmp]

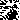

Supplement: Supplementary file 3 — Source Data [file 41467_2024_47479_MOESM3_ESM.zip › Source Data file/Coding for digitalization/Fig. 4/Different PUFs/20í┴20 (25).bmp]

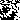

Supplement: Supplementary file 3 — Source Data [file 41467_2024_47479_MOESM3_ESM.zip › Source Data file/Coding for digitalization/Fig. 4/Different PUFs/20í┴20 (26).bmp]

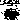

Supplement: Supplementary file 3 — Source Data [file 41467_2024_47479_MOESM3_ESM.zip › Source Data file/Coding for digitalization/Fig. 4/Different PUFs/20í┴20 (27).bmp]

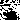

Supplement: Supplementary file 3 — Source Data [file 41467_2024_47479_MOESM3_ESM.zip › Source Data file/Coding for digitalization/Fig. 4/Different PUFs/20í┴20 (28).bmp]

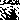

Supplement: Supplementary file 3 — Source Data [file 41467_2024_47479_MOESM3_ESM.zip › Source Data file/Coding for digitalization/Fig. 4/Different PUFs/20í┴20 (29).bmp]

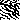

Supplement: Supplementary file 3 — Source Data [file 41467_2024_47479_MOESM3_ESM.zip › Source Data file/Coding for digitalization/Fig. 4/Different PUFs/20í┴20 (3).bmp]

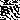

Supplement: Supplementary file 3 — Source Data [file 41467_2024_47479_MOESM3_ESM.zip › Source Data file/Coding for digitalization/Fig. 4/Different PUFs/20í┴20 (30).bmp]

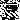

Supplement: Supplementary file 3 — Source Data [file 41467_2024_47479_MOESM3_ESM.zip › Source Data file/Coding for digitalization/Fig. 4/Different PUFs/20í┴20 (31).bmp]

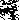

Supplement: Supplementary file 3 — Source Data [file 41467_2024_47479_MOESM3_ESM.zip › Source Data file/Coding for digitalization/Fig. 4/Different PUFs/20í┴20 (32).bmp]

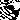

Supplement: Supplementary file 3 — Source Data [file 41467_2024_47479_MOESM3_ESM.zip › Source Data file/Coding for digitalization/Fig. 4/Different PUFs/20í┴20 (33).bmp]

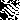

Supplement: Supplementary file 3 — Source Data [file 41467_2024_47479_MOESM3_ESM.zip › Source Data file/Coding for digitalization/Fig. 4/Different PUFs/20í┴20 (34).bmp]

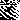

Supplement: Supplementary file 3 — Source Data [file 41467_2024_47479_MOESM3_ESM.zip › Source Data file/Coding for digitalization/Fig. 4/Different PUFs/20í┴20 (35).bmp]

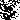

Supplement: Supplementary file 3 — Source Data [file 41467_2024_47479_MOESM3_ESM.zip › Source Data file/Coding for digitalization/Fig. 4/Different PUFs/20í┴20 (36).bmp]

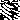

Supplement: Supplementary file 3 — Source Data [file 41467_2024_47479_MOESM3_ESM.zip › Source Data file/Coding for digitalization/Fig. 4/Different PUFs/20í┴20 (37).bmp]

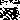

Supplement: Supplementary file 3 — Source Data [file 41467_2024_47479_MOESM3_ESM.zip › Source Data file/Coding for digitalization/Fig. 4/Different PUFs/20í┴20 (38).bmp]

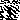

Supplement: Supplementary file 3 — Source Data [file 41467_2024_47479_MOESM3_ESM.zip › Source Data file/Coding for digitalization/Fig. 4/Different PUFs/20í┴20 (39).bmp]

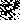

Supplement: Supplementary file 3 — Source Data [file 41467_2024_47479_MOESM3_ESM.zip › Source Data file/Coding for digitalization/Fig. 4/Different PUFs/20í┴20 (4).bmp]

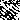

Supplement: Supplementary file 3 — Source Data [file 41467_2024_47479_MOESM3_ESM.zip › Source Data file/Coding for digitalization/Fig. 4/Different PUFs/20í┴20 (40).bmp]

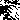

Supplement: Supplementary file 3 — Source Data [file 41467_2024_47479_MOESM3_ESM.zip › Source Data file/Coding for digitalization/Fig. 4/Different PUFs/20í┴20 (41).bmp]

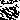

Supplement: Supplementary file 3 — Source Data [file 41467_2024_47479_MOESM3_ESM.zip › Source Data file/Coding for digitalization/Fig. 4/Different PUFs/20í┴20 (42).bmp]

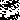

Supplement: Supplementary file 3 — Source Data [file 41467_2024_47479_MOESM3_ESM.zip › Source Data file/Coding for digitalization/Fig. 4/Different PUFs/20í┴20 (43).bmp]

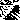

Supplement: Supplementary file 3 — Source Data [file 41467_2024_47479_MOESM3_ESM.zip › Source Data file/Coding for digitalization/Fig. 4/Different PUFs/20í┴20 (44).bmp]

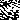

Supplement: Supplementary file 3 — Source Data [file 41467_2024_47479_MOESM3_ESM.zip › Source Data file/Coding for digitalization/Fig. 4/Different PUFs/20í┴20 (45).bmp]

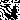

Supplement: Supplementary file 3 — Source Data [file 41467_2024_47479_MOESM3_ESM.zip › Source Data file/Coding for digitalization/Fig. 4/Different PUFs/20í┴20 (46).bmp]

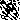

Supplement: Supplementary file 3 — Source Data [file 41467_2024_47479_MOESM3_ESM.zip › Source Data file/Coding for digitalization/Fig. 4/Different PUFs/20í┴20 (47).bmp]

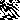

Supplement: Supplementary file 3 — Source Data [file 41467_2024_47479_MOESM3_ESM.zip › Source Data file/Coding for digitalization/Fig. 4/Different PUFs/20í┴20 (48).bmp]

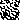

Supplement: Supplementary file 3 — Source Data [file 41467_2024_47479_MOESM3_ESM.zip › Source Data file/Coding for digitalization/Fig. 4/Different PUFs/20í┴20 (49).bmp]

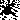

Supplement: Supplementary file 3 — Source Data [file 41467_2024_47479_MOESM3_ESM.zip › Source Data file/Coding for digitalization/Fig. 4/Different PUFs/20í┴20 (5).bmp]

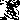

Supplement: Supplementary file 3 — Source Data [file 41467_2024_47479_MOESM3_ESM.zip › Source Data file/Coding for digitalization/Fig. 4/Different PUFs/20í┴20 (50).bmp]

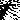

Supplement: Supplementary file 3 — Source Data [file 41467_2024_47479_MOESM3_ESM.zip › Source Data file/Coding for digitalization/Fig. 4/Different PUFs/20í┴20 (51).bmp]

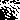

Supplement: Supplementary file 3 — Source Data [file 41467_2024_47479_MOESM3_ESM.zip › Source Data file/Coding for digitalization/Fig. 4/Different PUFs/20í┴20 (52).bmp]

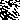

Supplement: Supplementary file 3 — Source Data [file 41467_2024_47479_MOESM3_ESM.zip › Source Data file/Coding for digitalization/Fig. 4/Different PUFs/20í┴20 (53).bmp]

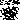

Supplement: Supplementary file 3 — Source Data [file 41467_2024_47479_MOESM3_ESM.zip › Source Data file/Coding for digitalization/Fig. 4/Different PUFs/20í┴20 (54).bmp]

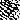

Supplement: Supplementary file 3 — Source Data [file 41467_2024_47479_MOESM3_ESM.zip › Source Data file/Coding for digitalization/Fig. 4/Different PUFs/20í┴20 (55).bmp]

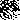

Supplement: Supplementary file 3 — Source Data [file 41467_2024_47479_MOESM3_ESM.zip › Source Data file/Coding for digitalization/Fig. 4/Different PUFs/20í┴20 (56).bmp]

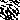

Supplement: Supplementary file 3 — Source Data [file 41467_2024_47479_MOESM3_ESM.zip › Source Data file/Coding for digitalization/Fig. 4/Different PUFs/20í┴20 (57).bmp]

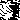

Supplement: Supplementary file 3 — Source Data [file 41467_2024_47479_MOESM3_ESM.zip › Source Data file/Coding for digitalization/Fig. 4/Different PUFs/20í┴20 (58).bmp]

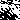

Supplement: Supplementary file 3 — Source Data [file 41467_2024_47479_MOESM3_ESM.zip › Source Data file/Coding for digitalization/Fig. 4/Different PUFs/20í┴20 (59).bmp]

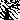

Supplement: Supplementary file 3 — Source Data [file 41467_2024_47479_MOESM3_ESM.zip › Source Data file/Coding for digitalization/Fig. 4/Different PUFs/20í┴20 (6).bmp]

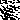

Supplement: Supplementary file 3 — Source Data [file 41467_2024_47479_MOESM3_ESM.zip › Source Data file/Coding for digitalization/Fig. 4/Different PUFs/20í┴20 (60).bmp]

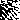

Supplement: Supplementary file 3 — Source Data [file 41467_2024_47479_MOESM3_ESM.zip › Source Data file/Coding for digitalization/Fig. 4/Different PUFs/20í┴20 (61).bmp]

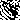

Supplement: Supplementary file 3 — Source Data [file 41467_2024_47479_MOESM3_ESM.zip › Source Data file/Coding for digitalization/Fig. 4/Different PUFs/20í┴20 (62).bmp]

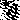

Supplement: Supplementary file 3 — Source Data [file 41467_2024_47479_MOESM3_ESM.zip › Source Data file/Coding for digitalization/Fig. 4/Different PUFs/20í┴20 (63).bmp]

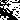

Supplement: Supplementary file 3 — Source Data [file 41467_2024_47479_MOESM3_ESM.zip › Source Data file/Coding for digitalization/Fig. 4/Different PUFs/20í┴20 (64).bmp]

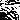

Supplement: Supplementary file 3 — Source Data [file 41467_2024_47479_MOESM3_ESM.zip › Source Data file/Coding for digitalization/Fig. 4/Different PUFs/20í┴20 (65).bmp]

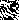

Supplement: Supplementary file 3 — Source Data [file 41467_2024_47479_MOESM3_ESM.zip › Source Data file/Coding for digitalization/Fig. 4/Different PUFs/20í┴20 (66).bmp]

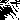

Supplement: Supplementary file 3 — Source Data [file 41467_2024_47479_MOESM3_ESM.zip › Source Data file/Coding for digitalization/Fig. 4/Different PUFs/20í┴20 (67).bmp]

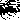

Supplement: Supplementary file 3 — Source Data [file 41467_2024_47479_MOESM3_ESM.zip › Source Data file/Coding for digitalization/Fig. 4/Different PUFs/20í┴20 (68).bmp]

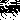

Supplement: Supplementary file 3 — Source Data [file 41467_2024_47479_MOESM3_ESM.zip › Source Data file/Coding for digitalization/Fig. 4/Different PUFs/20í┴20 (69).bmp]

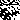

Supplement: Supplementary file 3 — Source Data [file 41467_2024_47479_MOESM3_ESM.zip › Source Data file/Coding for digitalization/Fig. 4/Different PUFs/20í┴20 (7).bmp]

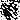

Supplement: Supplementary file 3 — Source Data [file 41467_2024_47479_MOESM3_ESM.zip › Source Data file/Coding for digitalization/Fig. 4/Different PUFs/20í┴20 (70).bmp]

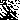

Supplement: Supplementary file 3 — Source Data [file 41467_2024_47479_MOESM3_ESM.zip › Source Data file/Coding for digitalization/Fig. 4/Different PUFs/20í┴20 (71).bmp]

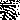

Supplement: Supplementary file 3 — Source Data [file 41467_2024_47479_MOESM3_ESM.zip › Source Data file/Coding for digitalization/Fig. 4/Different PUFs/20í┴20 (72).bmp]

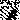

Supplement: Supplementary file 3 — Source Data [file 41467_2024_47479_MOESM3_ESM.zip › Source Data file/Coding for digitalization/Fig. 4/Different PUFs/20í┴20 (73).bmp]

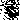

Supplement: Supplementary file 3 — Source Data [file 41467_2024_47479_MOESM3_ESM.zip › Source Data file/Coding for digitalization/Fig. 4/Different PUFs/20í┴20 (74).bmp]

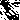

Supplement: Supplementary file 3 — Source Data [file 41467_2024_47479_MOESM3_ESM.zip › Source Data file/Coding for digitalization/Fig. 4/Different PUFs/20í┴20 (75).bmp]

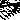

Supplement: Supplementary file 3 — Source Data [file 41467_2024_47479_MOESM3_ESM.zip › Source Data file/Coding for digitalization/Fig. 4/Different PUFs/20í┴20 (76).bmp]

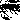

Supplement: Supplementary file 3 — Source Data [file 41467_2024_47479_MOESM3_ESM.zip › Source Data file/Coding for digitalization/Fig. 4/Different PUFs/20í┴20 (77).bmp]

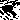

Supplement: Supplementary file 3 — Source Data [file 41467_2024_47479_MOESM3_ESM.zip › Source Data file/Coding for digitalization/Fig. 4/Different PUFs/20í┴20 (78).bmp]

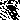

Supplement: Supplementary file 3 — Source Data [file 41467_2024_47479_MOESM3_ESM.zip › Source Data file/Coding for digitalization/Fig. 4/Different PUFs/20í┴20 (79).bmp]

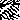

Supplement: Supplementary file 3 — Source Data [file 41467_2024_47479_MOESM3_ESM.zip › Source Data file/Coding for digitalization/Fig. 4/Different PUFs/20í┴20 (8).bmp]

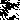

Supplement: Supplementary file 3 — Source Data [file 41467_2024_47479_MOESM3_ESM.zip › Source Data file/Coding for digitalization/Fig. 4/Different PUFs/20í┴20 (80).bmp]

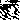

Supplement: Supplementary file 3 — Source Data [file 41467_2024_47479_MOESM3_ESM.zip › Source Data file/Coding for digitalization/Fig. 4/Different PUFs/20í┴20 (81).bmp]

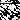

Supplement: Supplementary file 3 — Source Data [file 41467_2024_47479_MOESM3_ESM.zip › Source Data file/Coding for digitalization/Fig. 4/Different PUFs/20í┴20 (82).bmp]

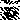

Supplement: Supplementary file 3 — Source Data [file 41467_2024_47479_MOESM3_ESM.zip › Source Data file/Coding for digitalization/Fig. 4/Different PUFs/20í┴20 (83).bmp]

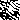

Supplement: Supplementary file 3 — Source Data [file 41467_2024_47479_MOESM3_ESM.zip › Source Data file/Coding for digitalization/Fig. 4/Different PUFs/20í┴20 (84).bmp]

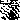

Supplement: Supplementary file 3 — Source Data [file 41467_2024_47479_MOESM3_ESM.zip › Source Data file/Coding for digitalization/Fig. 4/Different PUFs/20í┴20 (85).bmp]

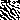

Supplement: Supplementary file 3 — Source Data [file 41467_2024_47479_MOESM3_ESM.zip › Source Data file/Coding for digitalization/Fig. 4/Different PUFs/20í┴20 (86).bmp]

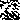

Supplement: Supplementary file 3 — Source Data [file 41467_2024_47479_MOESM3_ESM.zip › Source Data file/Coding for digitalization/Fig. 4/Different PUFs/20í┴20 (87).bmp]

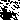

Supplement: Supplementary file 3 — Source Data [file 41467_2024_47479_MOESM3_ESM.zip › Source Data file/Coding for digitalization/Fig. 4/Different PUFs/20í┴20 (88).bmp]

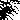

Supplement: Supplementary file 3 — Source Data [file 41467_2024_47479_MOESM3_ESM.zip › Source Data file/Coding for digitalization/Fig. 4/Different PUFs/20í┴20 (89).bmp]

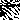

Supplement: Supplementary file 3 — Source Data [file 41467_2024_47479_MOESM3_ESM.zip › Source Data file/Coding for digitalization/Fig. 4/Different PUFs/20í┴20 (9).bmp]

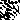

Supplement: Supplementary file 3 — Source Data [file 41467_2024_47479_MOESM3_ESM.zip › Source Data file/Coding for digitalization/Fig. 4/Different PUFs/20í┴20 (90).bmp]

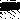

Supplement: Supplementary file 3 — Source Data [file 41467_2024_47479_MOESM3_ESM.zip › Source Data file/Coding for digitalization/Fig. 4/Different PUFs/20í┴20 (91).bmp]

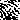

Supplement: Supplementary file 3 — Source Data [file 41467_2024_47479_MOESM3_ESM.zip › Source Data file/Coding for digitalization/Fig. 4/Different PUFs/20í┴20 (92).bmp]

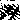

Supplement: Supplementary file 3 — Source Data [file 41467_2024_47479_MOESM3_ESM.zip › Source Data file/Coding for digitalization/Fig. 4/Different PUFs/20í┴20 (93).bmp]

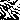

Supplement: Supplementary file 3 — Source Data [file 41467_2024_47479_MOESM3_ESM.zip › Source Data file/Coding for digitalization/Fig. 4/Different PUFs/20í┴20 (94).bmp]

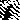

Supplement: Supplementary file 3 — Source Data [file 41467_2024_47479_MOESM3_ESM.zip › Source Data file/Coding for digitalization/Fig. 4/Different PUFs/20í┴20 (95).bmp]

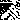

Supplement: Supplementary file 3 — Source Data [file 41467_2024_47479_MOESM3_ESM.zip › Source Data file/Coding for digitalization/Fig. 4/Different PUFs/20í┴20 (96).bmp]

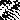

Supplement: Supplementary file 3 — Source Data [file 41467_2024_47479_MOESM3_ESM.zip › Source Data file/Coding for digitalization/Fig. 4/Different PUFs/20í┴20 (97).bmp]

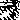

Supplement: Supplementary file 3 — Source Data [file 41467_2024_47479_MOESM3_ESM.zip › Source Data file/Coding for digitalization/Fig. 4/Different PUFs/20í┴20 (98).bmp]

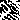

Supplement: Supplementary file 3 — Source Data [file 41467_2024_47479_MOESM3_ESM.zip › Source Data file/Coding for digitalization/Fig. 4/Different PUFs/20í┴20 (99).bmp]
