# Supplementary material for: All-silicon multidimensionally-encoded optical physical unclonable functions for integrated circuit anti-counterfeiting
Source: Nat Commun. 2024 Apr 13;15:3203. doi: 10.1038/s41467-024-47479-y (PMC11016093; doi:10.1038/s41467-024-47479-y)
Supplement: Supplementary file 2 — Peer Review File [file 41467_2024_47479_MOESM2_ESM.pdf]

nature portfolio

Peer Review File

All-silicon multidimensionally-encoded optical physical unclonable functions for integrated circuit anti-counterfeiting

Open Access This file is licensed under a Creative Commons Attribution 4.0 International License, which permits use, sharing, adaptation, distribution and reproduction in any medium or format, as long as you give appropriate credit to the original author(s) and the source, provide a link to the Creative Commons license, and indicate if changes were made. In the cases where the authors are anonymous, such as is the case for the reports of anonymous peer reviewers, author attribution should be to 'Anonymous Referee' followed by a clear attribution to the source work. The images or other third party material in this file are included in the article's Creative Commons license, unless indicated otherwise in a credit line to the material. If material is not included in the article's Creative Commons license and your intended use is not permitted by statutory regulation or exceeds the permitted use, you will need to obtain permission directly from the copyright holder. To view a copy of this license, visit http://creativecommons.org/licenses/by/4.0/.

REVIEWER COMMENTS

Reviewer #1 (Remarks to the Author):

In this article, the authors present a fascinating all-silicon multimodal physical unclonable functon(PUF) label. The performance of the multimodal label is demonstrated through 1) experimental and simulated data regarding material characterization, and 2) test results evaluating anticounterfeiting performance based on common standards. Overall, the demonstration encompasses comprehensive content, well-supported evidence, and robust scientific analysis. Additionally, the authors propose a detailed mutual authentication protocol that effectively addresses the device-server mutual issue. However, we have identified two potential issues with the article. We recommend publication in Nature Communications if the following two concerns can be addressed:

Q1. Upon reviewing the Method section, we found that the fabrication process appears complicated, involving numerous steps and various materials. This may result in 1) challenges in scalable fabrication, and 2) high production costs, both of which are critical barriers to the future application of PUF labels. Please explain why the complex fabrication process will not pose significant obstacles to the scalable production of PUF labels. Furthermore, please provide an estimate of the cost of fabricating PUF labels, based on the required materials and devices involved in the fabrication process.

Q2. In our view, the characteristics of the PUF label in this article are more akin to multidimensional encoding rather than multimodal. Multidimensional encoded labels (e.g., ACS Nano 15, 2901-2910 (2021); Nat. Commun. 11, 516 (2020)) typically exhibit the advantage of having much higher encoding capacity with a fixed image pixel number. In contrast, multimodal labels (e.g., Nat. Electron. 5, 433-442 (2022); Nat. Commun. 14, 2507 (2023)) possess modes with distinct characteristics, suitable for different application scenarios. In this article, the authors demonstrated six types of optical responses within the same image pixel, resulting in increased information entropy. Additionally, the authors did not specify the characteristics of different modes and their potential application scenarios.

If the authors concur with our perspective, please change the term 'multimodal' to 'multidimensional encoded' throughout the manuscript. Moreover, the authors should analyze the feasibility of extracting multidimensional information using low-cost, user-friendly devices, which is crucial for practical application. If the authors maintain that their label is indeed a multimodal label, please identify the potential application scenarios corresponding to the characteristics of the different modes.

Reviewer #2 (Remarks to the Author):

The authors present all-silica PUF devices with six responses for each pixel. They contribute efforts to address two challenges in the field: incompatibility with the CMOS process and limited information entropy. The PUF properties are well characterized, and the performance is acceptable. The explanation for position-dependent optical response is reasonable. Overall, the technology is sound. However, 1) its novelty requires justification, 2) the statistical analysis, stability test, and authentication process are not sufficient, and 3) a lot of details for experimental methods are missing. This manuscript needs to solve the following concerned issues before it can be considered for publication.

1. Regarding the main challenges this manuscript is working to solve, more elaboration is required to explain the difficulties and compare this work to the state-of-the-art. It would help to support the novelty of your work. Specifically,
a) For the compatibility of CMOS, lines 101-104 said that drop-casting and spin-coating

significantly benefited the compatibility of CMOS. A lot of PUFs are prepared by drop-casting or spin-coating, could the authors explain how this technology stands out? Discussions about why other reported PUFs are not compatible with CMOS and the specific requirements to make PUFs compatible with CMOS should be explained more clearly.
b) Fig. S4 compares the information entropy of this work and other reported work. However, the majority of the cited literature was published five years ago, so it cannot well represent the state-of-the-art.

2. For the six channels, general comments:
a) Are these six models totally independent of each other? For example, Si QD PL intensity (R2), Er3+ PL intensity (R3), and ratiometric PL intensity (R5). Fig. S8 gave the HD across six channels but how is it calculated? Considering the images from the six channels are not even the same data types, does it make sense to use HD to show the independence of the six channels?
b) The read time is an important parameter that needs to be considered for practical applications. Readout equipment and times of six security keys should be included in Table 1.
c) Specific thresholds are applied during the key generation. If the end users use different resolutions or equipment from different companies, will this threshold change? How can you guarantee a precise authentication in these scenarios? Especially the similarities are only around 80% when you use totally same equipment and settings.
d) Drop casting generally does not give a homogeneous film (e.g., coffee ring effect). Were the samples for statistical analysis collected from a localized area or representing different areas including edge and center? More samples should be involved for statistical analysis, typically at least 100.
e) Thermal, photo- and mechanical stability of the PUF devices should be tested.
Specific comments:
f) As explained in lines 191-192, emission from Er3+ is environmentally stable. Does that mean the distribution of Er3+ PL intensity (R3) on Si metamaterial would be not much different from that on bare Si substrate?
g) Fig. 4 characterized the Si QD PL wavelength (R4) channel. How were the 30 images generated? (e.g. Are they 1D spectra or 2D mapping? The 30 images in 4c are from 30 different position and the 30 images in 4d is from one position but 30 different time slots?)
h) Both similarity and HD were calculated based on binarized images, why do they have such a big difference? The average of the intra-HD is 0.09 in Fig. 4e, which makes the intra-correlation around 0.91. However, the similarity between the same PUFs in Fig. S7 is centered around 0.8. Either 0.91 or 0.8 doesn't give high reliability since not only one literature can achieve 0.98~0.99.
i) What's the Kolmogorov–Smirnov P-value for the Gaussian fit in Fig. 2e and 2g? They do not look like a nice fit, especially Fig. 2e, which suggests that the Si QD-related PL wavelength is not randomly distributed.

3. Please give details about the following experiments in the Methods part or supplementary information.
a) How the physical robustness of PUFs with/without PMMA was measured (Fig. S1c)? What is the concentration of the PMMA solution for spin coating?
b) At least one set of images for key generations during statistical analysis should be shown in SI.
c) How many samples and which view (top or 30° side) are measured for the statistical analysis in Fig. 2gh? What are the following data processes?
d) How was the simulation In lines 239-241 and Fig. S5d performed?
e) How was the exciting field map generated?
f) How were the metallic marks generated in Fig. S6?

Minor comments:
1. Full name needs to be given before the use of abbreviations. For example, PL.
2. The font in the figures needs to be uniform, including the SI figures.
3. Line 161, 'on the other hand…', means 'but' not 'and'.
4. In SI, line 35, there is no d in Fig. 3. It should be corrected.

Reviewer #3 (Remarks to the Author):

The authors present all-silicon multimodal optical PUFs based on Er-doped Si QDs and Si metasurface. The fabrication process of the PUFs is compatible with CMOS technologies. Given the six in-situ optical responses of the PUFs, exceptionally high information entropy has been achieved. The performance of the PUFs is impressive in terms of bit uniformity, similarity, Intra-HD, Inter-HD, FAR, FRR, and encoding capacity. I think that the current work holds good promise for the future development of the anti-counterfeiting of ICs. The manuscript is overall OK. However, I should ask the authors to improve the manuscript by considering the following points.

1. The authors in fact use the metasurface of a Si substrate. "Si metasurface" may be more appropriate than "Si metamaterial".
2. Do the authors refer to the length of the longest side of an inverted pyramid by saying "the length of maximum side"? For Fig. 1g it seems that the range of the length is not important. What matters is its average.
3. What is the full name of OR?
4. PL peaks at 1540 nm in Fig. 2d are too small to be clearly seen. Please provide a detailed caption for Fig. 2f.
5. Please clearly tell if a PUF is a 40 × 40 μm2 square. Does an image correspond to a PUF in Fig. 4 c&d?
6. More key information needs to be moved from Supplementary Note 3 to the main text.
7. The authors may show data to prove that the light emission from Er^{3+} in Er-Si QDs is environmentally stable (independent of position) in the Supplementary Information.
8. The English may be improved throughout the manuscript.

Reviewer #4 (Remarks to the Author):

The authors demonstrated a type of all-silicon-based anti-counterfeiting labels with multimodal optical physical unclonable functions (PUFs). The preparation of the PUF label is compatible with CMOS processes. The PUF pixels consist of Si metamaterial structure and Er^{3+} doped Si quantum dots (QDs). Different optical responses, including the micropattern imaging, Si QD PL intensity, Er^{3+} PL intensity, Si QD PL wavelength, Si QD PL lifetime, can be employed for multimode encoding, enabling the PUF labels with a high information entropy and encoding capacity. The research is interesting. The large-scale fabrication of silicon-based multimodal optical PUF labels should be very beneficial to the integrated circuit anti-counterfeiting and can be applied in other anti-counterfeiting areas. The work is of high novelty, but the current manuscript still needs to be improved before it can be published.

Detailed comments are as follows:

(1) The term "ratiometric PL intensity (R5)" is not clearly defined when it first appeared in the main text. It is the intensity ratio between the Si QD PL intensity (R2) and the Er^{3+} PL intensity (R3). R5 therefore is not an independent channel for encryption. The calculation of the encoding capacity should be corrected.
(2) The security key employing PL lifetime requires the instrumentation of microsecond lifetime measurement, which are usually not easy to access. Could the author comment on how to make use of such channel in regular applications?
(3) It is true that the light emission from Er^{3+} is environmentally stable. But the emission will be modulated by the optical density of states in the environment. The interplay between Si metamaterial and emission from Er^{3+} actually should be considered. For example, it has been reported that the emission from Er^{3+} can be modulated by the plasmon resonances of metal nanostructures (ACS Nano 2023, 17, 11, 10546–10559).
(4) The Si metamaterial structures should not support a localized surface plasmon at the wavelength of 405 nm. The authors are suggested to correct their statement (see line 243-246).
(5) The calculation of the information entropy seems wrong (please also refer to comment (1)).
(6) Could the authors estimate the cost of the PUF label?

POINT-BY-POINT RESPONSE TO REVIEWER COMMENTS

We express our gratitude to all the reviewers for their insightful feedback, which has been instrumental in refining our work. Please see below our detailed response to the comments, with the response in blue and the modifications in the revision in red.

REVIEWER COMMENTS

Reviewer #1:

In this article, the authors present a fascinating all-silicon multimodal physical unclonable function (PUF) label. The performance of the multimodal label is demonstrated through 1) experimental and simulated data regarding material characterization, and 2) test results evaluating anticounterfeiting performance based on common standards. Overall, the demonstration encompasses comprehensive content, well-supported evidence, and robust scientific analysis. Additionally, the authors propose a detailed mutual authentication protocol that effectively addresses the device-server mutual issue. However, we have identified two potential issues with the article. We recommend publication in Nature Communications if the following two concerns can be addressed:

Response:

We thank the reviewer for the constructive feedback and encouraging comments. We have comprehensively addressed each concern in our detailed point-by-point response below. Moreover, to better capture the two-dimensional (2D) nature of our PUF structure, which interacts with light at the surface, we have revised our manuscript to employ the term "Si metasurface" instead of "Si metamaterial".

Comment 1.

Upon reviewing the Method section, we found that the fabrication process appears complicated, involving numerous steps and various materials. This may result in 1) challenges in scalable fabrication, and 2) high production costs, both of which are critical barriers to the future application of PUF labels. Please explain why the complex fabrication process will not pose significant obstacles to the scalable production of PUF

labels. Furthermore, please provide an estimate of the cost of fabricating PUF labels, based on the required materials and devices involved in the fabrication process.

Response to comment 1.

We thank the reviewer for this very constructive comment. Here is our analysis addressing these concerns.

(1) Scalability analysis

Accessible Materials and Equipment: The materials and equipment we used are commonly available in the industry, with a wide range of suppliers listed in Table R1. This accessibility suggests that our method is capable of leveraging existing supply chains, demonstrating its potential for scalable production.

Established Processes: The processing techniques we employ, such as nonthermal plasma synthesis[1,2], wet chemical etching[3–5], drop-casting technique[6], and spin-coating encapsulation[3,5,7], are already widely used in the CMOS industry. This means our fabrication process can utilize the existing CMOS manufacturing infrastructure, which is crucial for large-scale production.

In summary, although our PUF label fabrication process involves multiple steps, each step relies on mature, readily available materials, equipment, and techniques that are compatible with existing CMOS processes. This compatibility, along with the potential for standardization and automation, proves the feasibility of scaling our production process.

(2) Cost estimation.

Here, to provide a general estimation of the total cost of fabricating the PUFs, we have calculated the cost of fabricating one piece 1.5×1.5 cm^2 sized PUF in almost all aspects based on our materials and devices, as shown in the following Table R1.

The estimated total cost for fabricating one piece of 1.5×1.5 cm^2 sized PUF label is about **1.45 USD**. Due to the high information entropy of our PUFs, a rather small working area can meet the purpose of anti-counterfeiting. In our study, the area used for encoding is 40 μm × 40 μm. Therefore, a 1.5×1.5 cm^2 sized PUF label can be divided into a maximum of $\sim 1.4 \times 10^5$ pieces of working labels. In this case, the cost of an individual working label will be significantly low (**~ 10^{-5} USD**). In addition, there

is potential for further reduction in costs through measures such as batch preparation, process optimization, and automation.

Our revision to the manuscript:

(Lines 117-126, Page 4: in the revised main text):

It is noteworthy that the scalability and cost-effectiveness of the fabrication process are paramount for the practical deployment of PUF-based anti-counterfeiting labels. The processing techniques adopted in this work such as nonthermal plasma synthesis[43,44], wet etching[45–47], drop casting[48] and spin coating[45,47,49] have already been widely used in the CMOS industry. This means that our fabrication process could be readily carried out based on the existing CMOS manufacturing infrastructure, which is crucial for large-scale production. Moreover, cost analysis reveals that the total cost for the fabrication of each PUF label is approximately ~ 10^{-5} USD, highlighting the economic viability of the current approach for widespread applications (Supplementary Table 1 and Supplementary Note 1).

Additionally, we included Table R1 as Supplementary Table 1 in the revised Supplementary Information as follows:

Table R1. The PUF fabrication process and estimation of detailed cost for fabricating one piece 1.5 × 1.5 cm^2 sized PUF.

Fabrication process	CMOS compatibility	Material/equipment		Unit price/ depreciation cost	Consumption	Cost (USD)	[a]The number of suppliers	Remarks
①Fabrication of Er-Si QDs toluene solution.	Compatible with plasma vapor deposition[1,2] and wet chemistry[3] in CMOS processes.	Material	SiH_4	14.88 USD/L	0.003 L	0.045	>100	Given that the fabrication of a single PUF with the size of 1.5 × 1.5 cm^2 necessitates 50 μL of Er-Si QDs in toluene solution, the consumption of materials and the depreciation cost of equipment are correspondingly proportional to the amounts and expenses required to prepare 50 μL of Er-Si QDs toluene solution. The depreciation cost of
			Ar	0.77 USD/L	0.3 L	0.23	>1000	
			$Er(tmhd)_3$	21 USD/g	0.0003 g	0.0063	>100	
			Glass tubes	0.42 USD/piece	1 piece	0.42	>6000	
			Methanol	4.39 USD/L	0.00005 L	0.00022	>100	
			HF	6.12 USD/L	0.000005 L	0.00003	>100	
			1-Dodecene	43.43 USD/L	0.00001 L	0.00043	>100	
			Mesitylene	21.47 USD/L	0.0001 L	0.0021	>100	
			Toluene	7.18 USD/L	0.00005 L	0.00036	>100	
		Equipment	$Er(tmhd)_3$ bottle	0.0018 USD/g	1 hour	0.0018	>6000	
			$Er(tmhd)_3$ Heater	0.00057 USD/hour	1 hour	0.00057	>6000	
			Vacuum Gauge	0.00046 USD/hour	1 hour	0.00046	>6000	

			Vacuum Pumps	0.00069 USD/hour	1 hour	0.00069	>6000	equipment is calculated over a 10-year period, with a residual value rate of 5%. The formula for calculating equipment depreciation cost is as follows: Equipment Depreciation Cost = [Cost of Equipment × (1 - 5%)] / (10 × 365 × 24 hours) × Actual Usage Time (hours). The same method is applied for calculating the depreciation costs of subsequent equipment.
			Pipeline Heaters	0.00036 USD/hour	1 hour	0.00036	>6000	
			Steel Tubing	0.000063 USD/hour	1 hour	0.000063	>6000	
			RF Power Supply	0.00046 USD/hour	1 hour	0.00046	>100	
			Matching box	0.000231 USD/hour	1 hour	0.000231	>100	
			Heating jacket	0.0000015 USD/hour	1 hour	0.0000015	>6000	
			Rotary evaporator	0.0000076 USD/hour	1 hour	0.0000076	>6000	
		Electricity	-	0.11 USD/kWh	0.03 kWh	0.0033	-	One person can prepare 10 mL of Er-Si QDs in toluene solution within 3 hours, then the actual time required to prepare 50 μL of the Er-Si QDs
		Labor	-	2.8 USD/hour	0.015 hours	0.042	-	

								in toluene solution is 0.015 hours. The average power of our equipment during the fabrication process is 2 kW
② Preparation of silicon metasurface	Compatible with wet chemical etching[3–5] in CMOS processes.	Material	Silicon wafer	0.07 USD/cm^2	2.25 cm^2	0.16	>100	Given that a single batch allows for the preparation of 60 pieces of PUFs with the size of 1.5 × 1.5 cm², the consumption of materials and the depreciation cost of equipment are respectively equivalent to the amounts and expenses required to fabricate a single PUF with the size of 1.5 × 1.5 cm².
			Acetone	6.38 USD/L	0.0017 L	0.011	>100	
			Ethanol	4.39 USD/L	0.0017 L	0.0075	>100	
			$Cu(NO_3)_2.3H_2O$	0.022 USD/L	0.055 L	0.0012	>100	
			HF	6.11 USD/L	0.0012 L	0.0073	>100	
			H_2O_2	6.91 USD/L	0.00096 L	0.0066	>100	
			HNO_3	6.91 USD/L	0.0017 L	0.012	>100	
			Deionized water	1.12 USD/L	0.0017 L	0.0019	>6000	
			N_2	0.24 USD/L	0.00083 L	0.0002	>100	
		Equipment	Ultrasonic machine	0.0000051 USD/hour	1 hour	0.0000051	>6000	
			Heating table	0.0000051 USD/hour	1 hour	0.0000051	>6000	

			Nitrogen Gun	0.0000026 USD/hour	1 hour	0.0000026	>6000	
		Electricity	-	0.11 USD/kWh	0.017 kWh	0.0019	-	One person is capable of preparing 60 PUFs within one hour, then the actual time required to prepare a single PUF is ~0.017 hours. The average power of our equipment during the fabrication process is 1 kW
		Labor	-	2.8 USD/hour	0.017 hour	0.048	-	
③ Encapsulation	Compatible with plastic encapsulation[3,5,7] in CMOS processes.	Material	PMMA	3080 USD/L	0.00005 L	0.15	>100	-
		Equipment	Pipette Gun	0.00003 USD/hour	1 hour	0.00003	>6000	
			Spin Coater	0.00045 USD/hour	1 hour	0.00045	>6000	
			Heating Station	0.00003 USD/hour	1 hour	0.00003	>6000	
		Electricity	-	0.11 USD/kWh	0.1kWh	0.011	-	One person is capable of encapsulating 10 PUFs within one hour, then the actual time
		Labor	-	2.8 USD/hour	0.1 hour	0.28	-	

								required to encapsulate a single PUF is 0.1 hours. The average power of our equipment during the fabrication process is 1 kW
Total						1.45		In the future, through measures such as batch preparation, process optimization, and automation, there is potential for further reduction in costs.

[a] The number of suppliers is totaled from Google, Alibaba, and Amazon.

Comment 2.

In our view, the characteristics of the PUF label in this article are more akin to multidimensional encoding rather than multimodal. Multidimensional encoded labels (e.g., ACS Nano 15, 2901-2910 (2021); Nat. Commun. 11, 516 (2020)) typically exhibit the advantage of having much higher encoding capacity with a fixed image pixel number. In contrast, multimodal labels (e.g., Nat. Electron. 5, 433-442 (2022); Nat. Commun. 14, 2507 (2023)) possess modes with distinct characteristics, suitable for different application scenarios. In this article, the authors demonstrated six types of optical responses within the same image pixel, resulting in increased information entropy. Additionally, the authors did not specify the characteristics of different modes and their potential application scenarios. If the authors concur with our perspective, please change the term 'multimodal' to 'multidimensional encoded' throughout the manuscript. If the authors maintain that their label is indeed a multimodal label, please identify the potential application scenarios corresponding to the characteristics of the different modes.

Response to comment 2.

We thank the reviewer for the insightful analysis and suggestions. After carefully reading the literature and conducting a thorough analysis of the characteristics of our PUF labels, we echo with the reviewer's viewpoint that the term 'multidimensionally-encoded' more accurately describes the characteristics of our PUF labels. We have replaced the term 'multimodal' with 'multidimensionally-encoded' throughout the entire manuscript.

Comment 3.

Moreover, the authors should analyze the feasibility of extracting multidimensional information using low-cost, user-friendly devices, which is crucial for practical application.

Response to comment 3.

We appreciate the reviewer for the insightful suggestion. We employed standard smartphones in conjunction with portable microscopes to capture the random

micropattern (R_1) of our PUF labels, as depicted in Fig. R1a. The anti-counterfeiting performance of our PUF based on R_1 are shown in Fig. R1b-g. The analysis of 100 different images yielded average bit uniformity (0.4988), similarity index (2.5%), and normalized Hamming distance (0.4992). Additionally, for a set of 100 same images, we calculated the average values of the similarity index (91.8%) and the Hamming distance (0.0933). Notably, both the false acceptance rate (FAR) and the false rejection rate (FRR) are remarkably low, which are at 8.8251×10^{-20} and 3.4265×10^{-20}, respectively (Fig. R1g). These results underscore the exceptional anti-counterfeiting performance of our PUF based on R_1, demonstrating that R_1 can be effectively extracted using low-cost and user-friendly devices.

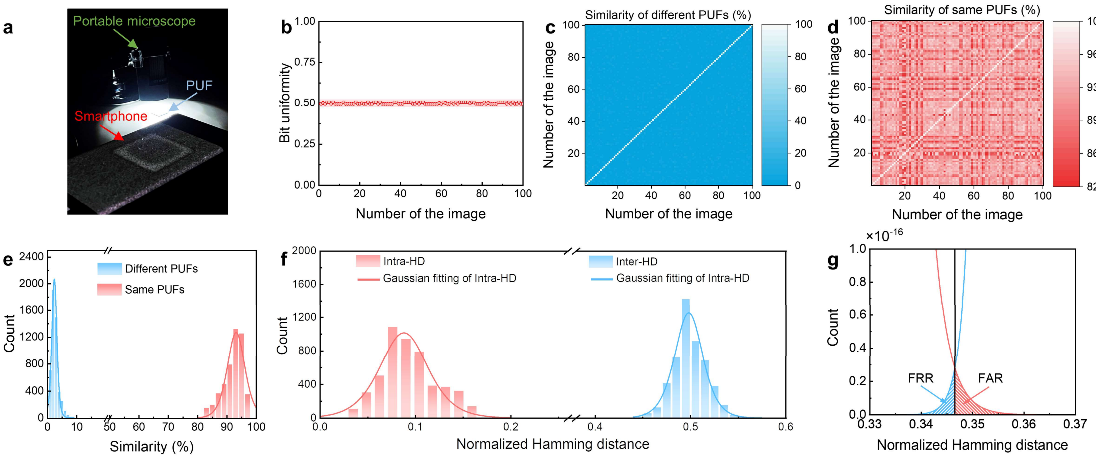

Fig. R1. a Experimental setup for capturing R_1 with a smartphone equipped with a portable microscope. **b** Occurrence probability of "1" in binary bits extracted from 100 images. **c** Pairwise match of 100 different PUFs using per pixel binary encoding of images acquired from the different position. **d** Pairwise match of 100 same PUFs using per pixel binary encoding of images acquired from the same position. **e** Distribution of the similarity for the different versus same PUFs in terms of per pixel binary encoding. **f** Distribution of normalized Hamming distance (HD) for Inter-HD and intra-HD. **g** Magnified Inter-HD and Intra-HD.

Regarding the extraction of information from other dimensions, such as the Si QD PL intensity (R_2), Er^{3+} PL intensity (R_3), Si QD PL wavelength (R_4), and Si QD PL lifetime (R_5), it is indeed challenging to directly obtain these signals through basic devices like

smartphones at present, due to the need for higher precision in spectral and temporal resolutions. It's important to note that the ratio signal (the ratio of R_2 to R_3) cannot be utilized as an independent signal, and thus has been excluded from our analysis (Please see the response to Reviewer 4 comment 1 for further details,). We envision that with advancements in portable spectrometer technology, continuous improvements in smartphone camera capabilities, and the development of fluorescence lifetime imaging techniques based on pulse sampling[8,9] and frequency domain[10,11], it will be possible to extract these complex signals through more straightforward devices in the near future. Furthermore, emerging analytical methods, such as phasor analysis[12,13] and Laguerre deconvolution[14,15], offer the potential for fit-free, rapid fluorescence lifetime imaging, significantly reducing dependence on specialized equipment and making the extraction of multidimensional information via low-cost, user-friendly means a reality.

In summary, we are actively exploring methods to extract multidimensional information from PUF labels using low-cost, user-friendly devices and we believe that, with the development of related technologies, effective extraction of all dimensional information will be achievable. We will continue to collaborate with experts and teams in the field to advance the technological progress in this area.

Our revision to the manuscript:

(Lines 370-378, Page 14: in the revised main text):

In addition, the feasibility of extracting optical responses using low-cost, user-friendly devices is essential for practical application. To this end, we have utilized standard smartphones in conjunction with portable microscopes to capture the R_1 of our PUF labels, as depicted in Supplementary Fig. 15, demonstrating excellent anti-counterfeiting performance. With the progress of portable spectrometer technology, the ongoing improvements of smartphone cameras, and the evolution of fluorescence imaging techniques based on pulse sampling[70,71] and frequency domain[72,73], we anticipate that the potential of extracting all the five optical responses by using even more affordable and user-friendly devices may be realized.

Furthermore, we added Fig. R1 as Supplementary Fig. 15 in the revised Supplementary Information.

Reviewer #2:

The authors present all-silica PUF devices with six responses for each pixel. They contribute efforts to address two challenges in the field: incompatibility with the CMOS process and limited information entropy. The PUF properties are well characterized, and the performance is acceptable. The explanation for position-dependent optical response is reasonable. Overall, the technology is sound. However, 1) its novelty requires justification, 2) the statistical analysis, stability test, and authentication process are not sufficient, and 3) a lot of details for experimental methods are missing. This manuscript needs to solve the following concerned issues before it can be considered for publication.

Response:

We appreciate the constructive feedback and have addressed the reviewer's concerns in our point-by-point response below.

Comment 1.

Regarding the main challenges this manuscript is working to solve, more elaboration is required to explain the difficulties and compare this work to the state-of-the-art. It would help to support the novelty of your work.

(a). For the compatibility of CMOS, lines 101-104 said that drop-casting and spin-coating significantly benefited the compatibility of CMOS. A lot of PUFs are prepared by drop-casting or spin-coating, could the authors explain how this technology stands out? Discussions about why other reported PUFs are not compatible with CMOS and the specific requirements to make PUFs compatible with CMOS should be explained more clearly.

Response to comment 1(a).

We thank the reviewer for pointing this out. Here, we would like to further clarify how our technology aligns with CMOS compatibility requirements and stands out from other PUF technologies.

CMOS Compatibility Requirements:

(1) Materials Compatibility: The materials utilized in PUF fabrication should be

compatible to the existing CMOS manufacturing ecosystem. Given its prevalent use in Si chips, Si emerges as the most compatible material within this ecosystem.

(2) Process Integration: The fabrication process for PUFs should leverage established CMOS manufacturing techniques, avoiding the need for supplementary, non-standard procedures.

Incompatibility of Other PUFs with CMOS:

A significant barrier to the integration of many existing PUF technologies into Si CMOS processes is the use of materials that do not align with the standard Si CMOS manufacturing ecosystem. The materials utilized in many PUF technologies, such as Ag nanoislands[16], nanocluster/graphene hybrid[17], Au networks[18], block copolymer self-assembly[19], and carbon dots[20], offer unique optical properties for PUF applications. However, these materials are not typically used in the standard Si CMOS manufacturing process. Techniques such as drop-casting or spin-coating of non-silicon materials, while simple and effective for creating PUFs, may introduce potential contamination to Si devices, making them difficult to be seamlessly integrated into the CMOS technology.

The Advantage of Our Technology and the Novelty of this work:

(1) Accessible Materials and Equipment: The materials and equipment we utilize are readily available in the Si CMOS manufacturing ecosystem, as detailed in Table R1 (Please refer to the Response to Comment 1 of Reviewer 1 for more details). This demonstrates a clear compatibility with CMOS processes.

(2) Utilization of Established Processes: The fabrication techniques we employ are well-established within the CMOS industry. Our use of plasma synthesis[1,2], wet chemical etching[3–5], drop-casting technique[6], and spin-coating encapsulation[3,5,7], means that our PUF fabrication process is fully compatible with existing CMOS manufacturing infrastructure, setting our technology apart.

By adhering to these principles, our PUF system not only demonstrates compatibility

with CMOS technology but also highlights how it differentiates from other PUFs that may not meet these stringent criteria. In conclusion, the novelty of our manuscript lies in its successful demonstration of a novel, scalable, and CMOS-compatible approach to fabricating optical PUFs with high information entropy. This work represents a significant leap forward in the development of secure, cost-effective anti-counterfeiting solutions for integrated circuits and IoT devices, highlighting the novelty of our research within the field.

Our revision to the manuscript:

(Lines 68-76, Page 3: in the revised main text):

This underscores the need for the development of multidimensionally-encoded optical PUFs that can leverage the unique properties of single-material systems (e.g., Ag nanoislands[29], nanocluster/graphene hybrids[30], Au networks[31], block copolymer self-assembly[21], diamond microparticles[32], and carbon dots[8]). Nevertheless, many reported multidimensionally-encoded optical PUFs involve materials that are not compatible with the standard silicon (Si) complementary metal-oxide-semiconductor (CMOS) platform. The incompatibility with CMOS manufacturing impedes the scalable integration of these PUFs to combat the counterfeiting of integrated circuits for IoT systems[1].

(b). Fig. S4 compares the information entropy of this work and other reported work. However, the majority of the cited literature was published five years ago, so it cannot well represent the state-of-the-art.

Response to comment 1(b).

We thank the reviewer for the insightful comment regarding the timeliness of the literature. We have conducted a thorough review and updated our comparative analysis. This included an extensive literature search for the most recent studies published within the last five years (Table R2). Our updated comparison, aspresented in Fig. R2, incorporates these findings, ensuring that our work is benchmarked against the latest advancements in the field.

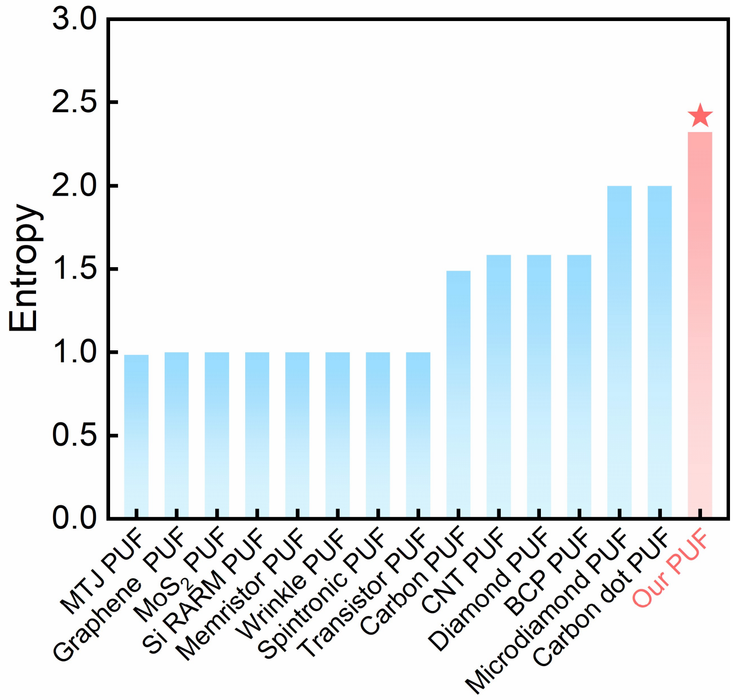

Fig. R2. A critical comparison of the information entropy of diverse PUFs, including MTJ PUF[21], graphene PUF[22], MoS_2 PUF[23], Si RARM PUF[24], memristor PUF[25], wrinkle PUF[26], spintronic PUF[27], transistor PUF[28], carbon PUF[29], carbon nanotube (CNT) PUF[30], diamond PUF[31], block co-polymer (BCP) PUF[19], microdiamond PUF[32], carbon dot PUF[20], and our PUF.

Furthermore, the recalculated information entropy value of the PUF in our work is **2.32 bits/pixel** (for further details, please refer to the response to Reviewer 4, Comment 5), which exceeds the information entropy reported in the majority of contemporary literature. This reaffirms the significance of our contribution, highlighting its superiority in terms of information entropy compared to most state-of-the-art reports.

Our revision to the manuscript:

(Lines 199-201, Page 8: in the revised main text):

Hence, an ultrahigh information entropy (up to 2.32 bits/pixel) is obtained (Supplementary Note 2), which is superior to those of PUFs based on alternative technologies[7,8,10,13,21,32–35,53–57] (Supplementary Fig. 4).

Furthermore, we added Fig. R2 as Supplementary Fig. 4 in the revised Supplementary

Information.

Table R2. Comparison of the information entropy of diverse PUFs.

Year	PUF device	Information Entropy	Refenrece
2014	MTJ PUF	0.985	R21
2021	graphene PUF	1	R22
2017	MoS_2 PUF	1	R23
2015	Si RARM PUF	1	R24
2018	memristor PUF	1	R25
2023	wrinkle PUF	1	R26
2022	spintronic PUF	1	R27
2023	transistor PUF	1	R28
2016	carbon PUF	1.49	R29
2022	carbon nanotube (CNT) PUF	1.58	R30
2023	diamond PUF,	1.58	R31
2022	block co-polymer (BCP) PUF	1.58	R19
2023	microdiamond PUF	2	R32
2023	carbon dot PUF	2	R20
**2024**	**our PUF**	**2.32**	**-**

Comment 2.

For the six channels, general comments:

(a). Are these six models totally independent of each other? For example, Si QD PL intensity (R_2), Er^{3+} PL intensity (R_3), and ratiometric PL intensity (R_5). Fig. S8 gave the HD across six channels but how is it calculated? Considering the images from the six channels are not even the same data types, does it make sense to use HD to show the independence of the six channels?

Response to comment 2(a).

We thank the reviewer for the comment regarding the independence of the six signal

channels in our study.

For the Independence of Signal Channels: We acknowledge that the ratiometric PL intensity (R_5), which is calculated as the ratio of Si QD PL intensity (R_2) to Er^{3+} PL intensity (R_3), does not constitute an independent encryption channel due to its dependence on R_2 and R_3. Consequently, we have revised our manuscript to exclude the ratiometric PL intensity as an independent signal. Instead, Si QD PL lifetime was designated as the R_5 signal, with subsequent references to R_5 pertaining to Si QD PL lifetime. This adjustment has led us to update our encoding capacity and information entropy calculations, resulting in revised values of 3.87×10^{279} for encoding capacity and 2.32 bits/pixel for information entropy, accurately reflecting our PUF system's capabilities.

Calculation of Hamming Distance (HD) across Different Channels:

(1) Image Generation: We first generated images for each signal—R_1 through R_5.

(2) Binary Encoding: Each image was converted into the binary format by setting thresholding signal intensities, assigning binary values (0 or 1) to translate diverse signal information into a binary image representation.

(3) HD Calculation: With the binary images for each signal, we then calculated the HD between pairs of images within one set (i.e. five types of signals). The HD is determined by comparing the binary values between two images and counting the number of positions where the values differ. The mean HD for a set of images is the average of these pairwise HD calculations.

(4) Aggregating Results Across Sets: To ensure statistical robustness, we repeated the above process for 100 different sets of data, each set representing the five types of signals obtained from different PUF samples or sample areas. For each set, we calculated the mean HD across the five channels.

(5) Mean HD Across Sets: The final step involved calculating the overall mean HD from the 100 sets of data. Remarkably, the aggregated mean HD value approached 0.5, indicating a balanced distribution of binary differences across all sets. This value, aligning with the ideal mean HD of 0.5, suggests a high degree of variability and independence among the five signals, as each channel contributes uniquely to the PUF characteristics without significant overlap or redundancy.

We have revised the normalized HD across the digitized keys of the five responses, as shown in Fig. R3.

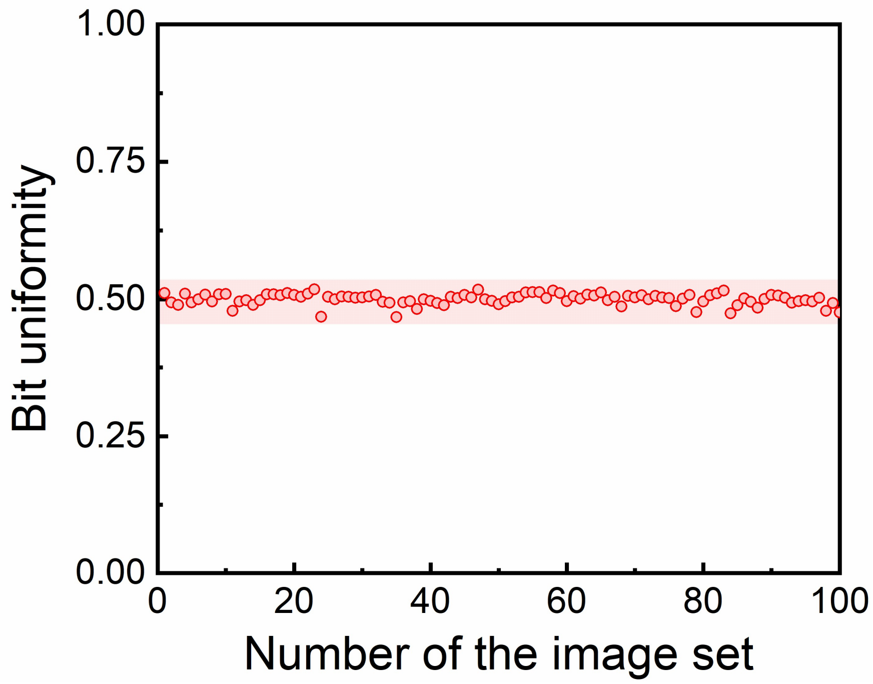

Fig. R3. The normalized Hamming distance (HD) across the digitized keys of the five responses.

For Using HD to Show Independence:

It is true that these channels do not represent the same data types—R_1 provides structural information; R_2 and R_3 offer intensity metrics; R_4 is related to spectral properties; R_5 provides temporal information. The use of HD in our study is grounded in the principle of quantifying dissimilarity between binary representations of data from each channel. While it is acknowledged that the channels produce different types of data—ranging from structural images to spectral and temporal signals—the conversion of these data into a binary format allows for a uniform basis of comparison. With the binary encoding process, we are able to assess the extent to which each channel contributes unique information to the overall PUF signature, even if the original data types are disparate. The underlying hypothesis is that if each channel indeed provides independent information, the binary representations derived from different PUFs should exhibit significant variability, as captured by HD. A higher HD implies greater dissimilarity, reinforcing the notion of channel independence in contributing to the PUF's complexity and security.

(b). The read time is an important parameter that needs to be considered for practical applications. Readout equipment and times of six security keys should be included in Table 1.

Response to comment 2(b).

We thank the reviewer for highlighting the importance of the readout equipment and time. We have updated Table 1 to include the readout equipment and times for the five signal channels (random micropatterns (R_1), PL intensity of Si QD (R_2), PL intensity of Er^{3+} (R_3), PL wavelength of Si QD (R_4), and PL lifetime of Si QD (R_5)) in the revised manuscript (Lines 368-370, Page 14). The readout time mentioned refers to the duration required to acquire a 20×20 pixels image for each signal type.

Table R3. The anti-counterfeiting performance and readout accessibility of our PUF for various optical responses.

Response	Bit uniformity	Intra-HD	Inter-HD	FAR	FRR	Readout	
						Equipment	Times
R_1	0.4989	0.0125	0.4989	2.0538×10^{-21}	1.4709×10^{-21}	Optical microscopy	1 s
R_2	0.4987	0.0062	0.5005	2.1088×10^{-36}	5.3115×10^{-36}	Confocal Raman System	40 s
R_3	0.4985	0.0063	0.4998	4.7162×10^{-31}	8.0269×10^{-31}		200 s
R_4	0.5006	0.0120	0.5004	2.4232×10^{-22}	3.1076×10^{-22}		40 s
R_5	0.4991	0.0106	0.5017	1.3949×10^{-16}	3.0477×10^{-16}		40 s

As presented in Table R3, the longest readout time is associated with the PL intensity of Er^{3+} (R_3), which requires 200 s. It is noteworthy that all signals can be rapidly read from their corresponding equipment, a feature that significantly enhances the applicability of our PUF technology for anti-counterfeiting purposes.

(c). Specific thresholds are applied during the key generation. If the end users use different resolutions or equipment from different companies, will this threshold change?

How can you guarantee a precise authentication in these scenarios? Especially the similarities are only around 80% when you use totally same equipment and settings.

Response to comment 2(c).

Regarding the Stability of the Threshold Values

We implemented a global search (GS) algorithm[33] to establish the optimal threshold for the digitization of each signal type. Our comprehensive analysis, as detailed in Table R4, examined the stability of these threshold values across a range of pixel resolutions (20 × 20, 50 × 50, 100 × 100 pixels) for all five signal types. The results demonstrate remarkable consistency in the threshold values across these varying resolutions, underscoring the robustness of our key generation methodology against changes in image resolution. Unfortunately, due to the constraints of the equipment, we were unable to directly compare the threshold variability across devices from different manufacturers. Despite this limitation, the inherent stability observed across different resolutions provides a strong indication that our PUF system's thresholds are fundamentally resilient to such variations.

Ensuring Precise Authentication

We have conducted a rigorous re-evaluation of our authentication process by retesting 100 images for each of the five signal types to verify their precisions in authentication. The results of this comprehensive analysis yielded significantly improved average similarities for each signal type: 98.68% for random micropatterns (R_1), 99.28% for PL intensity of Si QD (R_2), 99.32% for PL intensity of Er^{3+} (R_3), 98.73% for PL wavelength of Si QD (R_4), and 98.91% for PL lifetime of Si QD (R_5).

The observed increase in similarity percentages can be attributed to several factors that were optimized during the retesting process:

(1) Calibration and Standardization: We executed calibration of our equipment to more efficiently eliminate background noise and standardized the testing process to lessen mechanical disturbances during transitions between visible to near-infrared (vis-to-NIR) sensors. These steps ensured consistent experimental conditions across all tests, enhancing the reliability of our measurements.

Table R4. Thresholds comparison among different resolution.

Response	Resolution	Threshold
R_1	20 × 20	50.9981
	50 × 50	51.0217
	100 × 100	50.7624
R_2	20 × 20	55.7219
	50 × 50	55.2177
	100 × 100	55.0534
R_3	20 × 20	59.9319
	50 × 50	57.1827
	100 × 100	55.1081
R_4	20 × 20	144.5286
	50 × 50	143.9837
	100 × 100	144.6355
R_5	20 × 20	55.1127
	50 × 50	54.8281
	100 × 100	54.0397

(2) Enhanced Signal Processing Techniques: We fine-tuned the Gabor filtering parameters to improve local feature extraction and noise reduction, applying the formula[34]:

$$g_{\lambda,\theta,\varphi,\sigma,\gamma}(x,y) = e^{-\frac{x'^2+\gamma^2 y'^2}{2\sigma^2}} \cos\left(2\pi \frac{x'}{\lambda}\right) \qquad (R1)$$

with $x' = x\cos\theta + y\sin\theta$ and $y' = -x\sin\theta + y\cos\theta$, where x, y denote pixel coordinates; λ is the wavelength of the filter; θ is the orientation of the Gabor kernel; σ is the standard deviation of the Gaussian envelope; γ is the spatial aspect ratio. The optimized parameters for our Gabor filtering were: $\lambda = 3$, $\theta = 45°$, $\sigma = 1$, $\gamma = 1$. This has allowed for a more accurate representation of the original signal features, leading to higher similarity.

(3) Refined Threshold Determination: A global search algorithm[33] was employed to iteratively find the most suitable threshold for image binarization, resulting in a more uniform distribution of binary outcomes. This optimization ensured that the digital conversion process accurately reflected the unique characteristics of each PUF, contributing to the increased similarity.

Our revision to the manuscript:

(Lines 524-527, Page 19: in the revised main text):

Next, Gabor filtering (Supplementary Note 7) was applied to improve local feature extraction and noise reduction. Then, the image was transformed into the desired key size through binning operations. Finally, a global search algorithm[24] was employed to iteratively find the most suitable threshold for image binarization.

(d). Drop casting generally does not give a homogeneous film (e.g., coffee ring effect). Were the samples for statistical analysis collected from a localized area or representing different areas including edge and center? More samples should be involved for statistical analysis, typically at least 100.

Response to comment 2(d).

We thank the reviewer's valuable comment. The images used for analyzing the uniqueness of our PUFs were carefully selected from various regions across each sample, including both the edges and the center. This approach ensures that our analysis accurately reflects the heterogeneity of the PUF surface, including any effects from non-uniform film distribution such as the coffee ring effect. Additionally, for assessing the repeatability of our PUFs, we selected images from the same areas to minimize variability due to spatial differences. This dual strategy allows us to accurately gauge both the uniqueness and repeatability of our PUFs.

To further address the reviewer's concern, we took the reviewer's suggestion to expand our dataset to include 100 new images for each of the five signal types under investigation. This enhanced dataset capacity has been subjected to a thorough re-analysis, the results of which are now presented in Fig. R4-Fig. R8.

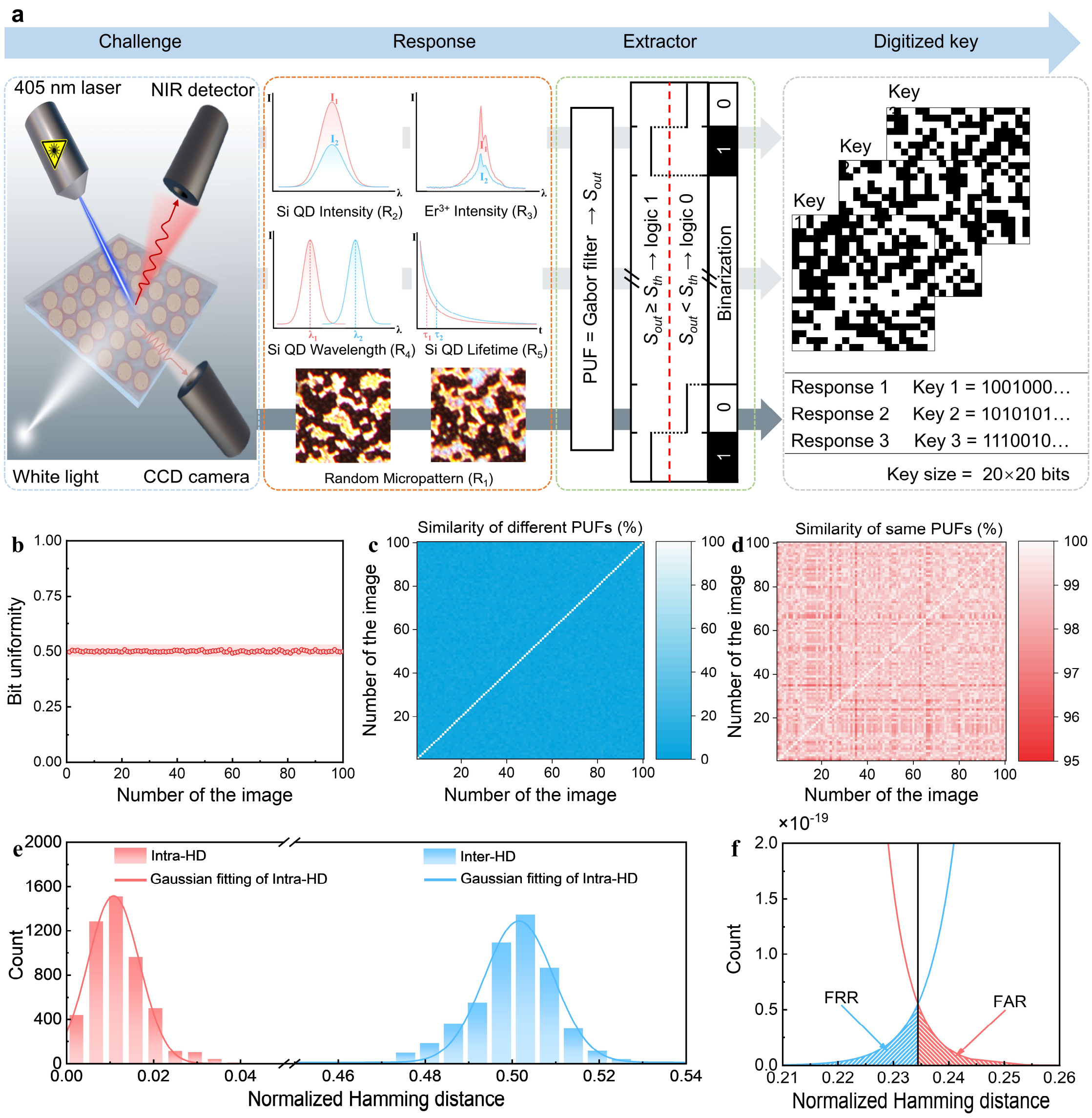

Fig. R4. Generated multiple security keys and features of a PUF based on the PL wavelength of Si QDs (R4). a Challenge-response authentication process for the PUF. **b** Occurrence probability of "1" in binary bits extracted from 100 images. **c** Pairwise match of 100 different PUFs using per pixel binary encoding of images acquired from the different positions. **d** Pairwise match of 100 same PUFs using per pixel binary encoding of images acquired from the same position. **e** Distribution of normalized Hamming distance (HD) for Inter-HD and intra-HD. **f** Magnified Inter-HD and Intra-HD.

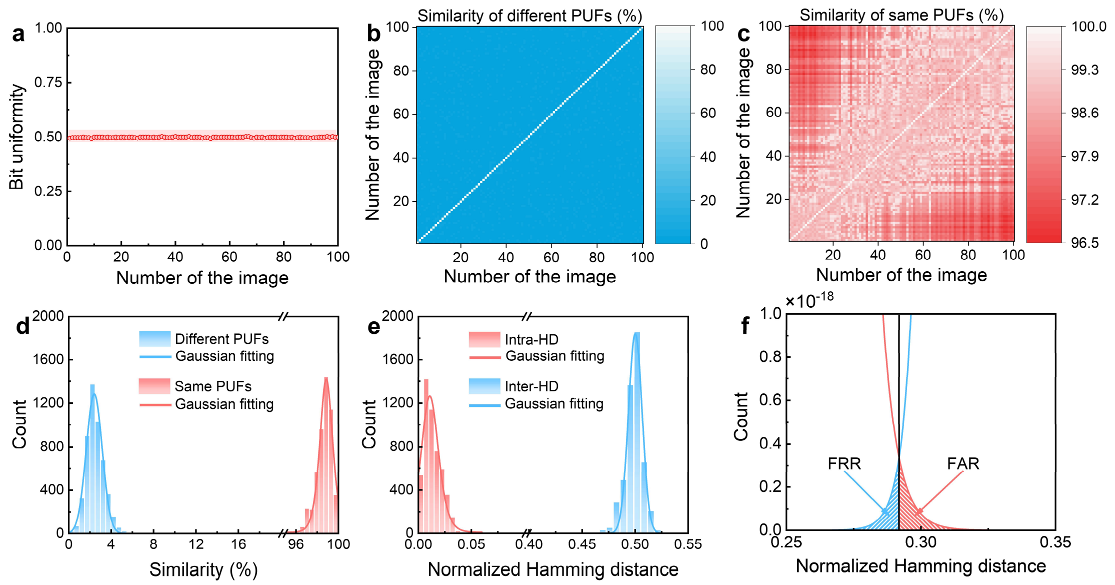

Fig. R5. Performance of the all-Si multidimensionally-encoded optical PUF based on the optical response of random micropattern(R_1). **a** Occurrence probability of "1" in binary bits extracted from 100 images. **b** Pairwise match of 100 different PUFs using per pixel binary encoding of images acquired from the different position. **c** Pairwise match of 100 same PUFs using per pixel binary encoding of images acquired from the same position. **d** Distribution of the similarity for the different versus same PUFs in terms of per pixel binary encoding. **e** Distribution of normalized Hamming distance (HD) for Inter-HD and intra-HD. **f** Magnified Inter-HD and Intra-HD.

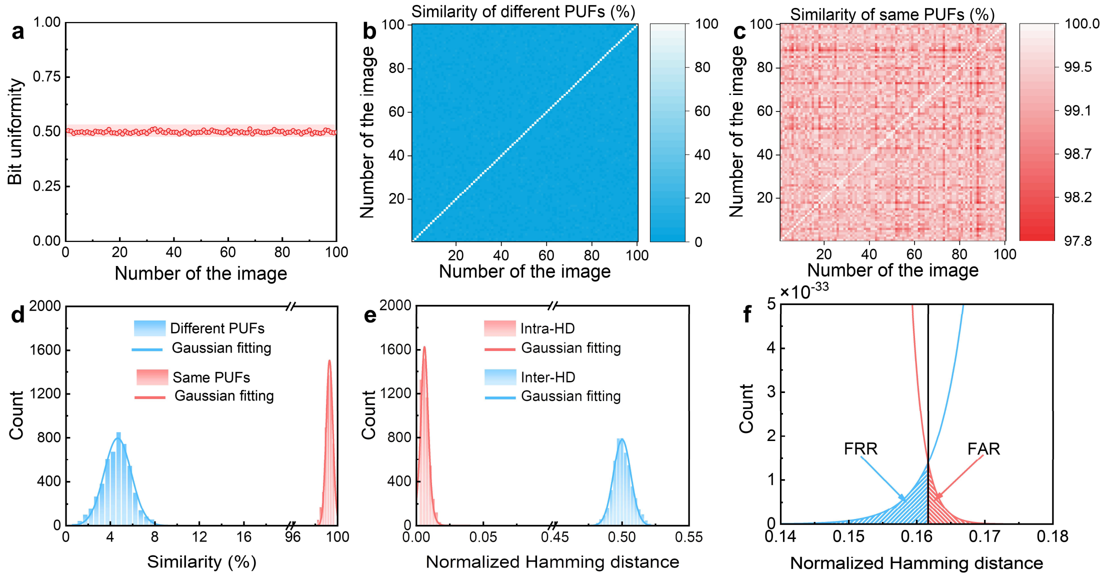

Fig. R6. Performance of the all-Si multidimensionally-encoded optical PUF based on the optical response of Si QD-related PL intensity (R_2). **a** Occurrence probability of "1" in binary bits extracted from 100 images. **b** Pairwise match of 100 different PUFs using per pixel binary encoding of images acquired from the different position. **c** Pairwise match of 100 same PUFs using per pixel binary encoding of images acquired from the same position. **d** Distribution of the similarity for the different versus same PUFs in terms of per pixel binary encoding. **e** Distribution of normalized Hamming distance (HD) for Inter-HD and intra-HD. **f** Magnified Inter-HD and Intra-HD.

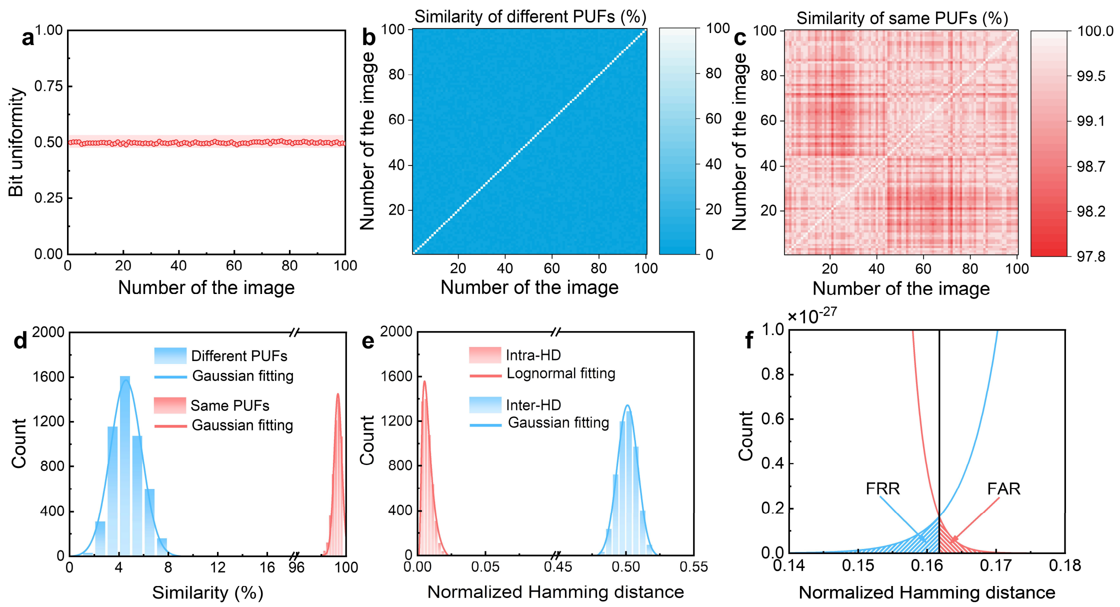

Fig. R7. Performance of the all-Si multidimensionally-encoded optical PUF based on the optical response of Er^{3+}-related emission intensity (R_3). **a** Occurrence probability of "1" in binary bits extracted from 100 images. **b** Pairwise match of 100 different PUFs using per pixel binary encoding of images acquired from the different position. **c** Pairwise match of 100 same PUFs using per pixel binary encoding of images acquired from the same position. **d** Distribution of the similarity for the different versus same PUFs in terms of per pixel binary encoding. **e** Distribution of normalized Hamming distance (HD) for Inter-HD and intra-HD. **f** Magnified Inter-HD and Intra-HD.

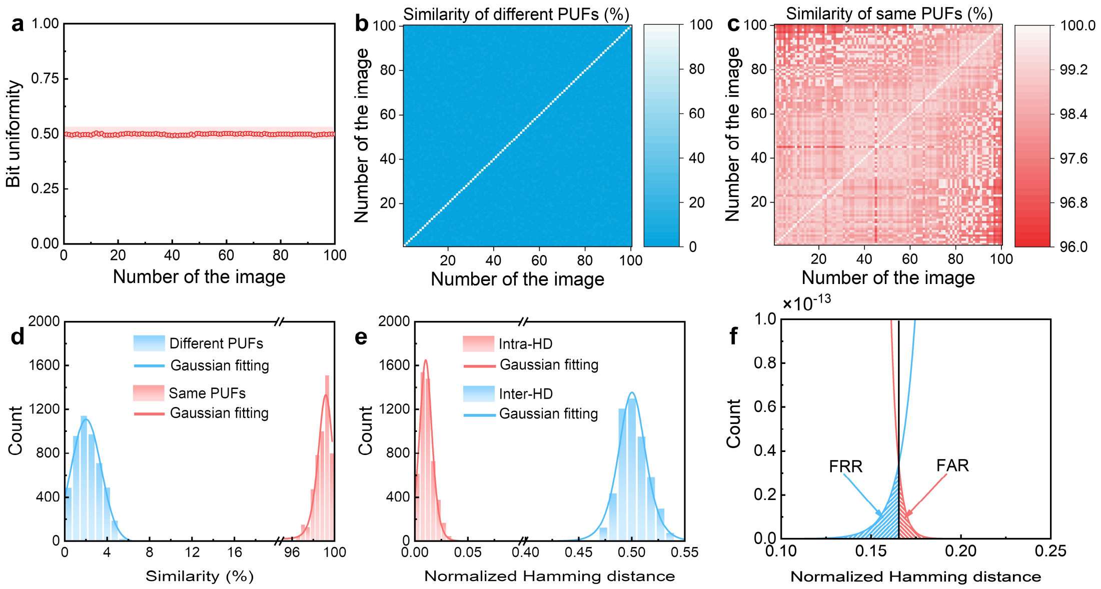

Fig. R8. Performance of the all-Si multidimensionally-encoded optical PUF based on the optical response of the Si QD-related PL lifetime (R_5). **a** Occurrence probability of "1" in binary bits extracted from 100 images. **b** Pairwise match of 100 different PUFs using per pixel binary encoding of images acquired from the different position. **c** Pairwise match of 100 same PUFs using per pixel binary encoding of images acquired from the same position. **d** Distribution of the similarity for the different versus same PUFs in terms of per pixel binary encoding. **e** Distribution of normalized Hamming distance (HD) for Inter-HD and intra-HD. **f** Magnified Inter-HD and Intra-HD.

(e). Thermal, photo- and mechanical stability of the PUF devices should be tested.

Response to comment 2(e).

We thank the reviewer's comment. Herein, we describe our comprehensive stability test results.

Thermal Stability:

We evaluated the thermal stability of five distinct signal types by comparing the similarity of images before and after heating (at 100°C for 1 hour) for each signal. This test was performed on five PUF devices for each signal type. The average similarity between pre- and post-heating images for each signal type exceeded 94% (Fig. R9), indicating high thermal stability.

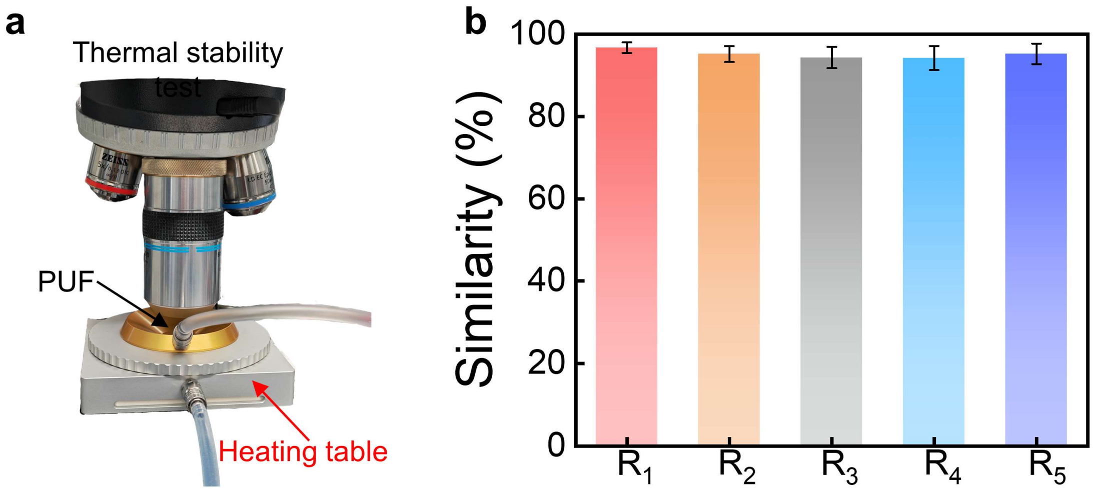

Fig. R9. Thermal stability measurement of the PUF devices. a Photograph of measurement setup for thermal stability test. **b** Average similarity of each optical response. Five PUF devices were tested for each optical response.

Photostability:

The photostability of the same five signal types was assessed by comparing the similarity across 100 consecutive images, with each signal type tested on five different PUF devices. The average similarity for each signal type remained above 98% (Fig. R10), demonstrating significant resistance to photodegradation over the testing period.

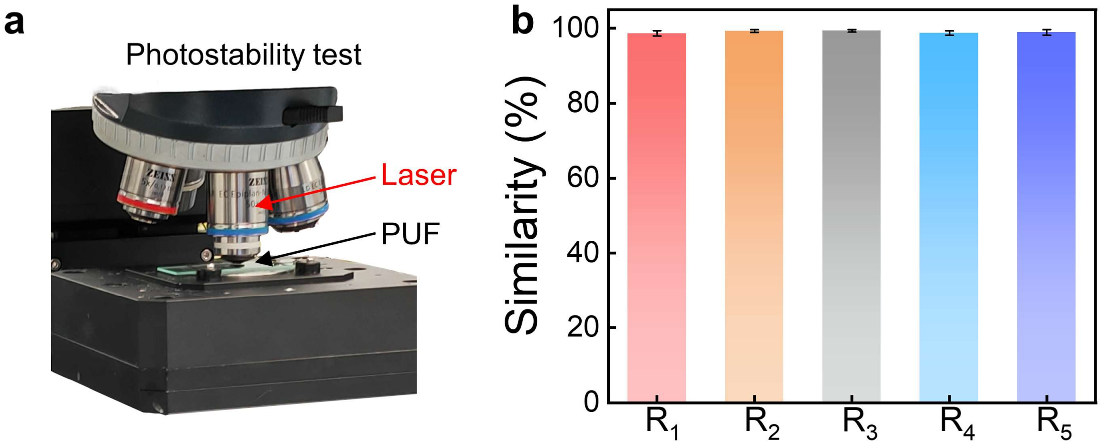

Fig. R10. Photostability measurement of the PUF devices. a Photograph of measurement setup for photostability test. **b** Average similarity of each optical response. Each optical response was tested 100 times.

Mechanical Stability:

To test the mechanical stability, we compared the similarity of images before and after subjecting each of the five signal types to friction in quartz sand. This procedure was repeated for five PUF devices per signal type. The average similarity post-friction for each signal type was greater than 94% (Fig. R11), showcasing excellent mechanical durability.

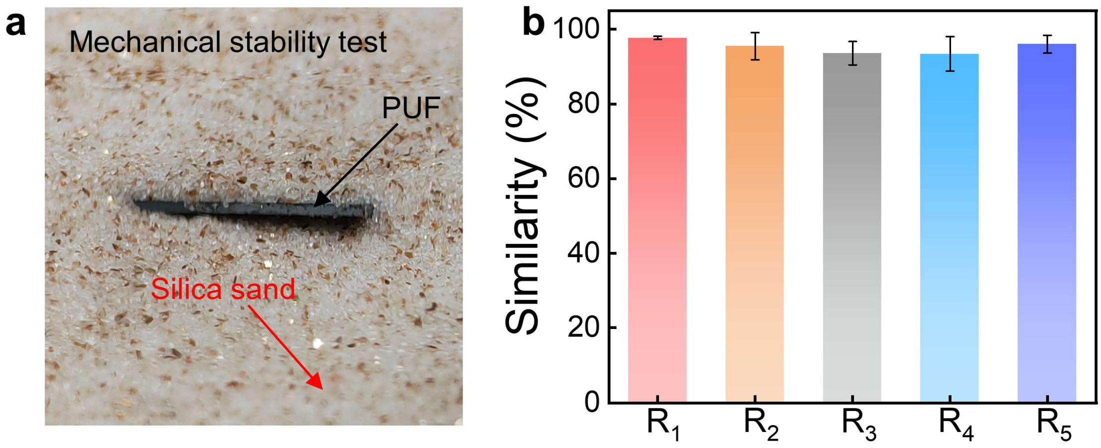

Fig. R11. Mechanical stability measurement of the PUF devices. a Photograph of measurement setup for mechanical stability test. **b** Average similarity of each optical response. Five PUF devices were tested for each optical response.

UV Stability:

Finally, the UV stability of each signal type was tested by comparing image similarity before and after UV exposure (365 nm, 10W for 1 hour). This test was also conducted on five PUF devices for each signal type. The average similarity remained above 95% post-exposure (Fig. R12), affirming the devices' robustness against UV radiation.

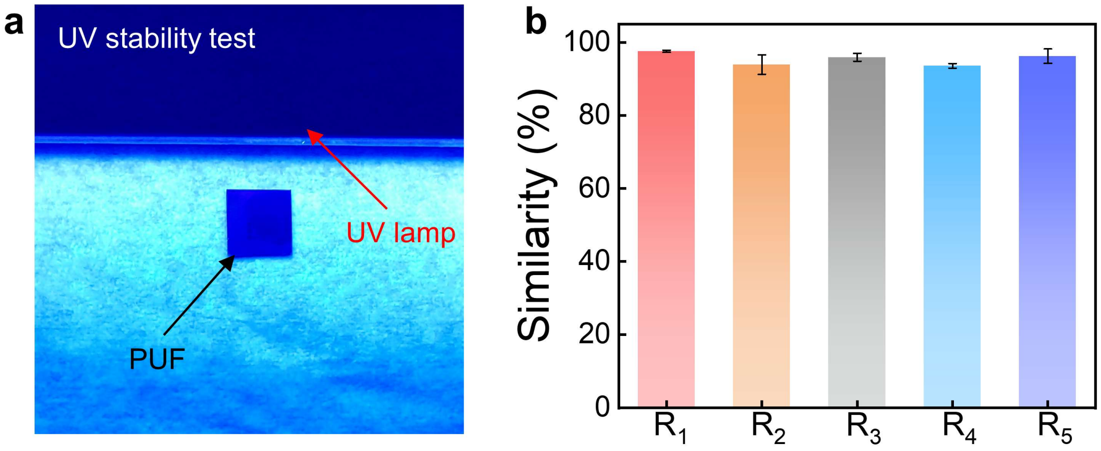

Fig. R12. UV stability measurement of the PUF devices. a Photograph of measurement setup for UV stability test. **b** Average similarity of each optical response. Five PUF devices were tested for each optical response.

These results collectively affirm the high degree of stability of our PUF devices under thermal, photonic, mechanical, and UV stressors. The consistent high similarity across different testing conditions underscore the reliability of our PUFs in maintaining their unique signatures, making them highly suitable for various security and authentication applications.

Our revision to the manuscript:

(Lines 392-405, Page 15: in the revised main text):

We have also validated the robustness of our PUFs to heating, abrasion, UV radiation, and laser exposure (Supplementary Figs. 16-19). Thermal stability is examined by heating the PUFs to 100°C for 1 hour and comparing pre-heating and post-heating images. The average similarity between pre- and post-heating images exceeds 94% for each optical response (Supplementary Fig. 16). Photostability is verified by acquiring 100 consecutive images under 405 nm laser illumination. The average similarity remains above 98% for all optical responses (Supplementary Fig. 17). Mechanical durability is evaluated by abrading the PUFs with quartz sands. The average post-abrasion similarity is greater than 94% for each type of signal (Supplementary Fig. 18). Finally, UV stability is tested by exposing the PUFs to 365 nm UV radiation at 10 W

for 1 hour. The average similarity remains above 95% post-exposure (Supplementary Fig. 19). All these results validate the remarkable stability of our all-Si multidimensionally-encoded PUFs under thermal, mechanical, UV, and laser conditions.

(f). As explained in lines 191-192, emission from Er^{3+} is environmentally stable. Does that mean the distribution of Er^{3+} PL intensity (R_3) on Si metamaterial would be not much different from that on bare Si substrate?

Response to comment 2(f).

We thank the reviewer for the insightful comment. We apologize that our previous descriptions might have led to some confusion. To clarify, while the PL wavelength and lifetime of Er^{3+} are environmentally stable and exhibit minimal variation with changing conditions, the PL intensity can vary depending on the surrounding environment (Details can be seen in Reviewer 3 Comment 7). To explore this further, we conducted Er^{3+} PL mapping tests on devices featuring both Si metasurface and bare Si substrate to compare the distribution of Er^{3+} PL intensity. Analyzing 100 images from each type of device, we focused on their bit uniformity as a measure of signal randomness and uniform distribution. The results of our analysis revealed that PL mapping images from devices with Si metasurface showed an average bit uniformity of 0.4985 with a standard deviation of 0.0036 (Fig. R13). In contrast, PL mapping images from devices with a bare Si substrate exhibited an average bit uniformity of 0.5047 with a standard deviation of 0.0274 (Fig. R13). These findings indicate that PL mapping images from the Si metasurface devices possess greater randomness and chaos compared to those from the bare Si substrate.

This increased randomness and chaos in the Si metasurface -based PUF could likely be attributed to the intricate and disordered structures present on the surface of the Si metasurface, which enhances the signal's complexity and randomness[35]. Despite the inherent environmental stability of Er^{3+} PL wavelength and lifetime (Details can be seen in Reviewer 3 Comment 7), our study demonstrates that the underlying substrate—

specifically, the presence of a metasurface structure—can significantly influence the distribution of Er^{3+} PL intensity.

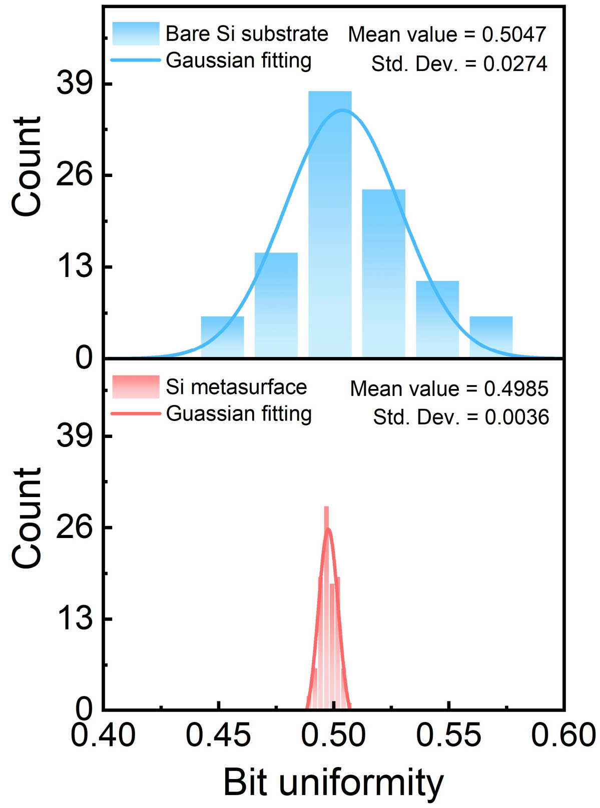

Fig. R13. Comparison of bit uniformity distribution for Er^{3+} PL intensity (R_3) derived from Si metasurface (bottom) and bare Si substrate (top). Each distribution is based on analyses of 100 images.

Our revision to the manuscript:

(Lines 174-176, Page 6: in the revised main text):

In particular, the greater randomness of R_3 derived from the Si metasurface compared to those from the bare Si substrate (Supplementary Fig. 3) well indicates the interplay between the Si metasurface and R_3.

(Lines 218-226, Page 8: in the revised main text):

To figure out the coupling mechanism between the Si metasurface and Er-Si QDs, FDTD simulations are employed. These Er-Si QDs contain two emitters: Er^{3+} and Si

QDs. Owing to the shielding of the 4*f* shell orbital by the outer 5*s* and 5*p* orbitals for Er^{3+} (ref. [58]), the PL wavelength and lifetime of Er^{3+} in Er-Si QDs exhibit rather weak sensitivity to environmental changes (Supplementary Fig. 5), limiting their use for anti-counterfeiting. Thus, the following FDTD simulations focus on the optical responses related to the band-to-band transitions of Er-Si QDs (i.e., the PL emission of Si QDs), particularly in relation to the interaction with the Si metasurface.

Furthermore, we added Fig. R13 as Supplementary Fig. 3 in the revised Supplementary Information.

(g). Fig. 4 characterized the Si QD PL wavelength (R_4) channel. How were the 30 images generated? (e.g. Are they 1D spectra or 2D mapping? The 30 images in 4c are from 30 different position and the 30 images in 4d is from one position but 30 different time slots?)

Response to comment 2(g).

We thank the reviewer for pointing this out. The images characterized in Fig. 4 were generated through a two-step process utilizing equipment designed for PL mapping to create 2D images:

(1) 2D PL Mapping: Initially, we conducted 2D PL mapping to capture PL information across our PUFs. Each 2D PL map covered an area of 40 μm × 40 μm with a resolution of 20 × 20 pixels. Crucially, each pixel within these maps contained complete spectral information, including both intensity and wavelength data.

(2) Wavelength Signal Extraction: Utilizing WITec's proprietary software, we extracted the central wavelength for each pixel. The central wavelength was determined based on the peak PL intensity value within the spectrum, thereby generating a PL wavelength mapping image that accurately represents the distribution of PL wavelengths across the sample.

Regarding the images in Fig. 4c, these were derived from different positions across the PUF samples, incorporating different regions to ensure a comprehensive analysis. This selection method, including both edge and center areas, was deliberately chosen to accurately reflect the surface heterogeneity of the PUFs and account for potential non-uniformities, such as those caused by the coffee ring effect.

Regarding the images in Fig. 4d, focusing on repeatability, we selected images from the same areas but captured at different time slots. This approach was aimed at minimizing variability that could arise from spatial differences, thereby providing a clearer insight into the temporal stability and repeatability of the PL wavelength signal (R_4) across multiple measurements.

Our revision to the manuscript:

(Line 288, Page 11: in the revised main text):

Each PUF is designed to be represented by a square image with the size of 40 × 40 μm^2.

(Lines 321-323, Page 12: in the revised main text):

Fig. 4c: Pairwise match of 100 different PUFs using per pixel binary encoding of images acquired from the different positions.

Fig. 4d:Pairwise match of 100 same PUFs using per pixel binary encoding of images acquired from the same position.

(h). Both similarity and HD were calculated based on binarized images, why do they have such a big difference? The average of the intra-HD is 0.09 in Fig. 4e, which makes the intra-correlation around 0.91. However, the similarity between the same PUFs in Fig. S7 is centered around 0.8. Either 0.91 or 0.8 doesn't give high reliability since not only one literature can achieve 0.98~0.99.

Response to comment 2(h).

We thank the reviewer for the valuable comment. The discrepancy between the intra-correlation (0.91 as derived from an average HD of 0.09) and the similarity centered around 0.8 can be attributed to the distinct nature of the mathematical definition and their sensitivity to the binarization process:

1. Similarity Calculation: The process involves digitizing the PUF images into binary matrices, followed by a pairwise comparison to calculate the similarity between images.

The similarity can be mathematically defined as follows:

$$Similarity = \frac{\boldsymbol{C_0}}{\boldsymbol{C}} \qquad (R2)$$

where $\boldsymbol{C}$ is the matrix difference between two PUF images after binary digitalization. The digitized matrices of two PUF images can be represented as: $\mathbf{A} = \begin{bmatrix} a_{11} & \cdots & a_{1n} \\ \vdots & \ddots & \vdots \\ a_{m1} & \cdots & a_{mn} \end{bmatrix}$, $\mathbf{B} = \begin{bmatrix} b_{11} & \cdots & b_{1n} \\ \vdots & \ddots & \vdots \\ b_{m1} & \cdots & b_{mn} \end{bmatrix}$, $\mathbf{C} = \mathbf{A} - \mathbf{B} = \begin{bmatrix} a_{11} - b_{11} & \cdots & a_{1n} - b_{1n} \\ \vdots & \ddots & \vdots \\ a_{m1} - b_{m1} & \cdots & a_{mn} - b_{mn} \end{bmatrix}$. $\boldsymbol{C_0}$ is the number of "0" in matrix $\boldsymbol{C}$.

This method assesses the percentage of matching pixels between two PUFs, providing a direct measure of similarity. The initial similarity value centered around 0.8 might have been influenced by the specific thresholds set during the digitization. The similarity metric is particularly useful for visualizing the overall resemblance between images, making it an accessible and easy-to-interpret measure for general similarity assessment.

2. HD Calculation: HD quantifies the minimum number of bit substitutions required to convert one binary key into another. The Intra-HD can be mathematically defined as follows:

$$Intra - Hamming\ distance = \frac{1}{m}\sum_{t=1}^{m}\frac{HD(K_i, K_{i,t})}{s} \qquad (R3)$$

where K_i and $K_{i,t}$ are the original s-bit reference key and a s-bit key extracted from the same PUF device at a different time-point t and m is the number of repeated measurements. For a PUF label represented by a 20 × 20 pixel matrix, this translates into a 20-bit key per row, with a total of 4950 pairwise comparisons (C_{100}^{2}) conducted across the dataset. The HD approach focuses on the bit-level differences between keys, offering a granular view of dissimilarity. HD is especially valuable in contexts where the exact alignment and distribution of features within the images are crucial, such as in cryptographic applications and error correction coding.

Upon conducting a thorough reevaluation and recalculating the metrics for 100 images, the results yielded significantly improved average similarities for each signal type: 98.68% for random micropatterns (R_1), 99.28% for PL intensity of Si QD (R_2), 99.32% for PL intensity of Er^{3+} (R_3), 98.73% for PL wavelength of Si QD (R_4), and 98.91% for PL lifetime of Si QD (R_5). These recalculated values underscore the high reliability of our PUF system, closely aligning with the high standards of 0.98~0.99 reported in the literature.

In summary, the initial discrepancy arose from the differential sensitivities of the similarity and HD metrics to the binarization parameters used in our analysis. The recalculated similarity, derived from a refined approach to image processing and comparison (Please refer to the Response to Comment 2c of Reviewer 2 for more details.), better reflect the inherent reliability and robustness of our PUFs.

(i). What's the Kolmogorov–Smirnov P-value for the Gaussian fit in Fig. 2e and 2g? They do not look like a nice fit, especially Fig. 2e, which suggests that the Si QD-related PL wavelength is not randomly distributed.

Response to comment 2(i).

We thank the reviewer for this valuable comment. Initially, the K-S P-values calculated for the Gaussian fits were 0.022591 for Fig. 2e and 0.160624 for Fig. 2g, using MATLAB software. The relatively low P-value for Fig. 2e, at 0.022591, indeed suggests that the fit might not adequately describe the data, indicating a potential deviation from a Gaussian distribution. Recognizing the potential impact of sample size on the statistical analysis, we expanded our dataset for Fig. 2e from 30 to 100 data points (Fig. R14) and recalculated the K-S P-value for the Gaussian fit. The recalculated P-value for this larger dataset is 0.367545, significantly higher than the initial value. Typically, a threshold of 0.05 is used, where P-values greater than 0.05 indicate that the sample distribution does not significantly differ from the Gaussian distribution, supporting the hypothesis of a good fit.

This improvement suggests that with an increased sample size, the fit to a Gaussian distribution appears to be more appropriate for the data in Fig. R14, thereby addressing the initial concern regarding the randomness of the Si QD-related PL wavelength distribution.

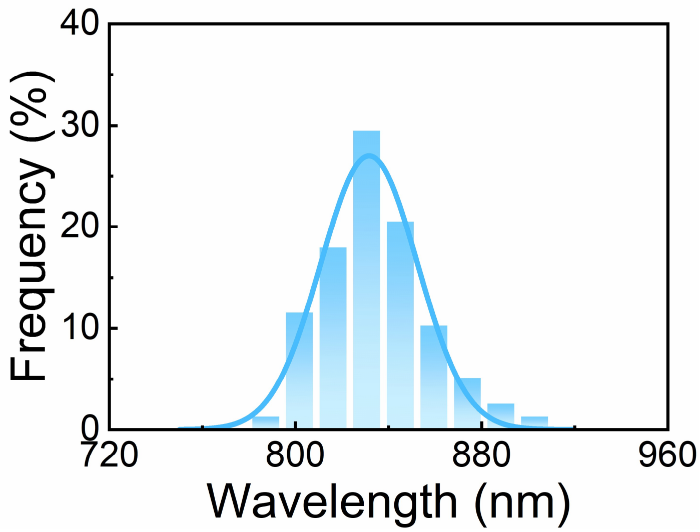

Fig. R14. Statistical distribution of the wavelength of the PL from Si QD.

Furthermore, we added Fig. R14 as Fig. 2e in the revised manuscript.

Comment 3.

Please give details about the following experiments in the Methods part or supplementary information.

(a). How the physical robustness of PUFs with/without PMMA was measured (Fig. S1c)? What is the concentration of the PMMA solution for spin coating?

Response to comment 3(a).

We thank the reviewer for pointing this out.

Measurement of Physical Robustness:

To evaluate the protective effect of PMMA on PUF devices, we conducted an experiment focusing on the variation of Si QD PL intensity as a proxy for physical robustness. This process involved the following steps:

(1) PUF Device Preparation: Two PUF devices were prepared, one encapsulated with PMMA and the other left unencapsulated.

(2) Immersion Test: Both devices were immersed in deionized water for varying durations (0, 4, 14, 21, 28, 42, and 49 days) to simulate the exposure to potentially damaging environments.

(3) Drying and Testing: After each immersion period, the surface water was carefully blown dried from the devices, and the Si QD PL intensity was measured at consistent locations on each device. To ensure accuracy in location across different testing times, metal markers were fabricated on the PUF surface (as detailed in response to comment 3(f)), allowing for precise repositioning for subsequent measurements. Each measurement was conducted five times at the same location on each device to ensure reliability.

PMMA Solution for Spin-coating:

The PMMA used for encapsulation was PMMA 950 A4, with a mass fraction of 4.1% dissolved in anisole. This specific concentration was chosen to achieve an optimal balance between protective coverage and maintaining the functional integrity of the underlying PUF structure.

Our revision to the manuscript:

(Lines 483-484, Page 18: in the revised main text):

PMMA (950 A4), featuring a 4.1% mass fraction in anisole, was acquired from Taizhou SUNANO New Energy Co., Ltd. (Taizhou, China).

(b). At least one set of images for key generations during statistical analysis should be shown in SI.

Response to comment 3(b).

We thank the reviewer for the suggestion. Fig. R15 illustrates the key generation process for all five optical responses, displaying representative images at each stage of the statistical analysis. This figure provides a clear, step-by-step depiction of the methodology, showcasing:

(1) Raw Data Acquisition (Fig. R15a): Original images captured for each of the five

optical responses, serving as the basis for further analysis.

(2) Gabor Filtering (Fig. R15b): Application of Gabor filtering to the raw images to enhance features and facilitate accurate thresholding for digitization.

(3) Digitization (Fig. R15c): Conversion of filtered images into binary format using a global thresholding method.

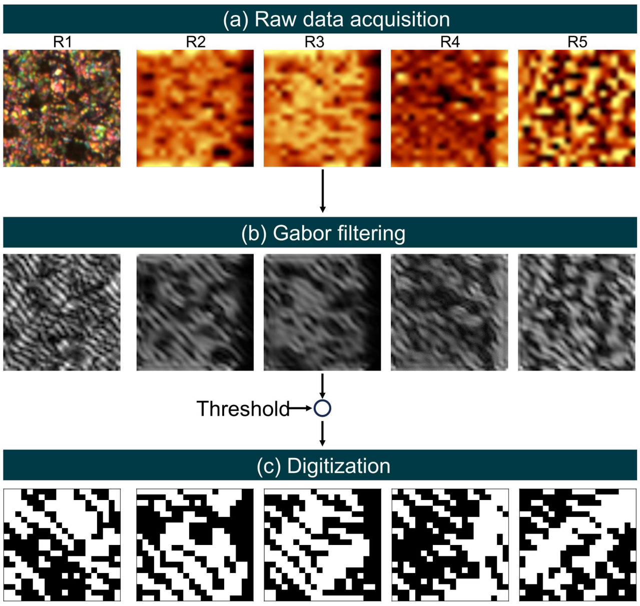

Fig. R15. Description of the bit extraction process, involving: (a) raw data acquisition, (b) Gabor filtering, and (c) digitization

Our revision to the manuscript:

(Lines 286-288, Page 11: in the revised main text):

A digitalization process is then performed, which compares with pre-established threshold values to generate response bits and the final cryptographic keys (Supplementary Fig. 7).

Furthermore, we added Fig. R15 as Supplementary Fig. 7 in the revised Supplementary Information.

(c). How many samples and which view (top or 30° side) are measured for the statistical analysis in Fig. 2gh? What are the following data processes?

Response to comment 3(c).

We thank the reviewer for pointing this out. To clarify, the analysis shown in Figs. 2g

and 2h is based on intrinsic material properties, such as PL intensity ratio (Fig. 2g) and lifetime (Fig. 2h), which do not involve angular measurements. Hence, the question might pertain to Figs. 1g and 1h instead.

Sample Details and the Measurement View:

For the statistical analysis illustrated in Figs. 1g and 1h, we conducted Scanning Electron Microscopy (SEM) imaging on five PUF devices. Each device was photographed from the top view, capturing the unique microstructural features characteristic of our PUFs. For each device, we acquired 10 SEM images, each with dimensions of 20 μm × 20 μm, resulting in a total of 50 SEM images subjected to statistical analysis.

Data Processing Steps:

The following data processing steps were employed to analyze the SEM images and extract the relevant microstructural metrics for statistical analysis:

(1) Pixel Length Determination: Using ImageJ software with the SEM scale bars, we calculated the actual length each pixel represented, ensuring precise dimensional analysis.

(2) Measurement of Inverted Pyramid Structures: We measured the longest side length and depth of the inverted pyramid structures seen in the SEM images, crucial for assessing structural variability across devices..

(3) Statistical Compilation: Measurements from the 50 SEM images provided 500 data points for statistical analysis, offering insight into the structural feature distribution.

(4) Distribution Graph Creation: We produced distribution graphs (Figs. 1g and 1h) to visually represent the structural features across our PUF devices.

Our revision to the manuscript:

(Lines 144-146, Page 5: in the revised main text):

For the statistical analysis presented in Figs. 1g and 1h, 50 top-view SEM images with each measuring the area of 20 μm × 20 μm are used.

(d). How was the simulation In lines 239-241 and Fig. S5d performed?

Response to comment 3(d).

We thank the reviewer for pointing this out. To explore the intricate interplay of light within the Si metasurface structure, we employed finite-difference time-domain (FDTD) simulations using Lumerical Solutions software. The detailed methodology of our simulation process is as follows:

1. Simulation Environment Setup: We initialized our simulation with the perfect matching layer (PML) boundary conditions applied in the x, y, and z directions. The PML conditions are critical for absorbing light in simulations, mimicking an infinitely extending space and thereby preventing any artificial reflections that could distort the results.

2. Source Configuration: The incident light was modeled as a total-field scattered-field (TFSF) plane wave with a wavelength of 830 nm. This approach allows for the separation of the incident and scattered fields, enabling us to analyze the interaction of the plane wave with the metasurface without interference from the boundaries of the simulation region.

3. Grid Layout: For the simulation domain, we utilized a precise grid layout with a resolution of 1 nm × 1 nm × 1 nm. Such a fine resolution ensures that the metasurface's features and the resulting electromagnetic fields are accurately captured, crucial for understanding the complex optical behaviors at play.

4. Field Monitoring: Electric field monitors were strategically placed within the simulation to capture the reflected field data. This step is pivotal as the reflected field, in our context, represents the emission characteristics of the dipole sources within the metasurface. By analyzing these fields, we can discern the far-field intensity distributions that emerge from the interaction between the incident plane wave and the metasurface structure.

By conducting these simulations, we aimed to gain a deeper understanding of how Si metasurface influences light propagation and distribution, elucidating the physical

principles that give rise to the observed complex optical patterns.

(e). How was the exciting field map generated?

Response to comment 3(e).

For generating the exciting field map, we employed a methodology similar to that for Fig. S5d (Please refer to the Response to Comment 3d of Reviewer 2 for more details.), with key differences highlighted below:

Light Source Wavelength: The incident plane wave's wavelength was adjusted to 405 nm, differing from the 830 nm used in Fig. S5d, to explore the interaction with Si metasurface at a different spectral point.

Field Monitoring: To capture the distribution of the exciting field, we placed field monitors on the surface of Si metasurface structure. These monitors are designed to record the electromagnetic field distribution as the incident light interacts with the metasurface, providing spatially resolved data on the field intensity.

Resulting Map: The data captured by the field monitors were then used to generate the exciting field map. This map visually represents the spatial distribution of the electromagnetic field across the metasurface structure when illuminated by the 405 nm light source, highlighting areas of enhanced field intensity due to the metasurface's unique properties.

Our revision to the manuscript:

(Lines 536-555, Page 20: in the revised main text):

In this article, we conducted FDTD simulations using the software "Lumerical Solutions". The Er-Si QDs were treated as electric dipole sources in this simulation, focusing on analyzing the Purcell factor and electric field distributions that include the reflected, exciting, and radiated fields. To accurately model the physical setup, we based our simulation on SEM images of the PUF surface. The simulation space was bounded by Perfectly Matched Layer (PML) conditions in all three dimensions (x, y, and z) to minimize any reflection artifacts that could skew the results. For the reflected field

analysis, we employed a total-field scattered-field (TFSF) source with a wavelength of 830 nm, representative of the emission characteristics of the dipole sources within the Er-Si QDs. Similarly, to simulate the exciting field, another 405 nm TFSF source was used to mimic the excitation conditions of the Er-Si QDs. Our simulation domain included a Si metasurface characterized by a random distribution, onto which we placed nine dipole sources at varying locations to simulate the distribution and emission of light from the Er-Si QDs. A highly detailed grid layout with 1 nm × 1 nm × 1 nm resolution was chosen to capture the intricate electric field variations near the metasurface and dipole sources. To extract the simulation results, we utilized electric field monitors and Purcell factor monitors strategically placed within the domain. These tools enabled us to capture the detailed electric field distributions (reflecting the reflected, excited, and radiated electric fields) and the Purcell factor enhancement at specific points on the Si metasurface.

(f). How were the metallic marks generated in Fig. S6?

Response to comment 3(f).

We thank the reviewer for pointing this out. The specific process for generating metallic markers is as follows:

(1) Mask Alignment: Initially, the mask is precisely positioned on the surface of the silicon wafer. The design and alignment of the mask ensure that, during the metal gold deposition, specific patterns are formed on the silicon wafer.

(2) First Layer Deposition: Following the mask's positioning, we proceed with the first deposition of metal gold. This step creates a metal gold layer on the silicon wafer surface, corresponding to the pattern of the mask.

(3) Mask Rotation and Re-positioning: After the first deposition, the mask is rotated clockwise by 90 degrees and precisely repositioned on the silicon wafer. This step is undertaken to create a new pattern on the silicon wafer that intersects with the pattern from the first deposition.

(4) Second Layer Deposition: With the mask repositioned, we conduct the second deposition of metal gold. This deposition creates a second layer on the silicon wafer, intersecting with the first layer's deposition, culminating in the final metallic marker pattern.

A schematic diagram of the overall preparation process is provided below:

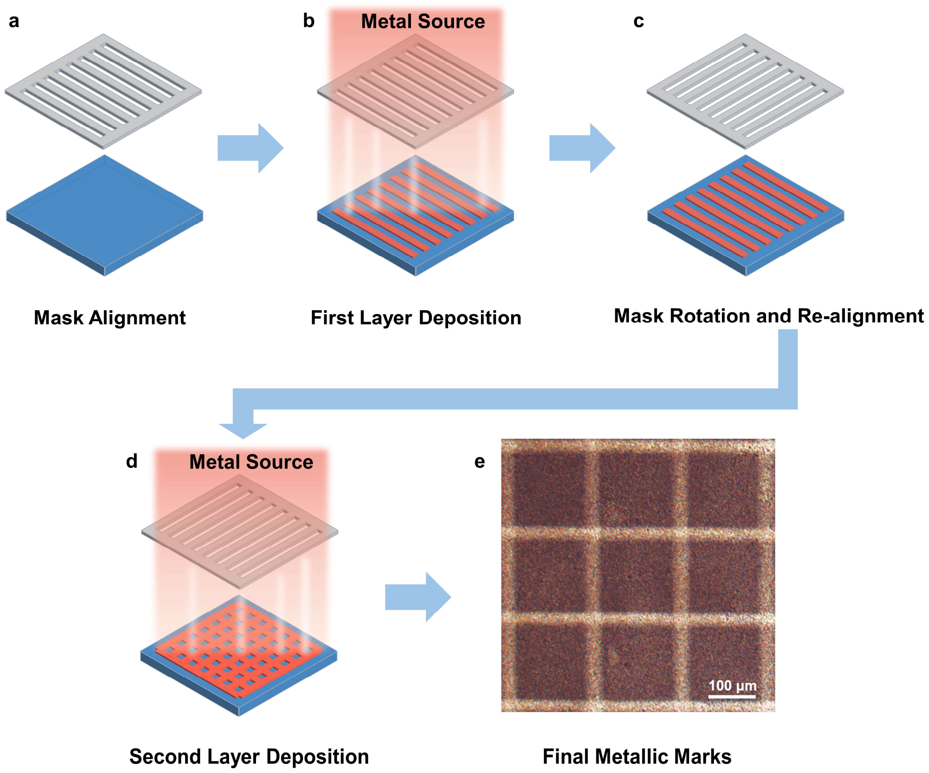

Fig. R16. Schematic diagram of the production process of metallic marks. Schematic diagram of the production process of metallic marks. a Mask alignment. **b** First layer deposition. **c** Mask rotation and re-positioning. The mask is rotated 90° clockwise. **d** Second layer deposition. e Final metallic marks.

Our revision to the manuscript:

(Lines 292-294, Page 11: in the revised main text):

Metallic marks (Supplementary Fig. 8) are used to simplify location tracking during recurrent measurements.

Furthermore, we added Fig. R16 as Supplementary Fig. 8 in the revised Supplementary Information.

Comment 4.

Minor comments:

(a). Full name needs to be given before the use of abbreviations. For example, PL.

Response to comment 4(a).

Thanks for highlighting this error. We have carefully reviewed our manuscript and revised it to include the full names of all terms prior to their abbreviation. For instance, we now introduce "photoluminescence (PL)" at its first mention, followed by the use of "PL" in subsequent references.

(b). The font in the figures needs to be uniform, including the SI figures.

Response to comment 4(b).

Thanks for pointing it out. In response to your comment, we have carefully reviewed all figures in our manuscript and SI to ensure that the font type, size, and style are uniform throughout. This includes the labels, annotations, and any text elements within the graphical representations.

(c). Line 161, 'on the other hand…', means 'but' not 'and'.

Response to comment 4(c).

Thanks for highlighting this error. We have revised the sentence to be " Additionally, Fig. 2d shows a position-dependent wavelength change of the PL associated with the band-to-band transition of Si QDs (R_4)" to more accurately convey our intended meaning.

(d). In SI, line 35, there is no d in Fig. 3. It should be corrected.

Response to comment 4(d).

Thanks for highlighting this error. The erroneous caption has been corrected in the Supplementary Information.

Reviewer #3:

The authors present all-silicon multimodal optical PUFs based on Er-doped Si QDs and Si metasurface. The fabrication process of the PUFs is compatible with CMOS technologies. Given the six in-situ optical responses of the PUFs, exceptionally high information entropy has been achieved. The performance of the PUFs is impressive in terms of bit uniformity, similarity, Intra-HD, Inter-HD, FAR, FRR, and encoding capacity. I think that the current work holds good promise for the future development of the anti-counterfeiting of ICs. The manuscript is overall OK. However, I should ask the authors to improve the manuscript by considering the following points.

Response:

We sincerely appreciate the feedback. Below, we have thoroughly addressed the reviewer's comments in detail.

Comment 1.

The authors in fact use the metasurface of a Si substrate. "Si metasurface" may be more appropriate than "Si metamaterial".

Response to comment 1.

We thank the reviewer for the valuable suggestion regarding the terminology of the key concept of the manuscript. We acknowledge that the term "Si metasurface" indeed provides a more precise description of our work than "Si metamaterial". We have changed "Si metamaterial" into "Si metasurface" through the whole revised manuscript.

Comment 2.

Do the authors refer to the length of the longest side of an inverted pyramid by saying "the length of maximum side"? For Fig. 1g it seems that the range of the length is not important. What matters is its average.

Response to comment 2.

We thank the reviewer for the insightful comment. "the length of maximum side," indeed means the length of the longest side of an inverted pyramid structure. We should apologize that our initial manuscript might have unintentionally suggested that the

range of these lengths is of importance. Moreover, the reviewer's observation rightly points out that for the analysis and interpretation of our results, the average length of these sides holds more significance, particularly when considering the modulation of light emission from Er-Si QDs through the Si metasurface.

We have clarified this point in our revised manuscript by adjusting our language to emphasize that it is the average length of the sides of the inverted pyramids that is most relevant to our study.

Our revision to the manuscript:

(Lines 152-153, Page 6: in the revised main text):

These results indicate that the longest side and the depth of an inverted pyramid are ~ 610 nm and 660 nm, respectively.

Comment 3.

What is the full name of OR?

Response to comment 3.

Thanks for pointing it out. We apologize for any confusion caused by unclear expressions in our manuscript. Upon reviewing our manuscript and supplementary information, we could not locate the reference to "OR" that you mentioned. We speculate that there might have been a typographical error, and you could be referring to "QR". To clarify, <u>the full name of "QR" is "Quick Response".</u>

Furthermore, in the revised manuscript, we have ensured that every abbreviation is clearly defined upon its first appearance, followed by the abbreviation in parentheses.

Our revision to the manuscript:

(Lines 211-212, Page 8: in the revised main text):

For example, R_4 of three PUFs is transformed into a binary barcode or quick-response (QR) code by introducing a predefined threshold (Fig. 2i, Supplementary Note 3).

Comment 4.

PL peaks at 1540 nm in Fig. 2d are too small to be clearly seen. Please provide a detailed caption for Fig. 2f.

Response to comment 4.

Thanks for pointing out the issue. To address this, we have magnified the PL peaks at 1540 nm by a factor of five (Fig. R17), ensuring that the details of the PL peaks are clearly visible.

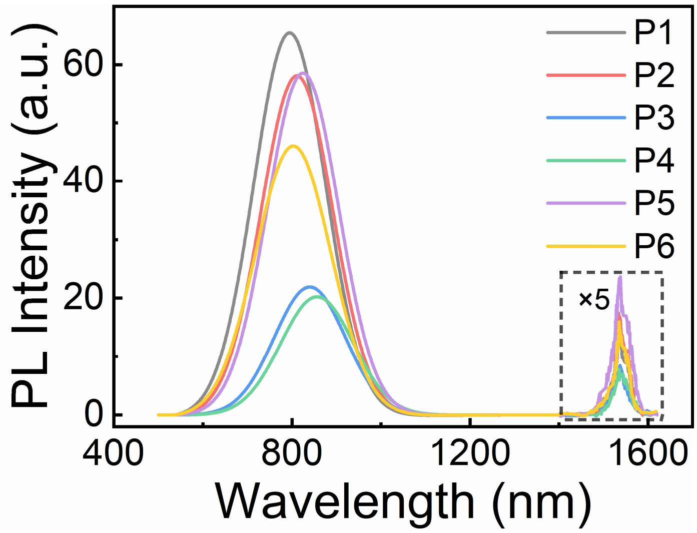

Fig. R17. PL spectra of the PUF on different positions. The PL peaks at 1540 nm are magnified by a factor of five.

Regarding the detailed caption for Fig. 2f, we appreciate your request for clarity. Our revision to the manuscript:

(Lines 188-190, Page 7: in the revised main text):

Fig. 2f: Demonstration of the random PL wavelength of Si QDs by using 20 positions per sample. $\lambda_{average}$ is the average value of the PL wavelength. ($\lambda - \lambda_{average}$) quantifies the variation of the PL wavelength.

Furthermore, we added Fig. R17 as Fig. 2d in the revised manuscript.

Comment 5.

Please clearly tell if a PUF is a 40 × 40 μm^2 square. Does an image correspond to a PUF in Fig. 4 c&d?

Response to comment 5.

We thank the reviewer's comment. To clarify, each PUF is indeed designed as a 40 × 40 μm^2 square. This specific dimensionality is chosen to optimize the balance between the physical space required for embedding sufficient unique features and the practicality of imaging and analysis. Furthermore, in Fig. 4 c&d, each image indeed corresponds to a single PUF.

We have revised our manuscript as follows (Lines 291-292, Page 11: in the revised main text):

Each PUF is designed to be represented by a square image with the size of 40 × 40 μm^2.

Comment 6.

More key information needs to be moved from Supplementary Note 3 to the main text.

Response to comment 6.

Thanks for pointing out the issue. We have included an essential description of our method for correcting image discrepancies caused by device vibrations and other factors. This additional content enhances the main text as follows (Lines 294-305, Page 11: in the revised main text):

Prior to analysis, the discrepancies in the image arising from device vibrations and other factors are corrected by utilizing the principle of phase correlation (Supplementary Note 4)[65,66]. The discrepancies are manifested in the frequency domain as follows:

$$F(u,v) = G(u,v)\exp\left[2\pi i\left(\frac{udx}{M}+\frac{vdy}{N}\right)\right] \quad (R4)$$

where $F(u,v)$ represents the Fourier transform of the reference image, $G(u,v)$ denotes the Fourier transform of the images requiring correction. The term $\exp\left[2\pi i\left(\frac{udx}{M}+\frac{vdy}{N}\right)\right]$ represents the correction factor, where dx and dy are the displacements in the x and y directions, respectively, and M and N are the dimensions of the image in the x and y directions, respectively. Once the correction is accomplished, the speeded-up robust features (SURF) algorithm[67] is employed to verify the location of identical feature regions within the image.

Comment 7.

The authors may show data to prove that the light emission from Er^{3+} in Er-Si QDs is environmentally stable (independent of position) in the Supplementary Information.

Response to comment 7.

We thank the reviewer for this valuable comment. To address this, we conducted comprehensive tests on the photoluminescence (PL) spectra (Fig. R18a) and time-resolved spectroscopy (Fig. R18b) of Er^{3+} across five distinct locations within a PUF, including the center and edges.

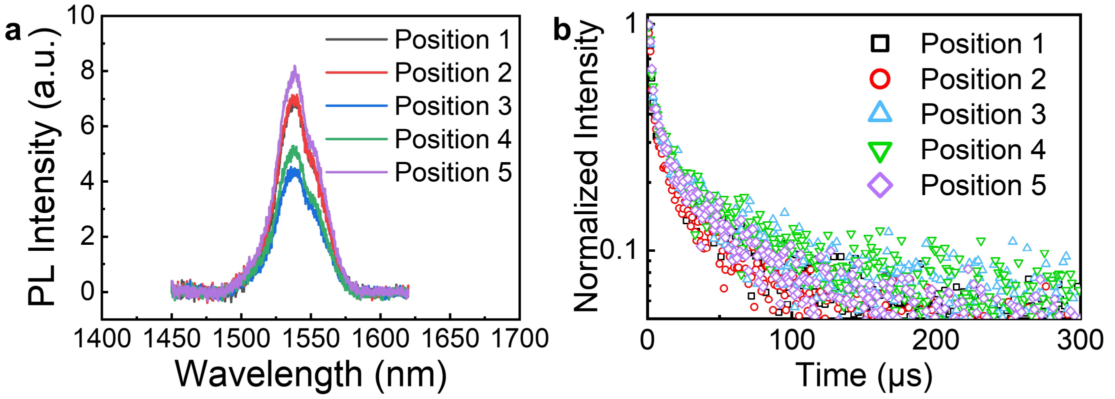

Fig. R18. a Er^{3+} PL spectra of the PUF on different positions. b The PL lifetime of Er^{3+} at different positions.

Our findings indicate variability in the PL intensity of Er^{3+} across different positions, likely attributable to the inhomogeneity in the drop-casting process and the irregularities of the silicon metasurface. However, it is crucial to note that despite these intensity variations, the PL wavelength and lifetime of Er^{3+} remained consistent across all tested locations. This consistency in wavelength and lifetime under various environmental conditions underscores the inherent stability of Er^{3+} light emission characteristics within Er-Si QDs.

These results have been detailed and included in the Supplementary Information section of our manuscript. This inclusion provides clear evidence that, although PL intensity may vary due to physical deposition and surface irregularities, the fundamental

properties of Er^{3+} emission—namely, the wavelength and lifetime—are robust and environmentally stable.

Our revision to the manuscript:

(Lines 219-226, Page 8: in the revised main text):

To figure out the coupling mechanism between the Si metasurface and Er-Si QDs, FDTD simulations are employed. These Er-Si QDs contain two emitters: Er^{3+} and Si QDs. Owing to the shielding of the 4*f* shell orbital by the outer 5*s* and 5*p* orbitals for Er^{3+} (ref. [58]), the PL wavelength and lifetime of Er^{3+} in Er-Si QDs exhibit rather weak sensitivity to environmental changes (Supplementary Fig. 5), limiting their use for anti-counterfeiting. Thus, the following FDTD simulations focus on the optical responses related to the band-to-band transitions of Er-Si QDs (i.e., the PL emission of Si QDs), particularly in relation to the interaction with the Si metasurface.

Furthermore, we added Fig. R18 as Supplementary Fig. 5 in the revised Supplementary Information.

Comment 8.

The English may be improved throughout the manuscript.

Response to comment 8.

We thank the reviewer for this feedback regarding the English language quality of our manuscript. We have tried our best to improve the manuscript and made cosmetic changes to the manuscript. These changes will not influence the content and framework of the paper. And here we did not list the changes but marked in red in the revised manuscript. We appreciate for Reviewer's warm work earnestly and hope that the correction will meet with approval.

Reviewer #4:

The authors demonstrated a type of all-silicon-based anti-counterfeiting labels with multimodal optical physical unclonable functions (PUFs). The preparation of the PUF label is compatible with CMOS processes. The PUF pixels consist of Si metamaterial structure and Er^{3+} doped Si quantum dots (QDs). Different optical responses, including the micropattern imaging, Si QD PL intensity, Er^{3+} PL intensity, Si QD PL wavelength, Si QD PL lifetime, can be employed for multimode encoding, enabling the PUF labels with a high information entropy and encoding capacity. The research is interesting. The large-scale fabrication of silicon-based multimodal optical PUF labels should be very beneficial to the integrated circuit anti-counterfeiting and can be applied in other anti-counterfeiting areas. The work is of high novelty, but the current manuscript still needs to be improved before it can be published.

Response:

We gratefully thank the reviewer's time, helpful comments, and appreciation of the novelty of our work. We have thoroughly addressed the reviewer's concerns in our following point-by-point response.

Comment 1.

The term "ratiometric PL intensity (R_5)" is not clearly defined when it first appeared in the main text. It is the intensity ratio between the Si QD PL intensity (R_2) and the Er^{3+} PL intensity (R_3). R_5 therefore is not an independent channel for encryption. The calculation of the encoding capacity should be corrected.

Response to comment 1.

We thank the reviewer for the insightful comment. Ratiometric PL intensity indeed refers to the intensity ratio of Si QD PL intensity (R_2) to the Er^{3+} PL intensity (R_3). We acknowledge your correct assessment that ratiometric PL intensity, being derived from R_2 and R_3, does not constitute an independent channel for encryption. Furthermore, we have corrected the calculation of the encoding capacity in our manuscript as follows (Lines 385-387, Page 15: in the revised main text):

The PUF in our study possesses a *c* value of 5, consisting of five optical responses.

Accordingly, we image an area of 20 × 20 pixels, providing a theoretical encoding capacity of 5^{400} (i.e., 3.87×10^{279}).

Comment 2.

The security key employing PL lifetime requires the instrumentation of microsecond lifetime measurement, which are usually not easy to access. Could the author comment on how to make use of such channel in regular applications?

Response to comment 2.

Thanks for highlighting the concern regarding the accessibility of microsecond lifetime measurement. Indeed, the necessity for specialized equipment has historically been a limitation for the widespread adoption of PL lifetime measurements in conventional applications. However, recent developments in fluorescence lifetime imaging microscopy (FLIM) techniques, such as those based on pulse sampling[8,9] and frequency domain[10,11] methods, now facilitate the rapid acquisition of fluorescence lifetimes at the microsecond level. Additionally, innovative analytical methods like phasor analysis[12,13] and Laguerre deconvolution[14,15] offer fit-free alternatives for analyzing fluorescence lifetimes, making the process more accessible and user-friendly.

In conclusion, while the requirement for specialized instruments for microsecond lifetime measurements has previously posed challenges, the advent of new FLIM technologies and analytical techniques is significantly reducing these obstacles. We remain optimistic about the practical application of PL lifetime as a reliable security feature and are committed to furthering research and development in this area.

Our revision to the manuscript:

(Lines 374-378, Page 14: in the revised main text):

With the progress of portable spectrometer technology, the ongoing improvements of smartphone cameras, and the evolution of fluorescence imaging techniques based on pulse sampling[70,71] and frequency domain[72,73], we anticipate that the potential of extracting all the five optical responses by using even more affordable and user-friendly

devices may be realized.

Comment 3.

It is true that the light emission from Er^{3+} is environmentally stable. But the emission will be modulated by the optical density of states in the environment. The interplay between Si metamaterial and emission from Er^{3+} actually should be considered. For example, it has been reported that the emission from Er^{3+} can be modulated by the plasmon resonances of metal nanostructures (ACS Nano 2023, 17, 11, 10546–10559).

Response to comment 3.

We thank the reviewer for this insightful comment regarding the interaction between Er^{3+} emissions and the Si metasurface. Your reference to the modulation of lanthanide ion emissions by plasmon resonances in metal nanostructures[39] underscores a significant aspect of photonic research that merits careful consideration in our work.

We apologize that our previous descriptions might have led to some confusion. To clarify, while the PL wavelength and lifetime of Er^{3+} are environmentally stable and exhibit minimal variation with changing conditions, the PL intensity can vary depending on the surrounding environment (Details can be seen in Reviewer 3 Comment 7). To explore this further, we conducted Er^{3+} PL intensity mapping tests on devices featuring both Si metasurface and bare Si substrate to study the influence of Si metasurface on the distribution of Er^{3+} PL intensity. Our findings indicate that PL intensity mapping images from the Si metasurface devices possess greater randomness and chaos compared to those from the bare Si substrate (Details can be seen in Reviewer 2 Comment 2f), demonstrating the interplay between Si metasurface and emission from Er^{3+}. Acknowledging this, we have revised our manuscript to better highlight the impact of the Si metasurface on the PL intensity of Er^{3+}.

Our revision to the manuscript:

(Lines 174-176, Page 6: in the revised main text):

In particular, the greater randomness of R_3 derived from the Si metasurface compared

to those from the bare Si substrate (Supplementary Fig. 3) well indicates the interplay between the Si metasurface and R_3.

(Lines 219-226, Page 8: in the revised main text):

To figure out the coupling mechanism between the Si metasurface and Er-Si QDs, FDTD simulations are employed. These Er-Si QDs contain two emitters: Er^{3+} and Si QDs. Owing to the shielding of the 4*f* shell orbital by the outer 5*s* and 5*p* orbitals for Er^{3+} (ref. [58]), the PL wavelength and lifetime of Er^{3+} in Er-Si QDs exhibit rather weak sensitivity to environmental changes (Supplementary Fig. 5), limiting their use for anti-counterfeiting. Thus, the following FDTD simulations focus on the optical responses related to the band-to-band transitions of Er-Si QDs (i.e., the PL emission of Si QDs), particularly in relation to the interaction with the Si metasurface.

Comment 4.

The Si structures should not support a localized surface plasmon at the wavelength of 405 nm. The authors are suggested to correct their statement (see line 243-246).

Response to comment 4.

We thank the reviewer for the astute observation regarding our discussion on the behavior of Si metasurface structures at a wavelength of 405 nm. Upon revisiting our manuscript, we recognize that our initial statement might have inadvertently suggested that silicon metasurface support localized surface plasmon resonances (LSPRs) in a manner similar to metallic systems. Given silicon's dielectric nature and its lack of free electron density typical of metals, this comparison may have been misleading.

The core of our investigation pertains to the enhancement of electromagnetic fields around Si metasurface structures. This enhancement, initially attributed to LSPRs, is, in fact, more accurately ascribed to diffractive coupling[40] and the interaction with photonic modes[41–43] facilitated by Si metasurface's unique structure.

In response to your valuable feedback, we have revised the statement (Lines 269-272,

Page 8: in the revised main text):

Under the illumination at the wavelength of 405 nm, the metasurface exhibits tapestry-like hotspots of the local exciting field (Supplementary Fig. 6e) owing to the diffractive coupling[42] and the interaction with photonic modes[61–63].

Comment 5.

The calculation of the information entropy seems wrong (please also refer to comment (1)).

Response to comment 5.

Thanks for highlighting the issue with our information entropy calculation. We refined our approach to accurately assess information entropy using only independent signals: micropattern imaging (R_1), Si QD PL intensity (R_2), Er^{3+} PL intensity (R_3), Si QD PL wavelength (R_4), and Si QD PL lifetime (R5). The formula used for calculating information entropy is as follows:

$$Information\ entropy = -\sum_{i=1}^{n} P(x_i)\log_2[P(x_i)] \qquad (R5)$$

With the corrected approach, considering five independent signals, our revised calculation of information entropy yields **2.32 bits/pixel**.

Our revision to the manuscript:

(Lines 207-209, Page 8: in the revised main text):

Hence, an ultrahigh information entropy (up to 2.32 bits/pixel) is obtained (Supplementary Note 2), which is superior to those of PUFs based on alternative technologies[7,8,10,13,21,32–35,53–57] (Supplementary Fig. 4).

Comment 6.

Could the authors estimate the cost of the PUF label?

Response to comment 6.

Thanks for the comment. The total cost for fabricating one piece of 1.5 × 1.5 cm^2 sized PUF label is **1.45 USD**. Further detailed analysis indicates that the cost for producing each micro-sized PUF label (40 μm × 40 μm) is remarkably low, approximately **10^{-5}**

USD. The detailed analysis has been given in the Response to Comment 1 of Reviewer 1.

Our revision to the manuscript:

(Lines 123-126, Page 4: in the revised main text):

Moreover, cost analysis reveals that the total cost for the fabrication of each PUF label is approximately ~ 10^{-5} USD, highlighting the economic viability of the current approach for widespread applications (Supplementary Table 1 and Supplementary Note 1).

References

R1. Chia, X. X. & Tan, D. T. H. Deuterated SiNx: A low-loss, back-end CMOS-compatible platform for nonlinear integrated optics. *Nanophotonics* **12**, 1613–1631 (2023).

R2. Marchack, N. *et al.* Plasma processing for advanced microelectronics beyond CMOS. *J. Appl. Phys.* **130**, 080901 (2021).

R3. Quirk, M. & Julian, S. *Semiconductor manufacturing technology*. vol. 1 (Pearson, 2001).

R4. Liao, Y., Shin, S. H., Jin, Y., Wang, Q. J. & Kim, M. Producing Microscale Ge Textures via Titanium Nitride- and Nickel-Assisted Chemical Etching with CMOS-Compatibility. *Adv. Mater. Interfaces* **8**, 1–8 (2021).

R5. Cui, J. *et al.* CMOS-compatible electrochemical synaptic transistor arrays for deep learning accelerators. *Nat. Electron.* **6**, 292–300 (2023).

R6. Conti, S. *et al.* Printed transistors made of 2D material-based inks. *Nat. Rev. Mater.* **8**, 651–667 (2023).

R7. He, Z. *et al.* CMOS Compatible High-Performance Nanolasing Based on Perovskite–SiN Hybrid Integration. *Adv. Opt. Mater.* **8**, 1–8 (2020).

R8. Gorpas, D. *et al.* Autofluorescence lifetime augmented reality as a means for real-time robotic surgery guidance in human patients. *Sci. Rep.* **9**, 1–9 (2019).

R9. Alfonso-Garcia, A. *et al.* Real-time augmented reality for delineation of surgical margins during neurosurgery using autofluorescence lifetime contrast. *J. Biophotonics* **13**, 1–12 (2020).

R10. Liu, X. *et al.* Fast fluorescence lifetime imaging techniques: A review on challenge and development. *J. Innov. Opt. Health Sci.* **12**, 1–27 (2019).

R11. Datta, R., Heaster, T. M., Sharick, J. T., Gillette, A. A. & Skala, M. C. Fluorescence lifetime imaging microscopy: fundamentals and advances in instrumentation, analysis, and applications. *J. Biomed. Opt.* **25**, 1 (2020).

R12. Rowley, M. I., Barber, P. R., Coolen, A. C. C. & Vojnovic, B. Bayesian analysis of fluorescence lifetime imaging data. *Multiphot. Microsc. Biomed. Sci. XI* **7903**, 790325 (2011).

R13. Ranjit, S., Malacrida, L., Jameson, D. M. & Gratton, E. Fit-free analysis of fluorescence lifetime imaging data using the phasor approach. *Nat. Protoc.* **13**, 1979–2004 (2018).

R14. Fereidouni, F., Gorpas, D., Ma, D., Fatakdawala, H. & Marcu, L. Rapid fluorescence lifetime estimation with modified phasor approach and Laguerre deconvolution: a comparative study. *Methods Appl. Fluoresc.* **5**, 035003 (2017).

R15. Pande, P., Trivedi, C. A. & Jo, J. A. Analysis of fluorescence lifetime imaging microscopy (FLIM) data: Based on a fully automated laguerre deconvolution method. *Methods Inf. Med.* **49**, 531–536 (2010).

R16. Caligiuri, V. *et al.* Hybrid Plasmonic/Photonic Nanoscale Strategy for Multilevel Anticounterfeit Labels. *ACS Appl. Mater. Interfaces* **13**, 49172–49183 (2021).

R17. Jiang, S., Kim, S. H., Park, C. S., Lee, W. Bin & Lee, S. S. Multilevel Anti-Counterfeiting Based on Covert Structural Features Embedded in Femtosecond-Laser-Treated Gold Nanocluster/Graphene Hybrid Layer. *ACS Appl. Mater. Interfaces* **14**, 39240–39248 (2022).

R18. Sun, N., Chen, Z., Wang, Y. & Wang, S. Random fractal-enabled physical unclonable functions with dynamic AI authentication. *Nat. Commun.* **14**, 2185 (2022).

R19. Kim, J. H. *et al.* Nanoscale physical unclonable function labels based on block co-polymer self-assembly. *Nat. Electron.* **5**, 433–442 (2022).

R20. Zhang, J. *et al.* An all-in-one nanoprinting approach for the synthesis of a nanofilm library for unclonable anti-counterfeiting applications. *Nat. Nanotechnol.* **18**, 1027–1035 (2023).

R21. Zhang, L., Fong, X., Chang, C.-H., Kong, Z. H. & Roy, K. Highly reliable memory-based physical unclonable function using spin-transfer torque MRAM. in *2014 IEEE International Symposium on Circuits and Systems (ISCAS)* 2169–2172 (IEEE, 2014). doi:10.1109/ISCAS.2014.6865598.

R22. Dodda, A. *et al.* Graphene-based physically unclonable functions that are reconfigurable and resilient to machine learning attacks. *Nat. Electron.* **4**, 364–374 (2021).

R23. Alharbi, A., Armstrong, D., Alharbi, S. & Shahrjerdi, D. Physically unclonable cryptographic primitives by chemical vapor deposition of layered MoS_2. *ACS Nano* **11**, 12772–12779 (2017).

R24. Liu, R., Wu, H., Pang, Y., Qian, H. & Yu, S. Experimental characterization of physical unclonable function based on 1 kb resistive. *IEEE Electron Device Lett.* **36**, 1380–1383 (2015).

R25. Zhang, R. *et al.* Nanoscale diffusive memristor crossbars as physical unclonable functions. *Nanoscale* **10**, 2721–2726 (2018).

R26. Kim, K. *et al.* Voxelated opto-physically unclonable functions via irreplicable wrinkles. *Light Sci. Appl.* **12**, 1–13 (2023).

R27. Lee, S. *et al.* Spintronic Physical Unclonable Functions Based on Field-Free Spin–Orbit-Torque Switching. *Adv. Mater.* **34**, 2203558 (2022).

R28. Kim, D. *et al.* Reconfigurable Electronic Physically Unclonable Functions Based on Organic Thin-Film Transistors with Multiscale Polycrystalline Entropy for Highly Secure Cryptography Primitives. *Adv. Funct. Mater.* **33**, 2210367 (2023).

R29. Hu, Z. *et al.* Physically unclonable cryptographic primitives using self-Assembled carbon nanotubes. *Nat. Nanotechnol.* **11**, 559–565 (2016).

R30. Zhong, D. *et al.* Twin physically unclonable functions based on aligned carbon nanotube arrays. *Nat. Electron.* **5**, 424–432 (2022).

R31. Zhang, T. *et al.* Multimodal dynamic and unclonable anti- counterfeiting using robust diamond microparticles on heterogeneous substrate. *Nat. Commun.* **14**, 2507 (2023).

R32. Guo, H. *et al.* Multilevel Encoding Physically Unclonable Functions Based on The Multispecies Structure in Diamonds. *Adv. Funct. Mater.* **2304648**, 1–10 (2023).

R33. Gu, Y. *et al.* Gap-enhanced Raman tags for physically unclonable anticounterfeiting labels. *Nat. Commun.* **11**, 516 (2020).

R34. Wang, F. H., Yao, X. H. & Han, J. Q. Iris recognition based on multichannel Gabor filtering and feature fusion. *Guangdian Gongcheng/Opto-Electronic Eng.* **34**, 72–76 (2007).

R35. Chen, F. *et al.* Unclonable fluorescence behaviors of perovskite quantum dots/chaotic metasurfaces hybrid nanostructures for versatile security primitive. *Chem. Eng. J.* **411**, 128350 (2021).

R36. Chen, D. J., Chiang, F. P., Tan, Y. S. & Don, H. S. Digital speckle-displacement measurement using a complex spectrum method. *Appl. Opt.* **32**, 1839 (1993).

R37. Ge, H. *et al.* Digital image frequency spectrum method for analyzing speckle displacement in frequency domain. *Opt. Lett.* **40**, 942 (2015).

R38. Bay, H., Ess, A., Tuytelaars, T. & Van Gool, L. Speeded-up robust features (SURF). *Comput. Vis. Image Underst.* **110**, 346–359 (2008).

R39. Zhang, J., Cheng, X., Zhang, H., Zheng, J. & Wang, J. Plasmon-Controlled Selective Emission Enhancement of Eu^{3+} with (Au Core)@(Y(V,P)O 4 :Eu) Nanostructures. *ACS Nano* **17**, 10546–10559 (2023).

R40. Staude, I. & Schilling, J. Metamaterial-inspired silicon nanophotonics. *Nat. Photonics* **11**, 274–284 (2017).

R41. Shcherbakov, M. R. *et al.* Enhanced third-harmonic generation in silicon nanoparticles driven by magnetic response. *Nano Lett.* **14**, 6488–6492 (2014).

R42. Yang, Y. *et al.* Nonlinear Fano-Resonant Dielectric Metasurfaces. *Nano Lett.* **15**, 7388–7393 (2015).

R43. Cui, C. *et al.* Light emission driven by magnetic and electric toroidal dipole resonances in a silicon metasurface. *Nanoscale* **11**, 14446–14454 (2019).

REVIEWERS' COMMENTS

Reviewer #1 (Remarks to the Author):

The authors have addressed my concerns with clear analysis and sufficient evidence. I recommend this work for publication in Nature Communications.

Reviewer #2 (Remarks to the Author):

The comments were well addressed. Acceptance for publication is recommended.

Reviewer #3 (Remarks to the Author):

The authors have addressed the reviewers' comments properly. And I would like to suggest acceptance at its current status.

Reviewer #4 (Remarks to the Author):

The authors have fully addressed all questions, comments, and suggested changes from the first round of review in a very careful and thorough way. The quality of the manuscript has been improved remarkably. I strongly suggest the publication of this work without any further changes.

POINT-BY-POINT RESPONSE TO REVIEWER COMMENTS

We express our gratitude to all the reviewers for their insightful feedback, which has been instrumental in refining our work. Please see below our detailed response to the comments, with the response in blue and the modifications in the revision in red.

REVIEWER COMMENTS

Reviewer #1:

The authors have addressed my concerns with clear analysis and sufficient evidence. I recommend this work for publication in Nature Communications.

Response:

We sincerely thank the reviewer for the positive evaluation and recommendation for publication in Nature Communications. The reviewer's insightful comments and feedback have been invaluable in improving the quality and clarity of our work.

Reviewer #2:

The comments were well addressed. Acceptance for publication is recommended.

Response:

We thank the reviewer for the positive feedback and recommendation for acceptance of our manuscript. We greatly appreciate the time and effort the reviewer has dedicated to reviewing our work thoroughly.

Reviewer #3:

The authors have addressed the reviewers' comments properly. And I would like to suggest acceptance at its current status.

Response:

We thank the reviewer for the positive recommendation. We appreciate the time and expertise the reviewer has dedicated to evaluating our manuscript thoroughly.

Reviewer #4:

The authors have fully addressed all questions, comments, and suggested changes from the first round of review in a very careful and thorough way. The quality of the manuscript has been improved remarkably. I strongly suggest the publication of this work without any further changes.

Response:

We are deeply appreciative of the reviewer's positive evaluation and strong recommendation for the publication of our manuscript. We extend our sincere gratitude once more for dedicating valuable time and effort, as well as providing invaluable insights that have significantly enhanced the quality of our work.

Other minor modifications

1. We optimized the images used in all figures to increase clarity and avoid potential copyright ambiguity.

   Our revision to the manuscript:

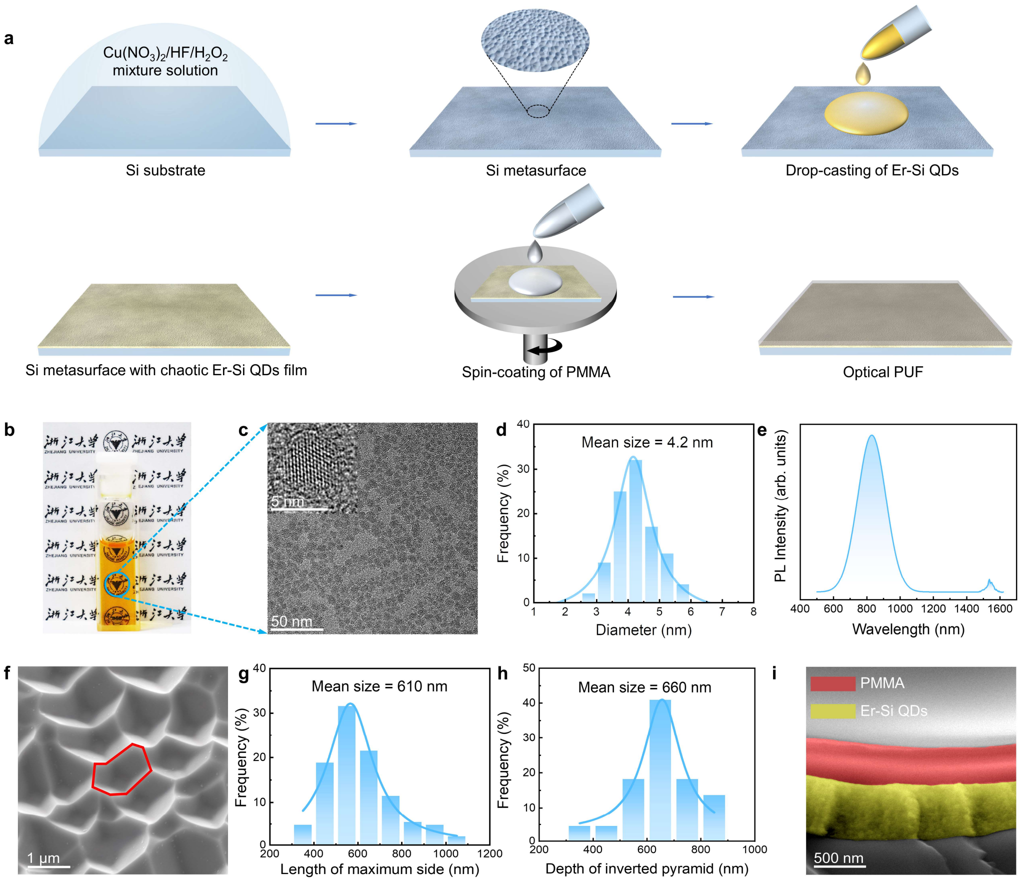

Fig. 1 | Preparation and structural characterization of an all-Si multidimensionally-encoded optical PUF. a Schematic representation of the fabrication process of an all-Si multidimensionally-encoded optical PUF. **b** Photograph of a solution of Er-Si QDs. **c** Transmission electron microscopy (TEM) image of Er-Si QDs. The inset displays a high-resolution TEM image. **d** Size distribution of Er-Si QDs. **e** PL spectrum of Er-Si QDs. **f** Scanning electron microscopy (SEM) image at a 30° side view of the random array of inverted pyramids. The red lines depict the sides of an inverted pyramid. **g** Distribution of the maximum length of the sides of an inverted pyramid. **h** Distribution of the depth of an inverted pyramid. For the statistical analysis presented in Figs. 1g and 1h, 50 top-view SEM images with each measuring the area of 20 μm × 20 μm are used. **i** Cross-sectional SEM image of an all-Si multidimensionally-encoded optical PUF.

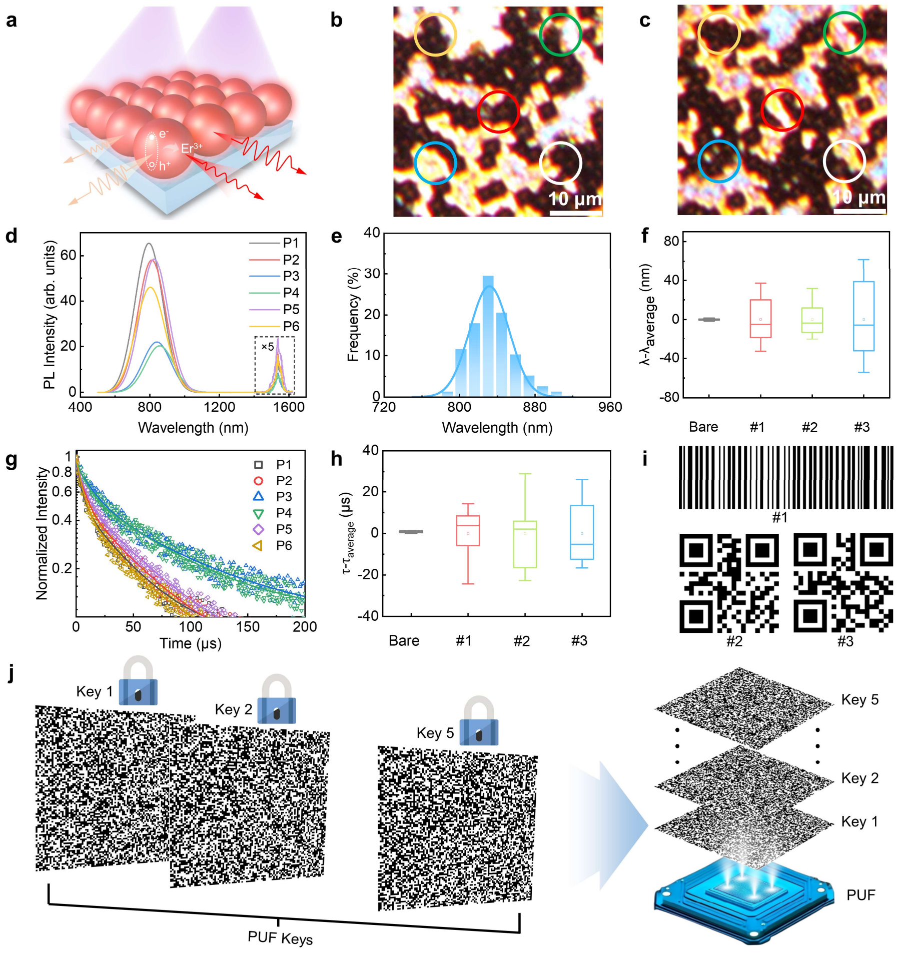

Fig. 2 | All-Si multidimensionally-encoded optical PUF encryption features. a Illustration depicting the working principle of the PUF. Top-view optical microscopy images of a PUF at positions 1 (**b**) and 2 (**c**). The overlaid circles highlight differences between the random micropatterns. **d** PL spectra of the PUF at six different positions. The PL peaks at 1540 nm are magnified by a factor of five. **e** Statistical distribution of the wavelength of the PL from Si QD. **f** Demonstration of the random PL wavelength of Si QDs by using 20 positions per sample. $\lambda_{average}$ is the average value of the PL wavelength. ($\lambda - \lambda_{average}$) quantifies the variation of the PL wavelength. **g** The PL lifetime of Si QDs at six different positions. **h** Demonstration of the random PL lifetime of Si QDs by using 20 positions per sample. $\tau_{average}$ is the average value of the PL lifetime. ($\tau - \tau_{average}$) quantifies the variation of the PL lifetime. **i** Optical key generation from various samples with distinct PL-wavelength thresholds. **j** Schematic image of five security keys extracted from a single PUF. All boxplots illustrate the interquartile range (IQR), extending from the first to the third quartile, with a central line denoting the median. Whiskers extend from the quartiles to the minimum and maximum data points. Hollow square points indicate the mean values.

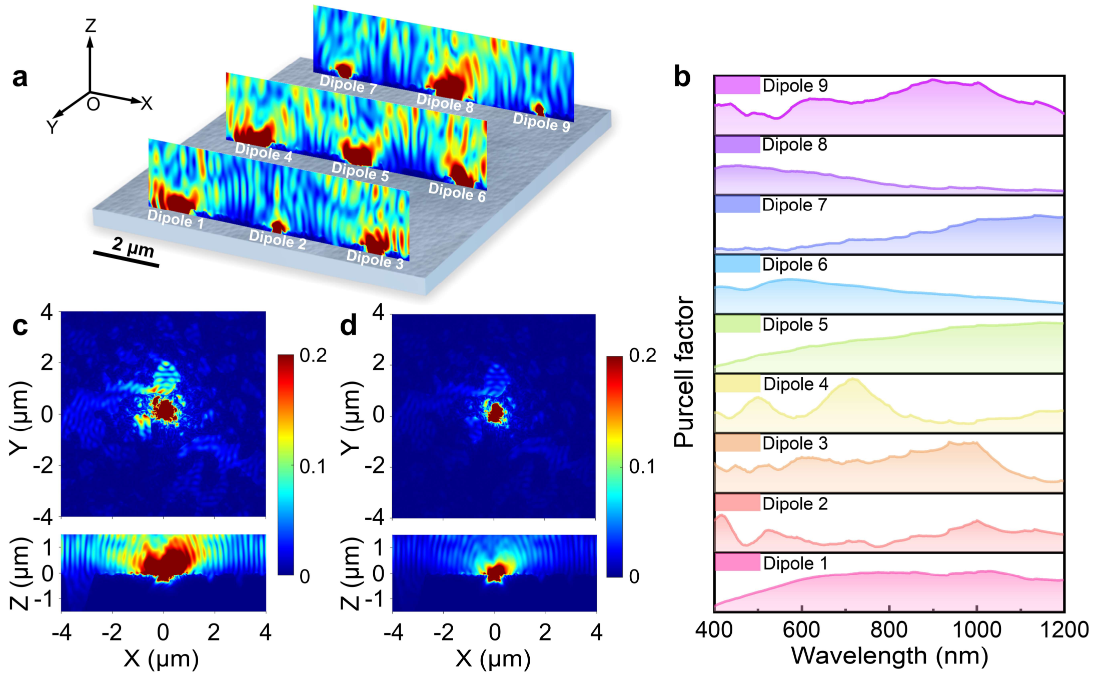

Fig. 3 | Simulated position-dependent optical behaviors in an all-Si multidimensionally-encoded optical PUF. a Radiation field distribution at the wavelength of 830 nm for nine dipole emitters across the Si metasurface. **b** Comparison of Purcell factors for the nine dipole locations. **c** Radiation field intensity distributions in the XOY plane (top) and XOZ plane (bottom) for a single dipole at its original position of the Si metasurface. **d** Radiation field intensity distributions in the XOY plane (top) and XOZ plane (bottom) for the same dipole shifted 10 nm from its original position at the Si metasurface.

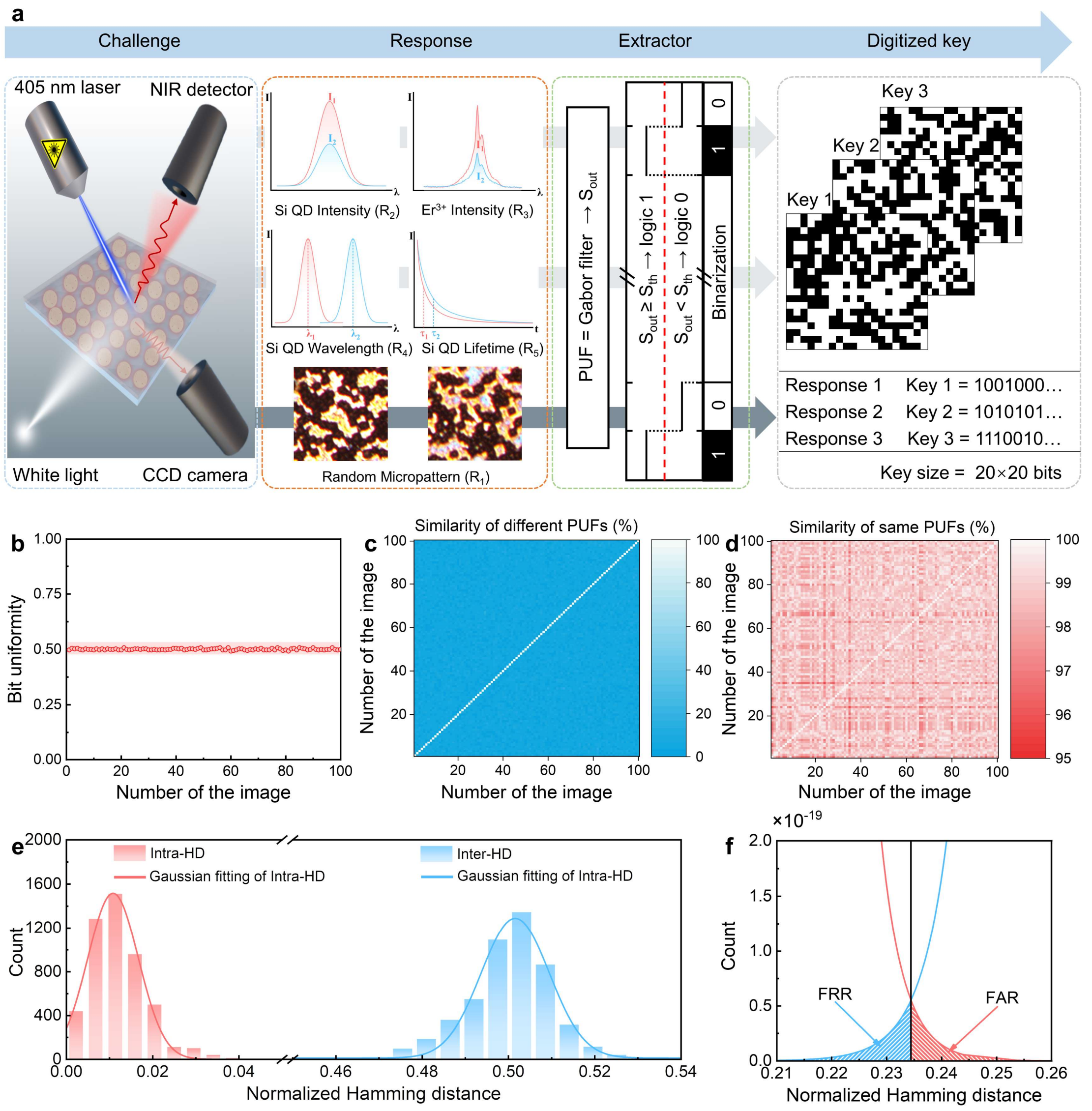

Fig. 4 | Generated multiple security keys and features of a PUF based on the PL wavelength of Si QDs (R_4). a Challenge-response authentication process for the PUF. **b** Occurrence probability of "1" in binary bits extracted from 100 images. **c** Pairwise match of 100 different PUFs using per pixel binary encoding of images acquired from the different positions. **d** Pairwise match of 100 same PUFs using per pixel binary encoding of images acquired from the same position. **e** Distribution of normalized Hamming distance (HD) for Inter-HD and intra-HD. **f** Magnified Inter-HD and Intra-HD.

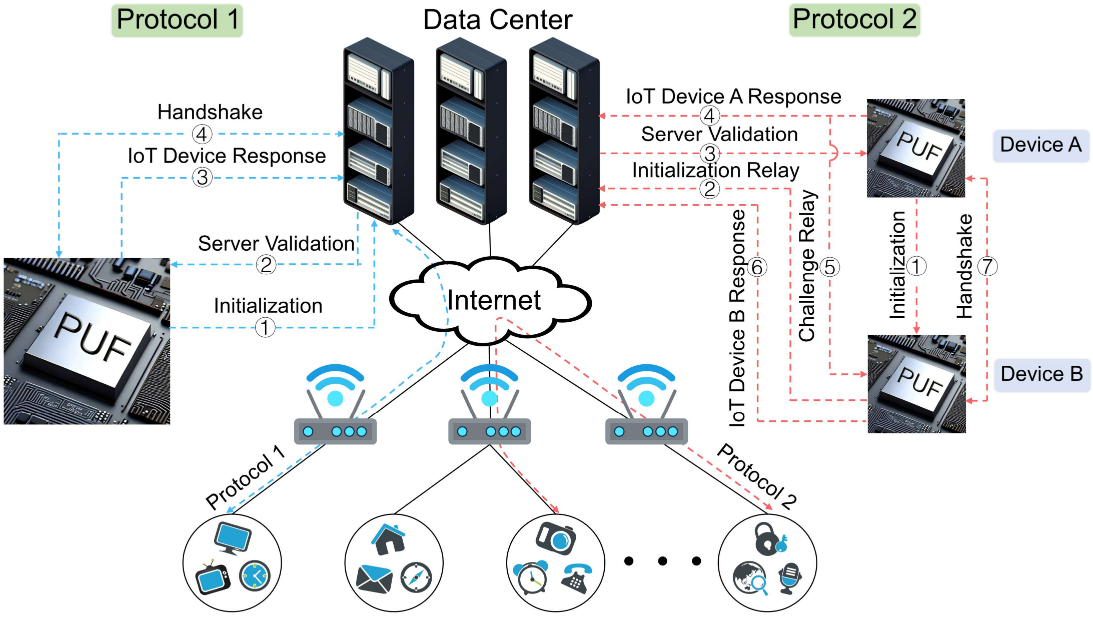

Fig. 5 | Mutual authentication protocols enabled by the all-Si multidimensionally-encoded optical PUF. Protocol 1: device-server mutual authentication. Authentication for the IoT device initiates with the transmission of its ID and a random number to the server (①). The server validates the device, formulates an encrypted message, and sends it back along with codes (②). The device responds, validates the data, and establishes new codes for mutual authentication (③). The server verifies the message, and if successful, mutual authentication is achieved (④). Protocol 2: device-device mutual authentication. The authentication process begins with IoT device A sending its identifier and a random number to IoT device B (①). Device B relays the identifiers and numbers to the server (②). The server retrieves suitable CRPs and generates encrypted messages for device A (③). Device A responds by decrypting messages, verifying integrity, and sending an encrypted message to the server (④). The server updates the associated CRP. A challenge relay occurs (⑤), and device B responds similarly (⑥). Finally, a validation and handshake process takes place, completing the mutual authentication between device A and device B (⑦).

2. We revised the affiliations.

Our revision to the manuscript:

[1]State Key Laboratory of Silicon and Advanced Semiconductor Materials & School of Materials Science and Engineering, Zhejiang University, Hangzhou, Zhejiang 310027, China

[2]CAS Key Laboratory of Standardization and Measurement for Nanotechnology, National Center for Nanoscience and Technology, Beijing 100190, China

[3]State Key Laboratory for Superlattices and Microstructures, Institute of Semiconductors, Chinese Academy of Sciences, Beijing 100083, China

[4]School of Electronic Science and Engineering & National Laboratory of Solid State Microstructures, Nanjing University, Nanjing, Jiangsu 210093; School of Microelectronics, Nantong University, Nantong, Jiangsu 226019, China

[5]Institute of Advanced Semiconductors, ZJU-Hangzhou Global Scientific and Technological Innovation Centre, Zhejiang University, Hangzhou, Zhejiang 311215, China

3. We optimized Table 1 to increase clarity.

Our revision to the manuscript:

Table 1 | The anti-counterfeiting performance and readout accessibility of our PUF for various optical responses.

**Response**	**Bit uniformity**	**Intra-HD**	**Inter-HD**	**FAR**	**FRR**	**Readout**	
						**Time**	**Equipment**
R_1	0.4989	0.0125	0.4989	2.0538×10^{-21}	1.4709×10^{-21}	1 s	Optical microscopy
R_2	0.4987	0.0062	0.5005	2.1088×10^{-36}	5.3115×10^{-36}	40 s	Confocal Raman System
R_3	0.4985	0.0063	0.4998	4.7162×10^{-31}	8.0269×10^{-31}	200 s	
R_4	0.5006	0.0120	0.5004	2.4232×10^{-22}	3.1076×10^{-22}	40 s	
R_5	0.4991	0.0106	0.5017	1.3949×10^{-16}	3.0477×10^{-16}	40 s	

4. We standardized the decimal places to four digits for all numerical values.

   Our revision to the manuscript:

   The PUF in our study possesses a *c* value of 5, consisting of five optical responses. Accordingly, we image an area of 20 × 20 pixels, providing a theoretical encoding capacity of 5^{400} (i.e., 3.8726×10^{279}).

5. We revised the Data Availability section.

   Our revision to the manuscript:

   The authors confirm that data supporting the findings of this study are available within the article and its Supplementary Information. Additional data supporting these findings are available from the corresponding author upon reasonable request. Source data are provided with this paper.

6. We revised the Acknowledgments section.

   Our revision to the manuscript:

   D. R. Y. acknowledges support from the National Key Research and Development Program of China (2018YFB2200101). X. D. P. acknowledges support from the National Natural Science Foundation of China (U22A2075, 61721005), the Fundamental Research Funds for the Central Universities (226-2022-00200). X. F. L. acknowledges support from the National Key Research and Development Program of China (2023YFA1507002), the National Natural Science Foundation of China (22073022), the National Science Foundation for Distinguished Young Scholars of China (22325301). The authors also acknowledge the Instrumentation and Service Center for Molecular Sciences at Westlake University for technical assistance. We also thank Dr. Rong Wang for the invaluable discussion.
